# Conditioned accumbal dopamine transients forecast individual preference for drug versus natural rewards and compulsive behavior

**DOI:** 10.1038/s41593-026-02331-y

**Published:** 2026-06-26

**Authors:** Vincent Pascoli, Laurena Python, Agnès Hiver, Ruud van Zessen, Fabrice Chaudun, Julian Hinz, Jérôme Flakowski, Benoit Girard, Maria Reva, Camilla Bellone, Vahid Esmaeili, Christian Lüscher

**Affiliations:** 1 Department of Basic Neurosciences, Medical Faculty, University of Geneva, Geneva, Switzerland; 2 Synapsy Center for Mental Health Research, Geneva, Switzerland; 3 Clinic of Neurology, Department of Clinical Neurosciences, Geneva University Hospital, Geneva, Switzerland

**Keywords:** Addiction, Motivation

## Abstract

Addicted individuals prefer drug rewards over natural rewards. Dysregulated dopamine (DA) signaling in the nucleus accumbens is thought to trigger this phenomenon, but there is little supporting evidence. Using a genetically encoded sensor, we monitored DA dynamics in mice in an operant associative task with natural (fat solution) or artificial (cocaine or optogenetic DA self-stimulation) rewards. During learning, DA transients emerged at predictive cues for both reward types, vanished at natural reward delivery, but persisted at artificial reward delivery. In choice sessions, animals’ preferences varied from exclusive natural reward to exclusive artificial reward. Individual preferences were predicted by cue-evoked DA response magnitudes. Revaluation of reward contingencies shifted reward-type preference and DA signaling accordingly, but not in mice showing compulsive drug seeking, indicating impaired value updating in these mice. These results support a model in which DA drives adaptive reward seeking, with persistent cue-associated DA signaling underpinning addiction vulnerability.

## Abstract (web summary)

Pascoli et al. show that nucleus accumbens dopamine transients evoked by reward-predictive cues encode subjective reward value in mice, predicting individual preference for drugs over natural rewards and forecasting vulnerability to compulsive drug-seeking behavior.

## Main

Taking drugs at the expense of natural rewards (NRs) is a hallmark of drug addiction^1,2^. This preference bias suggests that artificial rewards (ARs), such as addictive drugs, can hijack the reward system, eventually leading, in some individuals, to compulsive use despite negative consequences. However, studies showing that, in rats, NRs, such as sweets, can be more attractive than cocaine or heroin^3,4^ have challenged this notion. In these studies, only a minority of rats chose drugs at the expense of a sugar solution^5^. The individual preference likely reflects the value assigned to rewards and this value is driven by the context^6–8^. Indeed, multiple factors influence the assignment of subjective value, including the animal’s current physiological state, past experiences and costs such as effort, risk and delay^9,10^.

The delivery of NRs and ARs initially activates the mesolimbic DA system, but this changes with repeated exposure. When a NR becomes predictable, DA neuron activity and DA levels in the nucleus accumbens (NAc) shift from reward delivery (Rd) to predictive cues^11–14^. The dynamics of DA transients during cue–reward association learning resemble the reward prediction error (RPE) signaling in reinforcement learning models^15–17^. In this model, each additional trial generating a DA signal after Rd fuels the DA transient elicited by predictive cues in subsequent trials, increasing the cues’ encoded value and eventually their motivational properties^9,10,18,19^. As addictive drugs invariably elevate DA levels after intake through their distinct pharmacological properties^20^, the DA transients at cue presentation would increase with each iteration^21–25^. A leading theory builds on this difference between NRs and drug rewards and posits that drug-evoked accumbal DA release represents an aberrant reinforcement learning signal^26^. Such hijacking of the reward system would artificially inflate the value attributed to drug-related cues, subsequently biasing decision-making toward drug use^27–30^, because the mismatch between expectation and outcome is maintained^31^.

Mesolimbic DA release can also be controlled using optogenetic effectors expressed in ventral tegmental area (VTA) DA neurons. Operant optogenetic VTA DA neuron self-stimulation (oDASS) produces robust and reinforcing DA transients in the NAc^32–34^, eventually leading to compulsive self-stimulation in some individual mice^35,36^. Thus, oDASS is considered an AR, one that enables precise control over the degree of reinforcement and leverages a fundamental mechanism common to addictive drugs. Like other rewards, oDASS drives the emergence of accumbal DA transients triggered by a predictive cue, including in the NAc core^37–40^. Although compelling, the hypothesis that DA dynamics predict preference for drug rewards over NRs and determine addiction risk has not been empirically tested.

Here we monitored DA dynamics in the NAc using the genetically encoded sensor dLight1.2 while mice learned to self-administer either an NR (highly palatable fat solution) or an AR (cocaine or oDASS). We found that the difference in cue-evoked DA transients (NR − AR) predicted both the subsequent individual reward preference and the emergence of compulsive AR seeking. Specifically, mice with strong responses to AR-predictive cues preferred ARs and were more likely to persevere despite adverse consequences. These findings suggest that an enhanced DA response to drug-associated cues may not only bias drug preference but also serve as an early neural marker of addiction vulnerability.

## Results

### Operant conditioning for NR and AR builds accumbal DA transients that predict individual preference

**Fig. 1:**
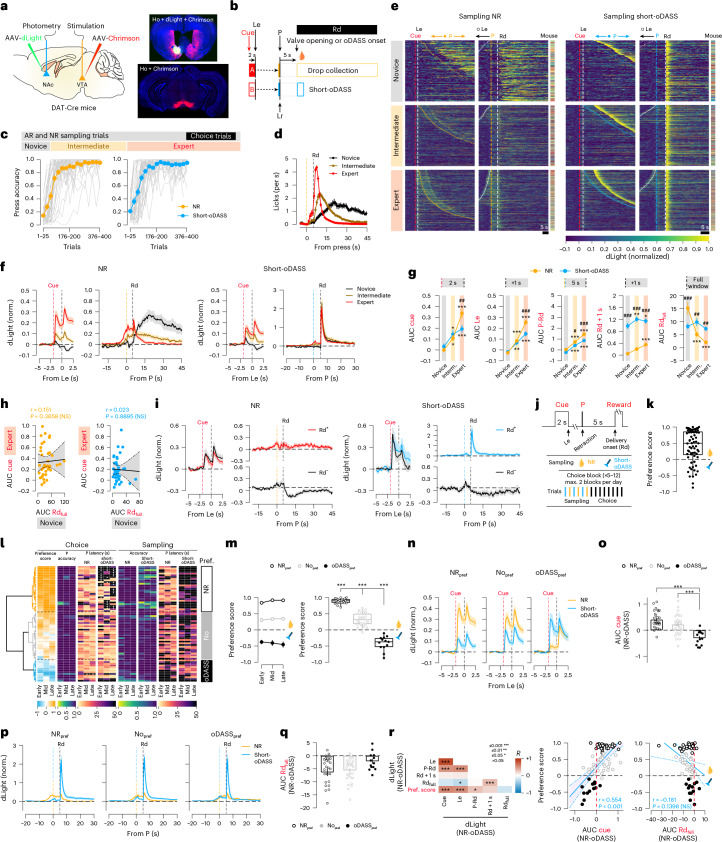
Operant conditioning for NR and AR builds accumbal DA transients that predict individual preference in expert mice. **a**, DAT-cre mice infected with AAV8-hSynFlex-ChrimsonR-tdTomato in the VTA and with the DA sensor, AAV-dLight1.2-egfp in the NAc. Immunohistochemistry against eGFP for amplified dLight visualization is shown in green, of Hoechst for nuclear staining in blue and of VTA DA neurons in the VTA (soma) and the NAc (terminals) in red. **b**, Operant task schedule. Sampling trials started by presenting a specific cue (A or B) preceding an Le (right or left) associated with NR or short-oDASS. The lever press (P) triggered its immediate retraction (Lr) and 5 s later the Rd. Rewards are either a drop of fat solution or short-oDASS (2 bursts of 5 pulses at 20 Hz, 500 ms apart). **c**, Accuracy to P for NR or short-oDASS during the first 400 trials and accuracy to P (*n* = 38 mice). Three learning periods were defined based on P accuracy (Methods). **d**, Number of licks per s around the Rd(*n* = 38 mice). **e**, Normalized dLight signals in hit sampling trials during the three learning periods (2,725 out of 5,579 sampling trials for the NR and 3,343 out of 6,156 for short-oDASS) were recorded from 38 mice sorted according to the P latency. DA transients were aligned either to the Le (white dashed line, 2 s after the cue onset) or to the lever P (orange or blue dashed line, 5 s before the Rd), from −10 s to +45 s or +30 s, for, respectively, NR and short-oDASS. Orange (or blue in short-oDASS trials) dots are for P and white dots are for the Le. **f**, Grouped data of dLight signal around either Le or P for NR and short-oDASS sampling during the three learning periods (*n* = 38). **g**, Mean AUC of normalized dLight during cue and after Le during learning (for cue, two-way repeated measures analysis sof variance (RM ANOVA), main period effect *F*[2,74] = 51.23, *P* ≤ 0.001, *η*^2^_p_ = 0.581 and interaction period × reward type *F*[2,74] = 5.82, *P* = 0.0045, *η*^2^_p_ = 0.136; for Le, two-way RM ANOVA, main period effect *F*[2,74] = 98.21, *P* ≤ 0.001, *η*^2^_p_ = 0.726 and interaction period × reward type *F*[2,74] = 6.20, *P* = 0.0032, *η*^2^_p_ = 0.144; for windows between P and Rd, main period effect *F*[2,74] = 42.86, *P* ≤ 0.001, *η*^2^_p_ = 0.537, main reward-type effect *F*[1,37] = 19.14, *P* ≤ 0.001, *η*^2^_p_ = 0.341; for 1 s immediately after Rd, main period effect *F*[2,74] = 14.13, *P* ≤ 0.001, *η*^2^_p_ = 276 and main reward-type effect *F*[1,37] = 95.90, *P* ≤ 0.001, *η*^2^_p_ = 0.722; for the full-time-window after Rd (Rd_full_), main period effect *F*[2,74] = 28.44, *P* ≤ 0.001, *η*^2^_p_ = 0.435 and interaction period × reward-type effect *F*[2,74] = 27.06, *P* ≤ 0.001, *η*^2^_p_ = 0.429. ANOVAs are followed by Bonferroni’s test: ^*^P ≤ 0.05, ^**^P ≤ 0.01, ^***^P ≤ 0.001, for intermediate or expert versus novice; ^#^*P* ≤ 0.05, ^##^*P* ≤ 0.01, ^###^*P* ≤ 0.001 for NR versus short-oDASS (*n* = 38). **h**, Correlation between the AUC of normalized dLight at cue in expert mice and the maximum AUC after Rd (Rd_full_) in novice mice, for NR and short-oDASS (two-tailed Spearman’s: *r*_36_ = 0.151, *P* = 0.3658, 95% confidence interval (CI) (−0.187, −0.457) and *r*_36_ = 0.023, *P* = 0.8895, 95% CI (−0.308, +0.349], respectively; *n* = 38 mice). **i**, The dLight signal recorded from expert mice during sampling trials with or without Rd (Rd^+^ or Rd^−^). Normalized dLight signal aligned to the Le or the P, with or without the Rd (drop of NR, *n* = 12 or short-oDASS, *n* = 7). **j**, Schedule of the experiment. The experiment consisted of alternating blocks of sampling and choice trials with NR and short-oDASS in expert mice (*n* = 68). Choice trials started with both cues presented simultaneously, followed by the extension of both levers. Lever P led to the retraction of both levers (Lr). Next, the operant task was as previously described for sampling trials. **k**, Distribution of preference score calculated from all choice trials (*n* = 68 mice). **l**, Behavioral clustering based on individual preference score (*n* = 68), P accuracy and latency in sampling and choice trials (white dots indicate missed trials). Data for all choice trials were pooled or presented for three epochs (early, mid and late). **m**, Evolution of the preference score for the three clusters of mice (*n* = 27, 28 and 13 mice) and distribution (one-way ANOVA, *F*[2,65] = 282.8, *P* ≤ 0.001, *η*^2^_p_ = 0.897, followed by Bonferroni’s test: ^***^P ≤ 0.001 for effects between clusters). **n**, Normalized dLight signal aligned to Le for three behavioral clusters of mice. **o**, Quantification of the difference of the normalized dLight signal evoked at cue associated with NR and short-oDASS (one-way ANOVA, *F*[2,65] = 18.37, *P* ≤ 0.001, *η*^2^_p_ = 0.361, followed by Bonferroni’s test: ^***^P ≤ 0.001 for effects between clusters; *n* = 27, 28 and 13 mice). **p**, Normalized dLight signal aligned to P (5 s before Rd) during the sampling trials for short-oDASS and NR for three behavioral clusters of mice. **q**, Quantification of the difference of normalized dLight signal recorded from the full window after Rd (Rd_full_) in NR and short-oDASS sampling trials (no significant effect between clusters, *n* = 27, 28 and 13 mice). **r**, Left: table of correlation between the difference of AUC of normalized dLight (NR-oDASS) for each event of the sampling trial and the preference score measured from later choice trials. Right: correlation between the preference score and the difference (NR-oDASS) of normalized dLight signal evoked at the specific cue or after Rd (two-tailed Spearman’s: *r*_66_ = 0.554, *P* ≤ 0.001, 95% CI (0.357, 0.704) and *r*_66_ = −0.181, *P* = 0.1398, 95% CI (0.408, 0.067), respectively; *n* = 68 mice). Box plots represent the distribution (median represents the 25th and 75th percentiles and whiskers the 5th and 95th percentiles). Data are represented as mean ± s.e.m. Interm., intermediate; max., maximum; Mid, middle; norm., normalized; NS, not significant; Pref., preference.

**Extended Data Fig. 1:**
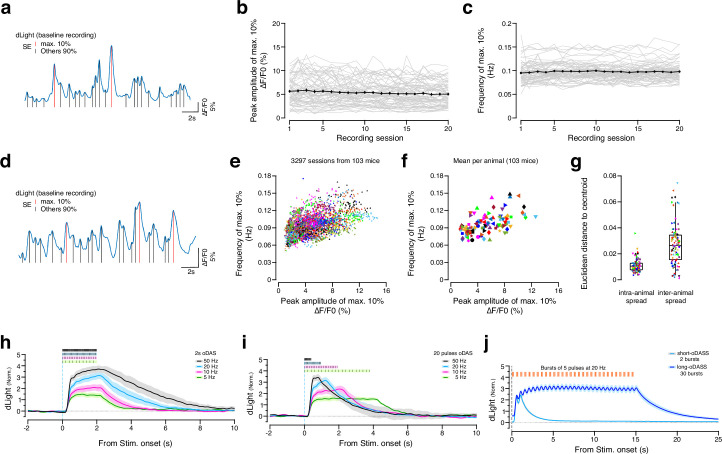
Normalization of dLight signals with Spontaneous events (SE) and optogenetic validation of accumbal dLight signals. **a**, Example trace of baseline dLight recording (ΔF/F0) during the learning phase. The 10% events of maximum amplitude (labelled in red) were detected in aggregate periods outside experimental trial windows and used for normalization of each recording session. **b**, Amplitude of the 10% highest events per mouse from 20 first recording sessions of the learning phase and grouped data in black (one-way RM ANOVA F[19,1273] = 3.39, *P* = 0.0019, η^2^_p_ = 0.048, followed by Dunnett’s test: none of the sessions 2 to 19 were significantly different from session 1, *n* = 68 mice). **c**, Corresponding frequency of 10% highest events (one-way ANOVA F[19,1273] = 1.43, *P* > 0.05, η^2^_p_ = 0.021, *n* = 68 mice). Gray lines are for individual mice and grouped data are represented as mean ± s.e.m. in black. **d**, Example trace of baseline dLight recording (ΔF/F0) from an expert mouse. The 10% events of maximum amplitude (labelled in red) were detected in aggregate periods outside experimental trial windows. **e**, Scatter plot of the 10% highest SE (frequency versus amplitude) measured from 103 expert mice in 3295 sessions. Symbols are for mice identification. **f**, Average per animal (*n* = 103). Distribution of the Euclidean distance to centroid between animals or between sessions. **g**, Box plots represent the distribution (median, 25 and 75%, whiskers are for 5 and 95%, *n* = 103). **h**, Normalized dLight signal in response to passive optogenetic stimulation of VTA dopamine neurons (10 ms pulses) of 2 s with increasing frequencies (*n* = 7). **i**, Normalized dLight signal in response to passive stimulation (10 ms pulses) of 20 pulses at with increasing frequencies (*n* = 7). **j**, Comparison of normalized dLight signal evoked by long- and short-oDASS (*n* = 56 and 54, respectively). Data are represented as mean ± s.e.m.

**Extended Data Fig. 2:**
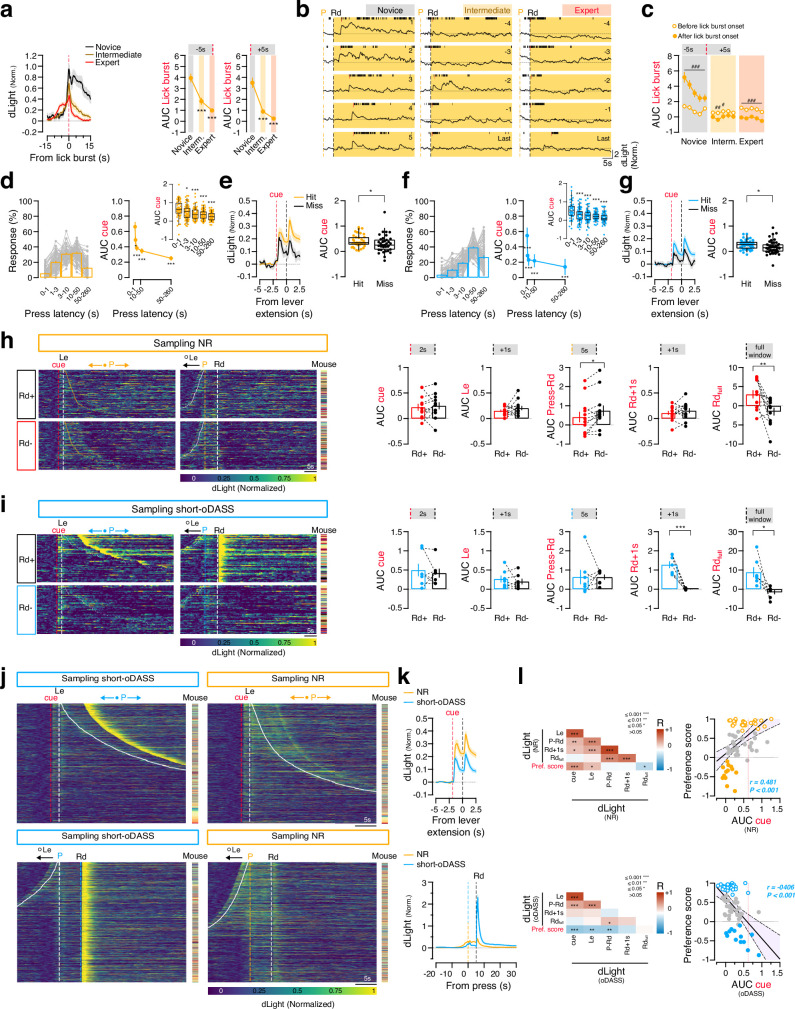
Accumbal dLight signals during sampling trials for NR and short-oDASS. **a**, Left, normalized dLight signal aligned to the onset of the first lick burst after reward delivery (Rd) during the three learning phases. Right, AUC for before and after the lick burst onset for the three learning phases (one-way mixed ANOVA, respectively F[2,74] = 40.94, *P* ≤ 0.001, η^2^_p_ = 0.532 and F[2,74] = 47.54, *P* ≤ 0.001, η^2^_p_ = 0.569, followed by a Dunnett’s test: ***P ≤ 0.001, *n* = 38 mice). **b**, Normalized dLight signal from single trial (first five hit trials of the novice period and then last five hit trials from intermediate and expert periods) from an example mouse. Red mark is for the first lick of the first burst of licks for after the Rd. **c**, Quantifications of the normalized dLight signal around the lick burst in hit trials (two-way RM ANOVA, main trial effect F[14,728] = 15.67, *P* ≤ 0.001, η^2^_p_ = 0.232 and trial x before / after interaction effect F[14,728] = 30.94, *P* ≤ 0.001, η^2^_p_ = 0.373, followed by a Bonferroni test: #*P* ≤ 0.05, ##*P* ≤ 0.01, ###*P* ≤ 0.001, *n* = 53 mice). **d**, Left, distribution of press latencies for NR sampling trials in expert mice (*n* = 68). Right, AUCs of dLight signal for 2 s after the cue onset recorded from expert mice (one-way mixed ANOVA F[4,317] = 11.92, *P* ≤ 0.001, η^2^_p_ = 0.131, followed by Dunnett’s test: *P ≤ 0.05, ***P ≤ 0.001 when compared to 0-1 s press latency, n = 68 mice) as a function of the latency to press. **e**, Left, grouped data of dLight signal aligned to the lever extension (2 s after the cue onset) for hit and miss trials in expert mice (only mice with miss trials were included). Right, AUCs of dLight signal for 2 s after the cue onset in hit and miss trials (Two-tailed paired *t*-test, t34 = 2.13, *P = 0.0408, *n* = 35/68, 272 miss and 3637 hit). **f**, Left, distribution of press latencies for short-oDASS trials in expert mice (*n* = 68 mice). Right, AUCs of dLight signal for 2 s after the cue recorded from expert mice as a function of the latency to press (one-way mixed ANOVA F[4,317] = 10.66, *P* ≤ 0.001, η^2^_p_ = 0.122, followed by Dunnett’s test: ***P ≤ 0.001 when compared to 0-1 s press latency, *n* = 68 mice). **g**, Left, grouped data of normalized dLight signal aligned to the lever extension (2 s after the cue onset) for hit (blue) and miss (black) short-oDASS sampling trials. Only data from mice having missed trials when expert are shown (n = 44/68 mice, 517 miss and 3497 hit). Right, AUCs of normalized dLight signal in hit and miss short-oDASS sampling trials (Two-tailed paired *t*-test, t43 = 2.26, *P = 0.0287, *n* = 44). **h**, Left, Normalized dLight signal recorded from expert mice during sampling trials with or without the natural reward delivery (Rd+ or Rd-). Recordings aligned to the lever extension (white dashed line) or to the press (orange dashed line), data from –15 s to +45 s. Trials are sorted as a function of the delay to press. Orange dots are for press and white dots are for Le (*n* = 12). Right, AUCs of the normalized dLight for each of the five windows (Two-tailed paired *t*-test, t11 = 0.54, *P* = 0.6017; t11 = 1.15, *P* = 0.2759; t11 = 3.65, *P = 0.0108; t11 = 1.14, *P* = 0.2802; t11 = 3.23, **P = 0.0080, *n* = 12 mice). **i**, Left, Normalized dLight signal recorded from expert mice during sampling trials with or without the short-oDASS reward delivery (Rd+ or Rd-). Recordings aligned to the lever extension (white dashed line) or to the press (blue dashed line), data from –15 s to +45 s. Trials are sorted as a function of the delay to press. Blue dots are for press and white dots are for Le (*n* = 7). Right, AUCs of the normalized dLight for each of the five windows (Two-tailed paired *t*-test, t6 = 0.62, *P* = 0.5579; t6 = 0.73, *P* = 0.4931; t6 = 0.02, *P* = 0.9835; t6 = 7.29, ***P = 0.0003; t6 = 3.07, *P = 0.0220, *n* = 7 mice). **j**, Top, normalized dLight signal recorded with photometry during sampling trials for short-oDASS and NR. Recordings of sampling trials for the 68 expert mice were aligned to the lever extension (2 s after the cue onset, -10 to +30 s) and sorted as a function of the delay to press. Orange and blue dots indicate lever presses. Bottom, normalized dLight signal recorded with photometry during sampling trials for short-oDASS and NR. Recordings of sampling trials for the 68 expert mice were aligned to the lever press (5 s before the Rd, -10 to +30 s) and sorted as a function of the delay to press. White dots indicate Le. **k**, Top, grouped data of normalized dLight signal aligned to the lever extension for all mice (*n* = 68). Bottom, normalized dLight signal aligned to press (5 s before Rd) during the sampling trials for short-oDASS and NR recorded from expert mice (*n* = 68). **l**, Top, table of correlation between the AUC of normalized dLight for each event of the natural reward sampling trial and the preference score measured from later choice trials and correlation between the preference score and the AUC of normalized dLight signal evoked at the specific cue associated with NR (Two-tailed Spearman’s: r_66_ = 0.481, *P* ≤ 0.001, 95% CI [0.267,0.650], *n* = 68 mice). Bottom, table of correlation between the AUC of normalized dLight for each event of the short-oDASS sampling trial and the preference score measured from later choice trials and correlation between the preference score and the AUC of normalized dLight signal evoked at the specific cue associated with short-oDASS (Two-tailed Spearman’s: r_66_ = -0.406, *P* ≤ 0.001, 95% CI [-0.593,-0.179], *n* = 68 mice). Box plots represent the distribution (median, 25 and 75%, whiskers are for 5 and 95%). Data are represented as mean ± s.e.m.

To study DA dynamics with photometry during operant self-stimulation, we expressed Cre-dependent opsin (AAV8-hSynFlex-ChrimsonR-tdTomato) in the VTA of DAT-Cre mice and the DA sensor AAV-dLight1.2 in the NAc (Fig. 1a). Mice were trained in parallel sampling trials to collect either an NR (drop of fat solution) or an AR (short-oDASS, optogenetic stimulation of VTA dopamine neurons which consisted of 2 bursts with 5 pulses at 20 Hz, 500 ms apart). Each sampling trial started with the cue presentation for 2 s that was terminated by the lever extension (Le), which was retracted once the animal pressed it (press (P)). P triggered the Rd after a delay of 5 s (Fig. 1b). The mice were not food restricted and only a limited number of daily trials were conducted to avoid satiation. To account for slight differences in interindividual expression levels or photometry efficiency, we normalized the DA signals to the amplitude of the largest spontaneous events of each recording (Extended Data Fig. 1a–g and Methods). The dLight recordings in the NAc while stimulating, optogenetically, VTA DA neurons were performed to define appropriate protocols and confirmed independence between opsin and sensor (Extended Data Fig. 1h–j and Methods). During the first 20 sampling sessions, the mice increased their accuracy to press (up to >75%) similarly for NR and short-oDASS and became experts (Fig. 1c and Methods). With learning the operant NR sampling task, licking behavior progressively moved closer to the Rd to immediately collect the drop and anticipatory licking appeared (Fig. 1d). The dLight measurements recorded in hit trials were aligned either to Le or to the P. For NR, DA transients were strongest after Rd in the novice, decreased rapidly with learning and disappeared in experts. Conversely, DA transients emerged at the distal cue during intermediate trials and became robust in expert mice (Fig. 1e–g). DA transients were first detected after the burst of lick following the NR delivery but then were visible before lick onset, likely driven by reward expectation or the incidental sound of the solenoid (Extended Data Fig. 2a–c). The DA transient evoked by the optogenetic stimulation (short-oDASS) persisted in expert mice but similarly developed at cue during the learning periods (Fig. 1e–g). In expert mice, DA transients evoked by the distal cue were higher in short-latency trials and hit trials compared to, respectively, long-latency or miss trials for both NR and short-oDASS (272 miss and 3,637 hit trials for NR, 517 miss and 3,637 hit trials for short-oDASS; Extended Data Fig. 2d–g), suggesting a potential motivational role in a trial-by-trial manner.

**Extended Data Fig. 3:**
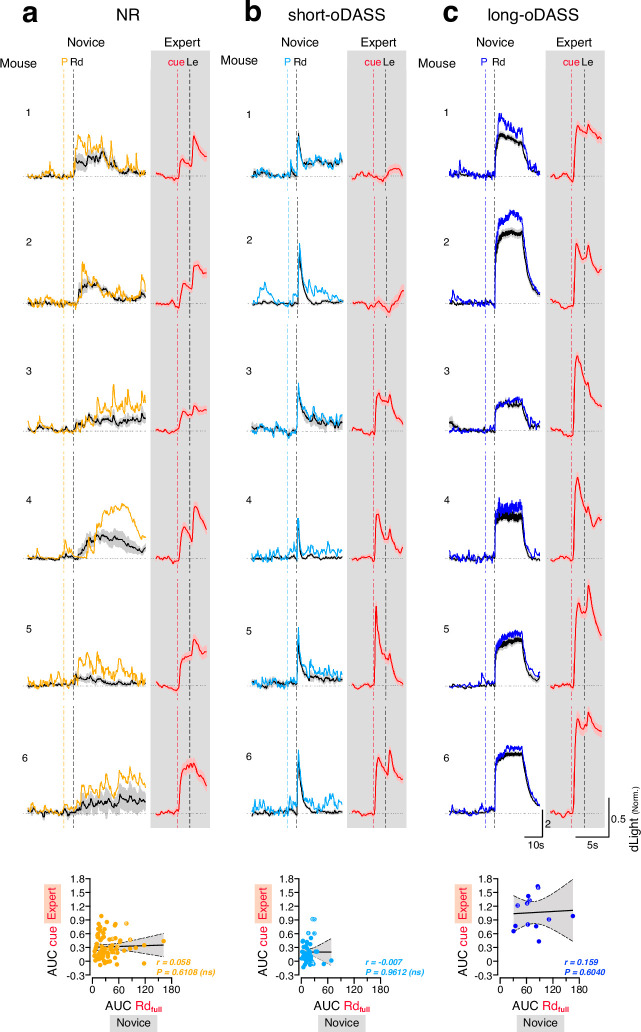
Lack of individual correlations between reward-evoked dopamine in novice and cue-evoked dopamine expert mice. **a**, Mean traces (black) across novice trials and trace of the maximum dLight signal trial (orange) recorded in the novice stage, aligned to the lever press for 6 example mice. Average traces of dLight signal, aligned to the lever extension recorded for the corresponding mice when becoming expert. Bottom, AUC of normalized dLight signal during cue presentation in expert mice as a function of the AUC of normalized dLight signal after the reward delivery (Rd) from the same mice, when novice (Two-tailed Spearman’s: r_78_ = 0.058, *P* = 0.6108, 95% CI [-0.171,0.280], *n* = 80 mice). Numbers are for example mice. **b**, Same as (a) for short-oDASS (Two-tailed Spearman’s: r_45_ = -0.007, *P* = 0.9612, 95% CI [-0.302,0.289], *n* = 47 mice). **c**, Same as (b) for long-oDASS (Two-tailed Spearman’s: r_11_ = 0.159, *P* = 0.6040, 95% CI [-0.444,0.663], *n* = 13 mice). Data are represented as mean ± s.e.m.

Additional analysis further strengthened the observation that DA transients emerge at predictive stimuli, such as the distal cue, the Le or valve opening in NR trials. However, the amplitudes of DA transients evoked by the cues in the expert period did not correlate with the initial amplitudes observed after Rd during the novice period (Fig. 1h and Extended Data Fig. 3). This observation suggests that, although emerging, DA at cue acquires additional properties. We next tested the effects of NR or AR omission with randomly interleaving delivery and omission trials. In reward omission trials, DA signal decreased after the expected delivery of the NR or short-oDASS, in line with previous observations^14^, which were attributed to a negative RPE encoded by VTA DA neurons. In contrast, the DA transient evoked by the cue was similar in rewarded and unrewarded trials (Fig. 1i and Extended Data Fig. 2h,i). Together, these results show that the DA transients shifted from the reward detection initially occurring very close to the Rd to the most distal predictive cue. Individual variability of DA transient at cue, at the end of the learning phase, was observed for both NR and short-oDASS. We also confirmed that DA dips when an expected NR or AR is omitted.

We next assessed individual preferences between NR and AR by offering blocks of choice trials intermingled in sampling trials for each reward (Fig. 1j and Methods). For choice trials, the two levers and two cues were presented simultaneously and both levers were retracted after the choice P. DA transients presented are from sampling trials and not from choice trials. On average, DA transients at cue were slightly higher for NR than for short-oDASS (1.9× area under the curve (AUC)), whereas DA transients after the Rd were much smaller for NR than for short-oDASS (Extended Data Fig. 2j,k). We found a strong interindividual variability of the preference score ((*N*_NR_ − *N*_AR_)/(*N*_NR_ + *N*_AR_), where *N*_NR_ is the number of NRs obtained during choice trials and *N*_AR_ the number of ARs (oDASS or cocaine); Fig. 1k) and proceeded to classify choice strategies with an unsupervised clustering method based on various behavioral parameters, including individual preference scores, press accuracy and latency (Fig. 1l). Three distinct profiles are revealed. Some mice showed a preference for the NR (NR_pref._, 39.7%), whereas other mice remained undecided (No_pref._, 41.2%) or preferred short-oDASS (oDASS_pref._, 19.1%). The preference score remained stable along the blocks of choice trials and highlighted interindividuals’ variability (Fig. 1m). The difference between the DA transients evoked by the cues associated with NR and short-oDASS was different in the three clusters. Mice with NR preference had a higher DA transient for the cue associated with the NR than with the short-oDASS and conversely for mice with the short-oDASS preference (Fig. 1n,o). A small proportion of mice showed a preference for short-oDASS over the NR and a higher DA transient at the associated cue, indicating that some individuals within the population preferentially attributed high value to the AR, despite comparable levels of optogenetically evoked accumbal DA.

Indeed, DA transients after the Rd did not differ across the three clusters of mice (Fig. 1p,q). Finally, we correlated the individual preference score and the difference between NR and short-oDASS transients. We found a strong correlation with DA transients at distal cues but not after Rd during sampling trials. DA transients aligned to the Le also correlated with the preference but most likely because of prior cue-evoked DA (Fig. 1r). The individual preference scores also correlated with DA transient at cue associated with NR or short-oDASS (Extended Data Fig. 2i). Together these data highlight the strong correlation between the individual average DA value at cue and the individual preference between NR and AR.

### Revaluation of AR boosted cue-evoked DA transients and biased preference

**Fig. 2:**
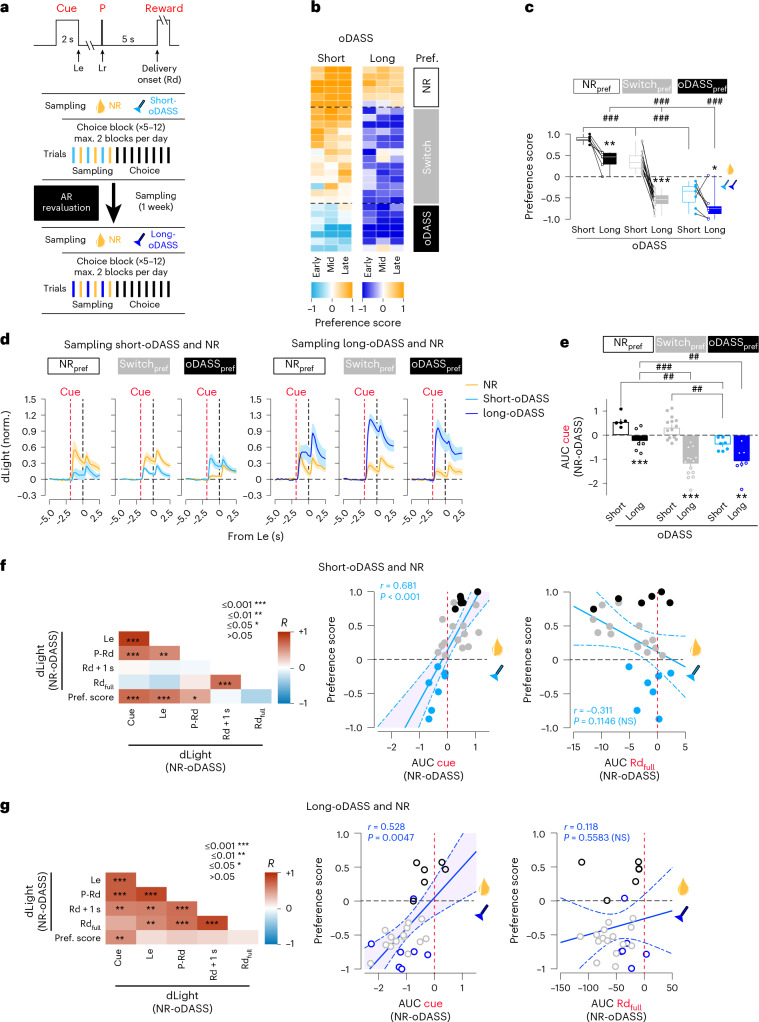
Revaluation of oDASS boosts cue-evoked DA transient and bias preference. **a**, Schedule of experiment. The experiment consisted of two successive phases, beginning with short-oDASS and then long-oDASS (*n* = 27 mice). Between the two phases mice were trained with long-oDASS for a week. **b**, Evolution of individual preference score and behavioral clusters of mice (*n* = 6, 14 and 7 mice; Extended Data Fig. 5). **c**, Preference score distribution (two-way RM ANOVA, main cluster effect *F*[2,24] = 81.17, *P* ≤ 0.001, *η*^2^_p_ = 0.871; oDASS protocol effect *F*[1,24] = 69.09, *P* ≤ 0.001, *η*^2^_p_ = 0.742 and interaction cluster × oDASS protocol *F*[2,24] = 10.52, *P* = 0.0005, *η*^2^_p_ = 0.467, followed by Bonferroni’s test: ^*^P ≤ 0.01, ^**^P ≤ 0.01, ^***^P ≤ 0.001 for short-oDASS versus long-oDASS; ^###^*P* ≤ 0.001 for effects between clusters; *n* = 6, 14 and 7 mice). Box plots represent the distribution (median represents the 25th and 75th percentiles and whiskers the 5th and 95th percentiles). **d**, Normalized dLight signal aligned to Le for three clusters, with short-oDASS or long-oDASS. **e**, Quantification of the difference of the normalized dLight signal evoked at cue associated with oDASS and NR, with short-oDASS or long-oDASS (two-way RM ANOVA, main cluster effect *F*[2,24] = 7.87, *P* = 0.0023, *η*^2^_p_ = 0.396; oDASS protocol effect *F*[1,24] = 92.72, *P* ≤ 0.001, *η*^2^_p_ = 0.794 and interaction cluster × oDASS protocol *F*[2,24] = 7.80, *P* = 0.0025, *η*^2^_p_ = 0.394, followed by Bonferroni’s test: ^**^P ≤ 0.01, ^***^P ≤ 0.001 for short-oDASS versus long-oDASS; ^##^*P* ≤ 0.01, ^###^*P* ≤ 0.001 for effects between clusters; *n* = 6, 14 and 7 mice). **f**, Left: table of correlation between the difference of AUC of normalized dLight (NR-oDASS) calculated for the different events of the trial and the preference score measured from later choice trials. Middle and right: correlation between the preference score and the difference (NR-oDASS) of normalized dLight signal evoked at the cue or after Rd (two-tailed Spearman’s: *r*_25_ = 0.681, *P* ≤ 0.001, 95% CI (0.396, 0.846) and *r*_25_ = -0.311, *P* = 0.1146, 95% CI (−0.625, +0.090), respectively; *n* = 27 mice). **g**, Same as **f** for the second phase of the experiment with oDASS revaluation (two-tailed Spearman’s: *r*_25_ = 0.528, *P* = 0.0047, 95% CI (0.173, 0.761]) and *r*_25_ = 0.118, *P* = 0.5583, 95% CI (−0.285, +0.486), respectively; *n* = 27 mice). Data are represented as mean ± s.e.m.

**Extended Data Fig. 4:**
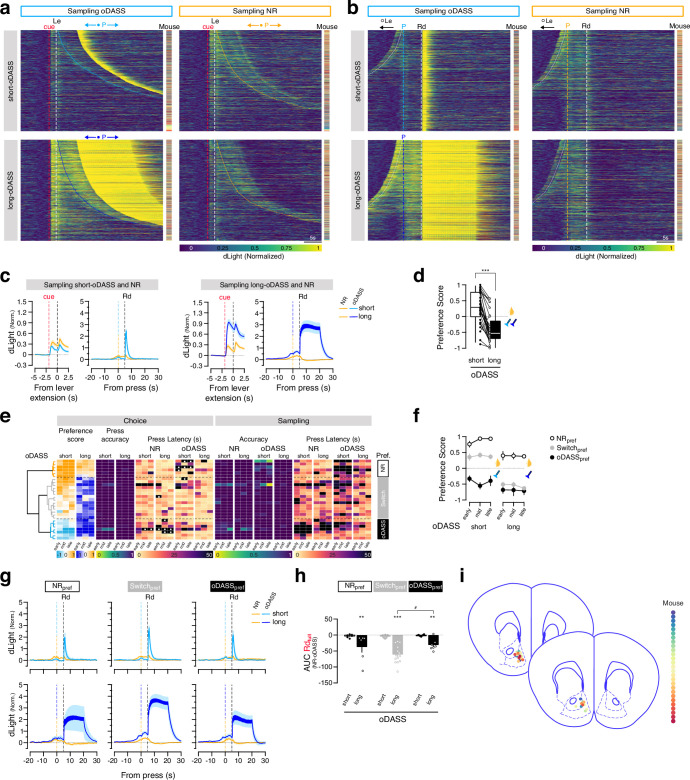
Behavioral clustering and accumbal dLight signal during oDASS revaluation. **a**, Normalized dLight signal recorded with photometry during sampling trials for short-oDASS and NR, and then for long-oDASS and NR. Recordings of sampling trials sorted as a function of the delay to press for the 27 expert mice were aligned to the lever extension (white dashed line, Le, 2 s after the cue onset, -10 to +30 s). Orange and blue dots indicate lever presses. **b**, Same as (A) but with trials aligned to the lever press (orange or blue dashed line, P, 5 s before the reward delivery). White dots indicate Le. **c**, Normalized dLight signal aligned to the lever extension (2 s after cue onset) or to the press (5 s before Rd) during the sampling trials for oDASS and NR recorded from the 27 expert mice, with short- or long-oDASS. **d**, Preference score before and after oDASS revaluation (Two-tailed paired *t*-test, t26 = 7.93, ***P ≤ 0.001, for long- *versus* short-oDASS, *n* = 27). Box plots represent the distribution (median, 25 and 75%, whiskers are for 5 and 95%). **e**, Behavioral clustering based on individual preference score (*n* = 27 mice), press accuracy and latency in sampling and choice trials (white dots when only missed trials). **f**, Evolution of the preference score for the three clusters of mice (*n* = 6, 14 and 7 mice). **g**, Normalized dLight signal aligned to press (5 s before the Rd) during the sampling trials for AR and NR recorded from the 3 clusters of mice, with short- or long-oDASS. **h**, Quantification of the difference of normalized dLight signal recorded from the full time window after the reward delivery in oDASS and NR sampling trials, with short- or long-oDASS (two-way RM ANOVA, main cluster effect F[2,24] = 2.87, *P* = 0.0763, η^2^_p_ = 0.193; main oDASS protocol effect F[1,24] = 53.89, *P* ≤ 0.001, η^2^_p_ = 0.692 and interaction cluster x oDASS protocol F[2,24] = 2.82, *P* = 0.0796, η^2^_p_ = 0.190, followed by a Bonferroni test: **P ≤ 0.01, ***P ≤ 0.001 for short- *versus* long-oDASS; #*P* ≤ 0.05 for effects between clusters, *n* = 6, 14 and 7 mice). **i**, Coronal sections (antero-posterior coordinates +1.54 and + 1.70 from bregma) containing the Nucleus Accumbens are shown. Dots are for the center of the dLight infection for each mouse. Data are represented as mean ± s.e.m.

We next gave a subset of mice that had established a preference between NR and short-oDASS the opportunity to sample and choose long-oDASS (30 instead of 2 bursts) (Fig. 2a). After revaluation, DA transients in the long-oDASS sampling trials became much larger after the Rd, but also at cue presentation, whereas DA transients in the NR sampling trials remained unchanged (Extended Data Fig. 4a,b). The DA transients at cue, typically slightly higher (1.7× AUC), when associated with NR rather than with short-oDASS, were now much higher (4× AUC) on average when associated with long-oDASS (Extended Data Fig. 4c). Accordingly, the preference score was also reversed (Extended Data Fig. 4d). Nevertheless, unsupervised clustering revealed three distinct behavioral profiles: 22.2% mice kept choosing the NR preferentially when oDASS became long (NR_pref._), 51.8% mice shifted their preference toward oDASS after revaluation (Switch_pref._) and 25.9% mice preferred oDASS regardless of the stimulation duration (oDASS_pref._) (Fig. 2b,c and Extended Data Fig. 4e,f). DA transients at cue during sampling trials were initially stronger for the NR than for the short-oDASS in the two groups, choosing the NR preferentially. After revaluation, DA transients at long-oDASS cue became larger, particularly for the two groups that, respectively, reinforced or developed a marked preference for long-oDASS over NR (Fig. 2d). Therefore, the difference of the DA transients evoked by the two cues recorded in interleaved oDASS and NR sampling trials, before and after revaluation, correlated with the choice trajectories. In mice switching and mice with a preference for oDASS, the difference of DA transients between the cues (NR–AR) became negative whereas the difference remained positive for mice preferring NR over long-oDASS (Fig. 2e). DA transients after the Rd similarly increased with long-oDASS for the three clusters of mice (Extended Data Fig. 4g,h). The dLight was expressed in the NAc of all mice (Extended Data Fig. 4i). As shown previously, the individual preference scores correlated with the difference in DA transients between NR and short-oDASS at the discriminative cues, but not after the Rd. Correlation remained strong after revaluation despite the shift toward long-oDASS preference and higher DA transient at associated cue in many mice (Fig. 2f,g). These findings show that increasing AR strength (oDASS burst number) enhances associated cue-evoked DA and shifts preference, but whether similar changes can emerge gradually through extended exposure to the less reinforcing AR remained unclear.

### Extended short-oDASS training failed to enhance value coding or shift preference

**Fig. 3:**
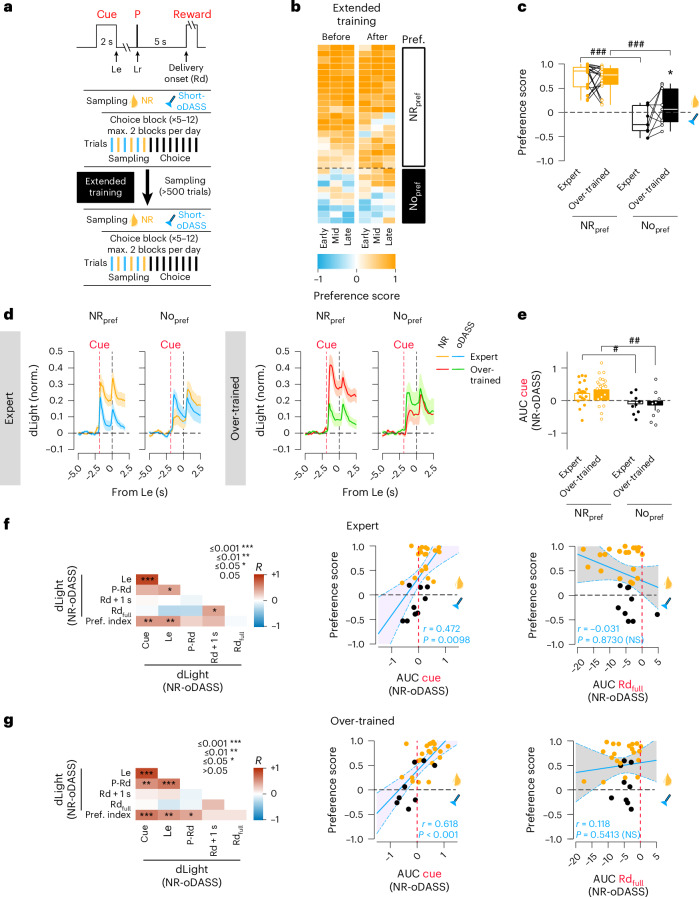
Established value coding and preference remain stable despite extended oDASS training. **a**, Schedule of experiment. The experiment consisted of two recording phases with short-oDASS (*n* = 29 mice). Between the two phases, mice received extended training with short-oDASS (minimum of 500 trials in 1 month). **b**, Evolution of individual preference score and behavioral clusters of mice (*n* = 20 and 9 mice; Extended Data Fig. 6). **c**, Preference score distribution (two-way RM ANOVA, main cluster effect *F*[1,27] = 70.42, *P* ≤ 0.001, *η*^2^_p_ = 0.723 and interaction cluster × extended training *F*[1,27] = 4.58, *P* = 0.0416, *η*^2^_p_ = 0.145, followed by Fisher’s least significant difference (LSD) test: ^*^P ≤ 0.05 for between over-trained versus expert, *n* = 9; ^###^*P* ≤ 0.001 for between-cluster effect; *n* = 20 and 9 mice). Box plots represent the distribution (median represents the 25th and 75th percentiles and whiskers the 5th and 95th percentiles). **d**, Normalized dLight signal aligned to Le for two clusters before and after the extended training. **e**, Quantification of the difference of the normalized dLight signal evoked at cue associated with oDASS and NR, before or after extended exposure (two-way RM ANOVA, main cluster effect *F*[1,27] = 9.21, *P* = 0.0053, *η*^2^_p_ = 0.254, followed by a Fisher’s LSD test: ^#^*P* ≤ 0.05, ^##^*P* ≤ 0.01 for between-cluster effect; *n* = 20 and 9 mice). **f**, Left: table of correlation between the difference of AUC of normalized dLight (NR-oDASS) calculated for the different events of the trial and the preference score measured from later choice trials. Middle and right: correlation between the preference score and the difference of normalized dLight signals evoked at the specific cue or after the Rd (two-tailed Spearman’s: *r*_27_ = 0.472, *P* = 0.0098, 95% CI (0.116, 0.720) and *r*_27_ = -0.031, *P* = 0.8730, 95% CI (−0.403, +0.349), respectively; *n* = 29 mice). **g**, Same as **f** for the second phase of the experiment, after extended training with short-oDASS (two-tailed Spearman’s: *r*_27_ = 0.618, *P* = 0.0004, 95% CI (0.315, 0.807) and *r*_27_ = 0.118, *P* = 0.5413, 95% CI (−0.270, +0.474), respectively; *n* = 29 mice). Data are represented as the mean ± s.e.m.

**Extended Data Fig. 5:**
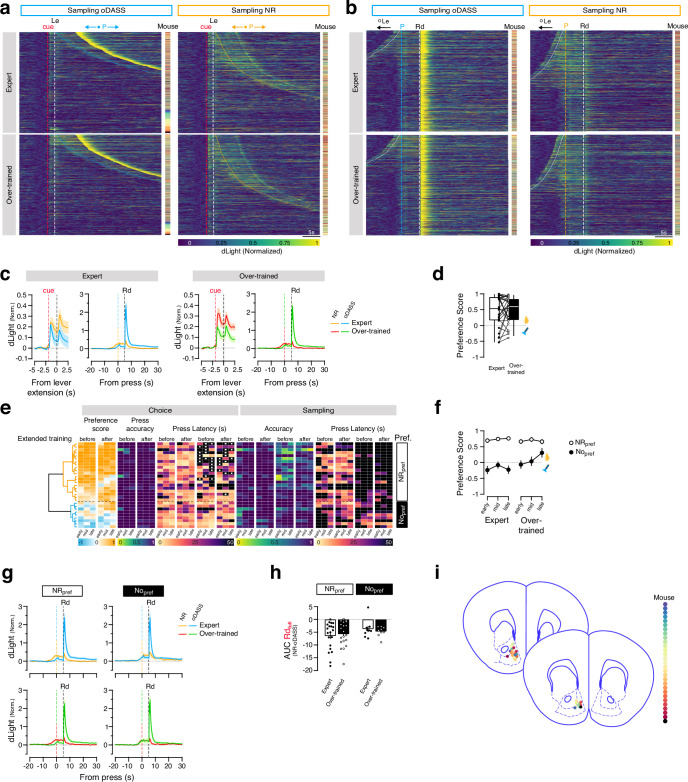
Behavioral clustering and accumbal dLight for short-oDASS and natural reward extended training. **a**, Normalized dLight signal recorded with photometry during sampling trials for short-oDASS and NR, before and after extended exposure. Recordings of sampling trials sorted as a function of the delay to press for the 29 expert mice were aligned to the lever extension (white dashed line, Le, 2 s after the cue onset, -10 to +30 s). Orange and blue dots indicate lever presses. **b**, Same as (A) but with trials aligned to the lever press (orange or blue dashed line, P, 5 s before the reward delivery). White dots indicate Le. **c**, Normalized dLight signal aligned to the lever extension (2 s after the cue onset) or to the press (5 s before Rd) during the sampling trials for oDASS and NR recorded from the 29 expert mice, before and after extended exposure. **d**, Preference score before and after extended exposure (Two-tailed paired *t*-test, t28 = 0.68, *P* = 0.499 (ns), for over-trained *versus* expert, *n* = 29 mice). Box plots represent the distribution (median, 25 and 75%, whiskers are for 5 and 95%). **e**, Behavioral clustering based on individual preference score (*n* = 29 mice), press accuracy and latency in sampling and choice trials (white dots when only missed trials). **f**, Evolution of the preference score for the two clusters of mice (*n* = 20 and 9 mice). **g**, Normalized dLight signal aligned to press (5 s before the reward delivery) recorded from the two clusters of mice during the sampling trials for short-oDASS and NR, before or after extended exposure. **h**, Quantification of the difference of normalized dLight signal recorded from the full window after Rd in short-oDASS and NR sampling trials, before or after extended exposure (two-way RM ANOVA, no significant effects of clusters or extended training were found, *n* = 20 and 9). **i**, Coronal sections (antero-posterior coordinates +1.54 and + 1.70 from bregma) containing the nucleus accumbens are shown. Dots are for the center of the dLight infection for each mouse. Data are represented as mean ± s.e.m.

Therefore, a second subset of mice underwent extended training with the short-oDASS to assess whether prolonged exposure alone could shift preference away from the NR. This experiment probed the prediction that persistent RPE with an AR would result in excessive cue-evoked DA transients^26,28,29^. The extended training period consisted of >500 trials of short-oDASS and NR over 4 weeks, after which the preference was again assessed (Fig. 3a). DA transients were similar before and after extended training. The DA transients at cue slightly higher (1.7× AUC), when associated with NR rather than with short-oDASS, were still higher (1.9× AUC) on average after extended training (Extended Data Fig. 5a–c). The individual preference ranged from strong NR preference to moderate short-oDASS preference, before and after extended training (Extended Data Fig. 5d). The behavioral clustering indicated two groups, one with a preference for the NR (NR_pref._, 69.0%) and another without preference (No_pref._, 31.0%). Surprisingly, mice that showed no preference before extended training shifted weakly toward a preference for the NR after extended training (Fig. 3b,c and Extended Data Fig. 5e,f). The DA transients for the NR-associated cue in mice with NR preference were larger than the DA transients measured at the cue associated with short-oDASS. An opposite pattern was found in mice without a distinct preference, confirming the correlation between individual preference and DA transient difference at cues in sampling trials (Fig. 3d). Of importance, DA transients at discriminative cues did not change with extended training (Fig. 3e). DA transients after the Rd did not differ between mice with and mice without NR preference and remained unchanged despite extended training (Extended Data Fig. 5g,h). The dLight was expressed in the NAc of all mice (Extended Data Fig. 5i). Before and after extended training, individual preference scores were best predicted by the difference between DA transients at cues, whereas no correlation was found with DA transients after Rd (Fig. 3f,g).

In summary, extended exposure to an AR capable of evoking persistent DA release in the NAc failed to enhance DA transient’s amplitude at cue, suggesting that consistently elevated DA after Rd alone doesn’t change the encoded value.

### Cocaine yielded strong cue-evoked DA transients that correlate with preference

**Fig. 4:**
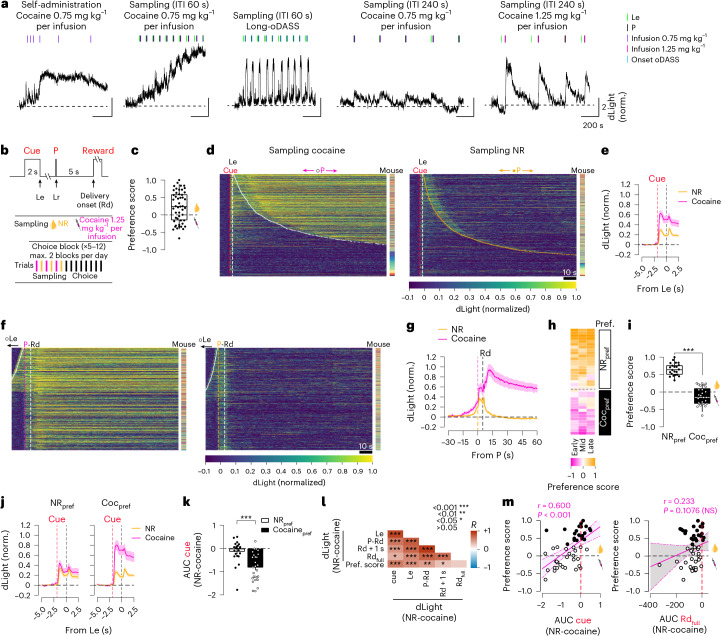
Cocaine-associated cues elicit DA transients that scale with individual preference. **a**, Left to right: normalized dLight signal during self-administration (fixed ratio schedule 1) of cocaine 0.75 mg kg^−1^ per infusion, during sampling trials for cocaine or long-oDASS with a 60-s intertrial interval (ITI) and during sampling trials for cocaine (0.75 or 1.25 mg kg^−1^ per infusion) with a 240-s ITI. **b**, Schedule of experiments. Photometry recordings were done during sampling trials and evaluation of preference between the NR and intravenous cocaine (1.25 mg kg^−1^ per infusion) was done in choice trials (*n* = 49 mice). **c**, Preference score distribution (*n* = 49 mice). **d**, Normalized dLight signal recorded with photometry during sampling trials for cocaine (left) and NR (right). Recordings of sampling trials were sorted as a function of the delay to P were aligned to the Le (white dashed line, 2 s after the cue onset, −10 to +120 s). Orange and white dots indicate lever Ps. Missed trials are shown at the bottom (*n* = 49 mice). **e**, Normalized dLight signal aligned to the Le (2 s after cue onset) during sampling trials for cocaine and NR (*n* = 49 mice). **f**, Same as **d** but with trials aligned to the lever P (orange or pink dashed line, 5 s before the Rd). White dots indicate Le. **g**, Normalized dLight signal aligned to the P (5 s before Rd) during sampling trials for cocaine and NR (*n* = 49 mice). **h**, Evolution of individual preference score and behavioral clusters of mice (*n* = 22 and 27 mice; Extended Data Fig. 7). **i**, Preference score distribution (two-tailed, unpaired Student’s *t*-test, *t*47 = 11.98, ^***^P ≤ 0.001, *n* = 22 and 27 for NR versus cocaine preference). **j**, Normalized dLight signal aligned to the Le for two clusters. **k**, Quantification of the difference of the normalized dLight signal evoked at cue associated with NR and cocaine (two-tailed, unpaired Student’s *t*-test, *t*47 = 4.29, ^***^P ≤ 0.001, *n* = 22 and 27 for NR versus cocaine preference). **l**, Table of correlation between the difference of AUC of normalized dLight (NR-cocaine) calculated for the different events of the sampling trial and the preference score measured from later choice trials. **m**, Correlation between the preference score and the difference of normalized dLight signal evoked at the cue or after Rd (two-tailed Spearman’s: *r*_47_ = 0.600, *P* ≤ 0.001, 95% CI (0.376, 0.758) and *r*_47_ = 0.233, *P* = 0.1076, 95% CI (−0.600, +0.489), respectively; *n* = 49 mice). Box plots represent the distribution (median represents the 25th and 75th percentiles and whiskers the 5th and 95th percentiles). Data are represented as the mean ± s.e.m. Coc, cocaine.

**Extended Data Fig. 6:**
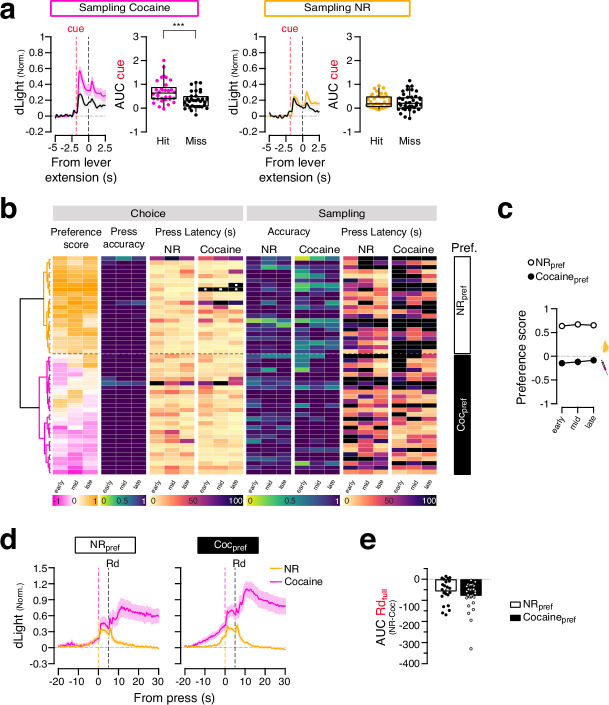
Behavioral clustering and accumbal dLight for cocaine *versus* natural reward preference. **a**, Grouped data aligned to the lever extension (2 s after the cue onset) and AUCs of dLight signal for hit (pink or orange) and miss (black) sampling trials for cocaine (1.25 mg/kg/infusion) and NR (Two-tailed paired *t*-test, t31 = 5.10, ***P ≤ 0.001, and t37 = 0.45, *P* = 0.6553, for cocaine and NR respectively). Only mice having miss trials were included (*n* = 32/49, 288 miss and 922 hit and *n* = 38/49 mice, 353 miss and 2354 hit, for cocaine and NR respectively). **b**, Behavioral clustering based on individual preference score, press accuracy and latency in sampling and choice trials for NR and cocaine (*n* = 49 mice) (white dots when only missed trials). **c**, Evolution of the preference score for the two clusters of mice (*n* = 22 and 27 mice). **d**, Grouped data of normalized dLight signal aligned to the press (5 s before the reward delivery) during the sampling trials for cocaine and NR (*n* = 22 and 27 mice, respectively). **e**, Quantification of the difference of normalized dLight signal AUCs recorded from the full-time window after the reward delivery (after Rd_full_) in cocaine and NR sampling trials (Two-tailed unpaired *t*-test, t47 = 1.14, *P* = 0.2592, *n* = 22 and 27 mice). Box plots represent the distribution (median, 25 and 75%, whiskers are for 5 and 95%). Data are represented as mean ± s.e.m.

Next, to examine whether the DA signatures linked to preference and cue value also apply to a pharmacological AR, we first trained mice to self-administer intravenous cocaine. In self-administration under a fixed ratio schedule of 1, mice typically made successive infusions, resulting in an accumulation of DA, which they maintained with regular Ps. In sampling trials with short delays imposed, DA accumulation also occurred, contrary to long-oDASS. Finally, with longer time intervals between trials, a large DA transient was observed after a single cocaine infusion of 1.25 mg kg^−1^ (Fig. 4a). Mice trained to self-administer intravenous cocaine (1.25 mg kg^−1^ per infusion) were then used to measure accumbal DA during sampling trials alongside NR consumption and their individual preference was assessed (Fig. 4b). The choice between cocaine and NR varied between individuals (Fig. 4c). In interleaved cocaine sampling trials, DA increased after cue presentation and stayed high for minutes when the animal quickly pressed the lever. When mice pressed the lever after a delay (>10–15 s), the DA evoked at the cue had returned to baseline but increased again around the P and after the cocaine infusion. In interleaved NR sampling trials, the pattern was as described previously. Overall, DA transients at cocaine-associated cue were larger (2.4× AUC) than at NR-associated cue (Fig. 4d,e) and were significantly higher in hit than in miss trials for cocaine but not for NR (288 miss and 922 hit for cocaine; 353 miss and 2,354 hit for NR sampling; Extended Data Fig. 6a). As expected, a cocaine-evoked DA transient after the Rd was observed even in expert mice (Fig. 4f). Of note, cocaine produced a DA surge several seconds after the intravenous infusion onset (Fig. 4g). We identified two clusters of about equal size: mice that preferred the NR (NR_pref._, 44.9%) and mice that preferred cocaine (Coc_pref._, 55.1%). The preference was stable across mice from the same cluster (Fig. 4h,i and Extended Data Fig. 6b,c). Mice with a preference for cocaine exhibited the largest difference between DA transients at cues (Fig. 4j,k). In all mice, the DA transients observed after the Rd were much larger in cocaine than in NR trials (Extended Data Fig. 6d,e). The individual preference was best predicted by the difference of DA transients at cues and, to a lesser extent, by the difference after the Le and during the interval P-reward delivery (P-Rd) but not after the Rd (Fig. 4l,m).

**Extended Data Fig. 7:**
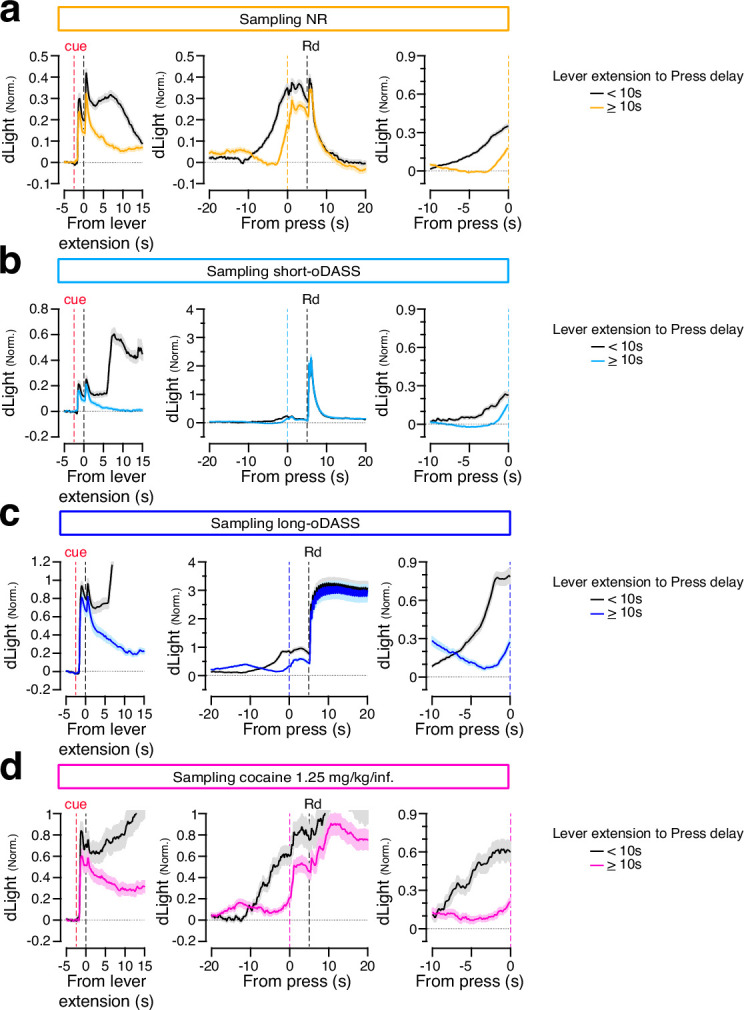
Ramping of dLight signals before lever pressing for natural and artificial rewards. **a**, NR sampling trials from all mice were separated in two data sets depending on the latency to press (<10 s and >10 s) to determine whether the observed ramping of DA before pressing was only due to jitter effect of previous events (ie, cue and lever extension) or DA transients linked to approach behavior. Normalized dLight signal recorded during NR sampling trials from all expert mice were aligned to the lever extension (2 s after the cue onset) and to the press for both data sets (*n* = 114 mice). In trials with a delay to press higher than 10 s, the rise of dLight signal started before the press, suggesting an effect linked to the approach behavior. Note that ramping of dLight signal was also seen in trials without confounding earlier events. **b**, Same analysis for short-oDASS sampling trials from all expert mice (*n* = 68). **c**, Same analysis for long-oDASS sampling trials from all expert mice (*n* = 54). **d**, Same analysis for intravenous cocaine (1.25 mg/kg/infusion) sampling trials from all expert mice (*n* = 43). Data are represented as mean ± s.e.m.

As seen above with oDASS, the dLight signal, in expert mice, increased before the lever P in cocaine sampling trials. Such DA ramps could reflect transients evoked by prior events (cue and Le) averaged between several trials (jitter effect is due to variable delays between the Le and the P) or the lever approach behavior. To disentangle these possibilities, we separately analyzed the trials according to the latency between the Le and the lever P. In short-latency trials (<10 s), the DA ramp was in large part explained by a superimposition of the dLight signal with the one from previous events. In long-latency trials (≥10 s), a remaining DA ramp was observed for all reward types, suggesting a DA signal associated with the lever approach (Extended Data Fig. 7).

### The oDASS with punishment risk reduces associated cue-evoked DA transients favoring NR preference

**Fig. 5:**
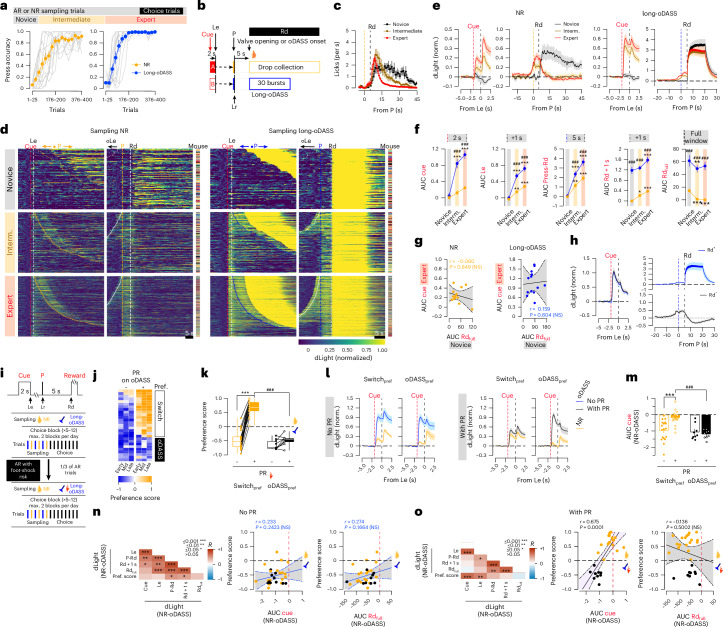
Punishment risk on oDASS attenuates cue-evoked DA transient and biase reward preference. **a**, Accuracy to P for NR or long-oDASS during the first 400 trials and accuracy to P (*n* = 13 mice). Three learning steps were defined based on P accuracy. **b**, Operant task schedule. Rewards are either a drop of fat solution or long-oDASS (30 bursts 500 ms apart of 5 pulses at 20 Hz). **c**, Number of licks per s around the NR delivery (*n* = 13 mice). **d**, Normalized dLight signal in hit sampling trials during the three learning periods (1,070 out of 2,259 sampling trials for the NR and 1,096 out of 1,951 for long-oDASS) sorted according to the P latency (*n* = 13 mice). DA transients were aligned either to the Le (white dashed line, 2 s after the cue onset) or to the lever P (orange or blue dashed line, 5 s before the Rd), from −10 s to +45 s or +30 s for, respectively, NR and long-oDASS. Missed trials are not shown. Orange (or blue in long-oDASS trials) dots indicate P and white dots Le. **e**, Grouped data of dLight signal aligned to the Le or the P for NR (left) and long-oDASS (right) sampling during the three learning periods. **f**, Mean AUC of normalized dLight during the three learning periods. Two-way RM ANOVA: for cue, main period effect *F*[2,24] = 73.95, *P* ≤ 0.001, *η*^2^_p_ = 0.860; reward-type effect *F*[1,12] = 37.08, *P* ≤ 0.001, *η*^2^_p_ = 0.371 and interaction period × reward type *F*[2,24] = 17.71, *P* ≤ 0.001, *η*^2^_p_ = 0.596; for Le, main period effect *F*[2,24] = 107.1, *P* ≤ 0.001, *η*^2^_p_ = 0.899, reward-type effect *F*[1,12] = 21.47, *P* ≤ 0.001, *η*^2^_p_ = 0.641 and interaction period × reward type *F*[2,24] = 17.37, *P* ≤ 0.001, *η*^2^_p_ = 0.591; during P-Rd, main period effect *F*[2,24] = 46.50, *P* ≤ 0.001, *η*^2^_p_ = 0.795, reward-type effect *F*[1,12] = 29.92, *P* ≤ 0.001, *η*^2^_p_ = 0.714 and interaction period × reward type *F*[2,24] = 6.59, *P* ≤ 0.01, *η*^2^_p_ = 0.354; for 1 s after Rd, main period effect *F*[2,24] = 15.44, *P* ≤ 0.001, *η*^2^_p_ = 0.563 and reward-type effect *F*[1,12] = 173.20, *P* ≤ 0.001, *η*^2^_p_ = 0.935; for Rd_full_, main period effect *F*[2,24] = 8.33, *P* ≤ 0.01, *η*^2^_p_ = 0.410 and reward-type effect *F*[1,12] = 67.68, *P* ≤ 0.001, *η*^2^_p_ = 0.849. ANOVAs are followed by Bonferroni’s test: ^*^P ≤ 0.05, ^**^P ≤ 0.01, ^***^P ≤ 0.001, for intermediate or expert versus novice; ^###^*P* ≤ 0.001 for NR versus long-oDASS (*n* = 13 mice). **g**, Correlation between AUC of normalized dLight at expert cue and maximum AUC at novice Rd_full_, for NR and long-oDASS (two-tailed Spearman’s: *r*_11_ = −0.060, *P* = 0.8491, 95% CI (0.604, 0.521) and *r*_11_ = 0.159, *P* = 0.6040, 95% CI (0.444, 0.663), respectively, *n* = 13 mice). **h**, The dLight signal recorded from expert mice (*n* = 12) during sampling trials with or without Rd (Rd^+^ or Rd^−^). Normalized dLight signal aligned to the Le or the P, with or without the Rd (long-oDASS). **i**, Schedule of experiment. Experiment contained two successive phases, without and with PR and consisted of alternating blocks of sampling and choice trials with NR and long-oDASS in expert mice (*n* = 27). **j**, Behavioral clustering based on individual preference score (*n* = 27 mice), P accuracy and latency in sampling and choice trials (only preference score is shown; Extended Data Fig. 8). **k**, Preference score distribution (two-way RM ANOVA, main cluster effect *F*[1,25] = 45.41, *P* ≤ 0.001, *η*^2^_p_ = 0.645, PR effect *F*[1,25] = 164.40, *P* ≤ 0.001, *η*^2^_p_ = 0.868 and interaction cluster × PR *F*[1,25] = 94.53, *P* ≤ 0.001, *η*^2^_p_ = 0.791, followed by Fisher’s LSD test: ^***^P ≤ 0.001 for a significant effect of PR; ^###^*P* ≤ 0.001 for between-cluster effect; *n* = 18 and 9 mice). Box plots represent the distribution (median represents the 25th and 75th percentiles and whiskers the 5th and 95th percentiles). **l**, Normalized dLight signal aligned to Le for two clusters, without and with PR. **m**, Quantification of the difference of the normalized dLight signal evoked at cue associated with long-oDASS and NR, without and with the PR on oDASS (two-way RM ANOVA, main cluster effect *F*[1,25] = 11.89, *P* = 0.002, *η*^2^_p_ = 0.322, PR effect *F*[1,25] = 7.45, *P* = 0.012, *η*^2^_p_ = 0.229 and interaction cluster × PR *F*[1,25] = 14.25, *P* ≤ 0.001, *η*^2^_p_ = 0.363, followed by Fisher’s LSD test: ^***^P ≤ 0.001 for a significant effect of PR; ^###^*P* ≤ 0.001 for between-cluster effect; *n* = 18 and 9 mice). **n**, Left: correlation table between the difference of AUC of normalized dLight (NR-oDASS) calculated for the different events of the trial and the preference score measured from later choice trials. Middle and right: correlation between the preference score and the difference of normalized dLight signal evoked at the specific cue or after Rd (two-tailed Spearman’s: *r*_25_ = 0.233, *P* = 0.2423, 95% CI (0.173, 0.571) and *r*_25_ = 0.274, *P* = 0.1664, 95% CI (−0.130, +0.600), respectively; *n* = 27 mice). **o**, Same as **n** for the second phase of the experiment, with PR on long-oDASS (two-tailed Spearman’s: *r*_25_ = 0.675, *P* ≤ 0.001, 95% CI (0.387, 0.843) and *r*_25_ = −0.136, *P* = 0.5002, 95% CI (−0.499, +0.269), respectively; *n* = 27 mice). Data are represented as the mean ± s.e.m. N, novice; I, intermediate; E, expert.

**Extended Data Fig. 8:**
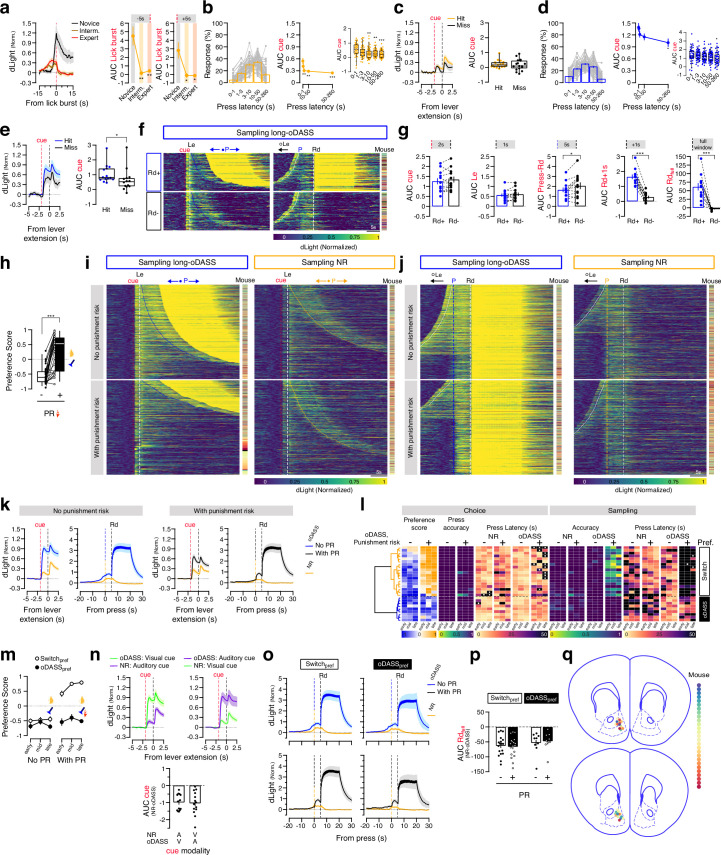
Behavioral clustering for long-oDASS *versus* NR preference and accumbal dLight during sampling trials with or without punishment. **a**, Left, normalized dLight signal aligned to the onset of the first lick burst after Rd during the three learning phases. Right, normalized dLight AUC before and after the lick burst onset in the three learning phases (one-way mixed ANOVA, respectively F[2,24] = 17.97, *P* = 0.0008, η^2^_p_ = 0.600 and F[2,24] = 9.84, *P* = 0.0063, η^2^_p_ = 0.451, followed by a Dunnett’s test: *P ≤ 0.05 or ** *P* ≤ 0.001 for intermediate and expert *versus* novice phase, *n* = 13 mice). **b**, Left, distribution of press latencies for NR sampling trials in expert mice (*n* = 54). Right, AUCs of dLight signal for 2 s after the cue onset recorded from expert mice as a function of the latency to press (one-way mixed ANOVA F[4,247] = 5.19, *P* = 0.0005, η^2^_p_ = 0.078, followed by Dunnett’s test: ** *P* ≤ 0.01 or ***P ≤ 0.001 when compared to 0-1 s press latency, *n* = 54 mice). **c**, Left, grouped data of dLight signal aligned to the lever extension (2 s after the cue onset) for hit and miss trials in expert mice. Right, AUCs of dLight signal for 2 s after the cue in hit and miss trials (Two-tailed paired *t*-test, t16 = 0.61, *P* = 0.5480, *n* = 17/54 mice, 70 miss and 3092 hit trials). **d**, Left, distribution of press latencies for long-oDASS trials in expert mice (*n* = 54 mice). Right, AUCs of dLight signal for 2 s after the cue onset as a function of the latency to press (one-way mixed ANOVA F[4,256] = 3.05, *P* = 0.0177, η^2^_p_ = 0.045, followed by Dunnett’s test: *P ≤ 0.05 when compared to 0-1 s press latency, *n* = 54 expert mice). **e**, Left, grouped data of normalized dLight signal aligned to the lever extension (2 s after the cue onset) for hit (blue) and miss (black) long-oDASS sampling trials. Only data from mice having missed trials are shown (*n* = 14/54 expert mice, 29 miss and 3137 hit trials from all study). Right, distribution of AUCs of normalized dLight signal in hit and miss trials of long-oDASS sampling (Two-tailed paired *t*-test, t13 = 2.86, *P = 0.0135, *n* = 14/54). **f**, Normalized dLight signal recorded during long-oDASS sampling trials with or without the reward delivery (Rd + , Rd-) Session consisted of 24 trials with 50% of randomly interleaved omission or delivery of the optogenetic stimulation. Recordings are aligned at the lever extension (white dashed line) or at the press (blue dashed line), data from –10 s to +30 s. Trials are sorted as a function of the delay to press. Blue dots are for Press and white dots are for Le (*n* = 12 expert mice). **g**, AUCs of the normalized dLight signal for each of the 5 windows (Two-tailed paired *t*-test, t11 = 1.20, *P* = 0.2552; t11 = 0.99, *P* = 0.3428; t11 = 2.56, *P = 0.0268; t11 = 10.70, ***P ≤ 0.001; t11 = 5.82, ***P ≤ 0.001, *n* = 12 mice). **h**, Preference score without or with punishment risk on long-oDASS (Two-tailed paired *t*-test, t26 = 7.95, ***P ≤ 0.001, for without *versus* with PR, *n* = 27 mice). **i**, Normalized dLight signal recorded with photometry during sampling trials for the NR or for long-oDASS, during the two phases. Recordings of each trial are aligned to the lever extension (-10 to +30 s) and sorted as a function of the delay to press. Missed trials are shown at the bottom. Orange and blue dots are for lever presses. **j**, Same as (i) but with trials aligned to the lever press (orange or blue dashed line, P, 5 s before the reward delivery). White dots indicate Le. **k**, Normalized dLight signal aligned to the lever extension (Le, 2 s after the cue onset) or to the press (5 s before Rd) during the sampling trials for long-oDASS and NR recorded from the expert mice (*n* = 27), without and with the punishment risk. **l**, Behavioral clustering based on individual preference score (*n* = 27 mice), press accuracy and latency in sampling and choice trials (white dots when only missed trials). **m**, Evolution of the preference score for the two clusters of mice (n = 18 and 9 mice). **n**, dLight data at cue, separated by modality of cue to long-oDASS and NR during sampling trials (Two-tailed unpaired *t*-test, t25 = 0.23, *P* = 0.8209, *n* = 14 and 13 mice, for auditory and visual modality of the cue associated with long-oDASS, respectively). Note that the modality was randomly assigned for each mouse. **o**, Normalized dLight signal aligned to press (5 s before the reward delivery) during the sampling trials for long-oDASS and NR, without or with punishment risk. **p**, Quantification of the difference of normalized dLight signal recorded after the reward delivery (AUC on the full-time window) in long-oDASS and NR sampling trials, without or with punishment risk (two-way RM ANOVA, no significant effects of clusters or risk of punishment were found, *n* = 18 and 9). **q**, Coronal sections (antero-posterior coordinates +1.54 and + 1.70 from bregma) containing the Nucleus Accumbens are shown. Dots are for the center of the dLight infection for each mouse. Box plots represent the distribution (median, 25 and 75%, whiskers are for 5 and 95%). Data are represented as mean ± s.e.m.

Next, we aimed to test the consequences of a punishment risk (mild foot-shock) applied on long-oDASS on both NAc DA transients and preference. We first established the learning of NR and long-oDASS (30 bursts of 5 pulses at 20 Hz, 500 ms apart) sampling following the approach described above (Fig. 5a–f and Extended Data Fig. 8a). Overall, mice learned to lever P for both NR and long-oDASS and DA transients emerged at cue, becoming much higher (5.4× AUC) for long-oDASS than for NR (Fig. 5e,f). As previously, no correlation was found between an individual’s DA transient at expert cues and DA transient at novice Rd (Fig. 5g). P latency or missed trials weakly matched the amplitude of DA transients evoked at associated cues, confirming a modest trial-by-trial motivation role (70 miss and 3,092 hit for NR; 29 miss and 3,137 hit for long-oDASS sampling trials; Extended Data Fig. 8b–e). The omission of long-oDASS in expert mice produced a deflection of the DA signal starting at the time of the expected Rd (5 s after the lever P). The DA transient evoked by the discriminative cue remained unchanged between rewarded and unrewarded trials (Fig. 5h and Extended Data Fig. 8f,g).

Expert mice were then subjected to the two-reward choice procedure. The initial experimental condition (no punishment risk (PR)) was followed by a devaluation-like situation (oDASS under a PR) (Fig. 5i). The preference score was significantly reversed by the PR, toward more NR choices (Extended Data Fig. 8h). Accordingly, in the absence of PR, DA transients recorded in interleaved sampling trials were much higher (5.4× AUC) at cue associated with long-oDASS than with NR. This difference in DA transient between the two cues was reduced by the PR (2.6× AUC). DA transients after the Rd remained unchanged by the PR and were always much higher for long-oDASS than for NR (Extended Data Fig. 8i–k). Hierarchical clustering based on behavior revealed two distinct groups of mice reflecting the different trajectories: mice that switched their preference from long-oDASS to NR under PR (Switch_pref._, 66.7%) and mice that confirmed their long-oDASS preference despite the PR (oDASS_pref._, 33.3%) (Fig. 5j,k and Extended Data Fig. 8l,m). DA transients at the long-oDASS-associated cue were much stronger than at the NR-associated cue during the phase without PR for both clusters, in line with all mice preferring long-oDASS. This difference tended to attenuate for the mice switching their preference while staying high in the mice still choosing long-oDASS despite the risk of foot-shock (Fig. 5l,m). Importantly, cue modality (visual and auditory), randomly attributed to long-oDASS and NR for each mouse, had no influence on DA value coding at cue or on preference (Extended Data Fig. 8n). DA transients after NR delivery and after oDASS did not differ between the two clusters of mice and did not evolve with introduction of the PR (Extended Data Fig. 8o,p). The dLight was expressed in the NAc of all mice (Extended Data Fig. 8q). With PR, we found a highly significant correlation between the DA transients at cues during sampling trials and the preference score, but not for DA transients at proximal events or after the Rd. No correlation was found in the absence of PR because all mice preferred long-oDASS (Fig. 5n,o). Taken together, these results highlight that the difference in DA transients evoked by the discriminative cue can adapt with the PR on AR together with the preference, but that some animals escaped this process.

### AR preference predicted perseverance to punished oDASS

**Fig. 6:**
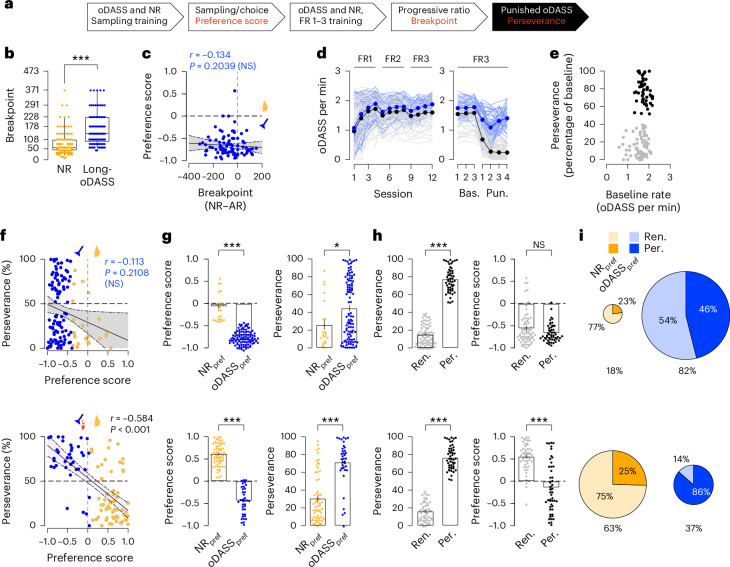
Preference predicts perseverance to punished oDASS. **a**, Timeline of the experiments. **b**, Left: breakpoint for NR and long-oDASS (two-tailed, paired Student’s *t*-test, *t*90 = 6.33, ^***^P ≤ 0.001, for long-oDASS versus NR; *n* = 91 mice). Box plots represent the distribution (median represents the 25th and 75th percentiles and whiskers the 5th and 95th percentiles). **c**, Correlation between the preference score and the breakpoint difference (BP_NR_ − BP_AR_) (two-tailed Spearman’s: *r*_89_ = −0.134, *P* = 0.2039, 95% CI (−0.337, +0.080), *n* = 91 mice). **d**, long-oDASS rate during 60-min sessions with increasing fixed ratio (left) and long-oDASS rate during 30-min session without and with the risk of foot-shock (0.25 mA, 500 ms) every third stimulation (left, *n* = 124 mice). **e**, long-oDASS perseverance (percentage rate of laser stimulation during punished sessions 3–4 to baseline rate) as a function of baseline rate (*n* = 124). The color code indicated classification as renouncers (gray) and perseverers (black). **f**, Correlation between the perseverance for long-oDASS and the preference score for NR versus long-oDASS (top, two-tailed Spearman’s: *r*_122_ = −0.113, *P* = 0.2108, 95% CI (−0.289, +0.070), *n* = 124 mice) or NR versus long-oDASS with PR (bottom). Top: two-tailed Spearman’s: *r*_98_ = −0.584, *P* ≤ 0.001, 95% CI (−0.703, −0.433), *n* = 100 mice). **g**, Preference score and perseverance for data grouped as a function of the choice clustering. Top: two-tailed, unpaired Student’s *t*-test, *t*122 = 15.36, ^***^P ≤ 0.001 and *t*122 = 2.41, *P* = 0.0173, respectively, for NR_pref._ versus long-oDASS_pref_ (*n* = 102 and 22). Bottom: two-tailed, unpaired Student’s *t*-test, *t*98 = 18.97, ^***^P ≤ 0.001 and *t*98 = 7.37, *P* ≤ 0.001, respectively, for NR_pref._ versus long-oDASS_pref_ (*n* = 62 and 37 mice). **h**, Perseverance and preference score for data grouped as a function of the perseverance clustering. Top: two-tailed, unpaired Student’s *t*-test, *t*122 = 27.35, ^***^P ≤ 0.001 and *t*122 = 1.89, *P* = 0.001, respectively, for Ren. versus Per. (*n* = 72 and 52). Bottom: two-tailed, unpaired Student’s *t*-test, *t*98 = 24.85, ^***^P ≤ 0.001 and *t*98 = 7.78, *P* ≤ 0.001, respectively, for renouncers (Ren.) versus perseverers (Per.) (*n* = 52 and 48 mice). **i**, Charts for intersection between choice and perseverance clusters. The size of the pie charts is relative to preference proportions (top, *n* = 124 and, bottom, *n* = 100 mice). Data are represented as the mean ± s.e.m. Bas., baseline; Pun., punishment.

To evaluate the place of an individual AR preference assessed in choice paradigm in the processus of addiction, we determined the breakpoint for each reward (long-oDASS and NR) in a progressive ratio schedule session and further evaluated individual addiction-related behavior with the perseverance for long-oDASS despite PR (Fig. 6a). We found no correlation between breakpoint difference (NR − AR) and the preference score, most likely because of the strong long-oDASS preference and the weak interindividual variability (Fig. 6b,c). In the oDASS perseverance task, previously used to determine compulsivity^35,36^, mice maintained their stimulation rate when the fixed ratio was increased (Fig. 6d) and about half the mice continued pressing the lever for long-oDASS when a PR (foot-shock of 0.25 mA, 500 ms) was introduced, whereas the other half had strongly reduced responding, in line with previous studies^32^. As previously described^32,36^, the mice were classified as renouncing or persevering, based on the perseverance score, which was uncorrelated with the oDASS rate at baseline (Fig. 6e). The oDASS perseverance did not significantly correlate with preference, but, among mice with NR or weak oDASS preference, a large majority classified as renouncers. Within mice with oDASS preference, about half were then found to be perseverers (Fig. 6f–i).

We therefore looked at the preference between NR and oDASS with PR and found that individual perseverance strongly correlated with preference score. Of mice preferring oDASS despite PR over the NR, 86% then classified as perseverers. It is interesting that, among persevering mice, 10–15% chose the NR rather than the oDASS with PR, suggesting that the alternative reward option could oppose the transition to addiction-related behavior. This hints at a relationship where individual preference (and hence DA value coding) may predict the transition to compulsion (Fig. 6f–i). This suggested that initial preference represents a necessary step but is not sufficient for transition to addiction.

In summary, the trajectory of an individual mouse toward addiction may be inferred from its initial AR preference and eventually by DA value encoded at predictive associated cues.

### Preference for weak ARs reveals vulnerability to addiction-like behavior

**Fig. 7:**
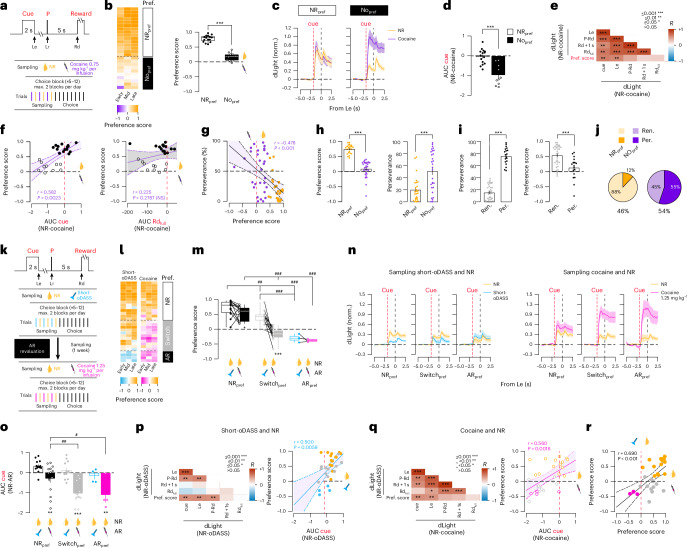
Preference for weak artificial rewards reveals vulnerability to addiction-like behavior. **a**, Schedule of experiments. Photometry recordings were done during sampling trials and evaluation of preference between the NR and intravenous cocaine (0.75 mg kg^−1^ per infusion) was done in choice trials (*n* = 25 mice). **b**, Preference score distribution for the two behavioral clusters (two-tailed, unpaired Student’s *t*-test, *t*23 = 12.17, ^***^P ≤ 0.001, *n* = 15 and 10 for NR versus no preference; Extended data Fig. 9). **c**, Normalized dLight signal aligned to the Le (2 s after cue onset) during the sampling trials for cocaine and NR (*n* = 25 mice). **d**, Quantification of the difference of the normalized dLight signal evoked at cue associated with NR and cocaine (two-tailed, unpaired Student’s *t*-test, *t*23 = 5.24, ^***^P ≤ 0.001, *n* = 15 and 10 for NR versus No_pref._). **e**, Table of correlation between the difference of AUC of normalized dLight (NR-cocaine) calculated for the different events of the sampling trial and the preference score measured from later choice trials. **f**, Correlation between the preference score and the difference of normalized dLight signal evoked at the specific cue or after Rd (two-tailed Spearman’s: *r*_23_ = 0.582, *P* = 0.0023, 95% CI (0.230, 0.799) and *r*_23_ = 0.225, *P* = 0.2787, 95% CI (−0.199, +0.578), respectively; *n* = 25 mice). **g**, Correlation between the preference score for NR versus cocaine 0.75 mg kg^−1^ per infusion and the perseverance for long-oDASS (two-tailed Spearman’s: *r*_52_ = −0.478, *P* = 0.0003, 95% CI (−0.666, −0.233); *n* = 54 mice). **h**, Preference score and perseverance for data grouped as a function of the choice clustering (two-tailed, unpaired Student’s *t*-test, *t*52 = 10.85, ^***^P ≤ 0.001 and *t*52 = 3.99 ^***^P = 0.0002, for, respectively, preference score and perseverance, for NR_pref._ versus No_pref._, *n* = 25 and 29 mice). **i**, Perseverance and preference score for data grouped as a function of the perseverance clustering (two-tailed, unpaired Student’s *t*-test, *t*52 = 16.39, ^***^P ≤ 0.001 and *t*52 = 4.18, ^***^P = 0.0001, for, respectively, perseverance and preference index, for Ren. versus Per.; *n* = 35 and 19 mice). **j**, Charts for intersection between choice and perseverance clusters. The size of the pie charts is relative to preference proportions. **k**, Schedule of experiment. The experiment consisted of two successive phases, beginning with short-oDASS and then cocaine 1.25 mg kg^−1^ per infusion (*n* = 29 mice). Between the two phases, mice were trained with cocaine for 1 week. **l**, Evolution of individual preference score and behavioral clusters of mice (*n* = 14, 11 and 4 mice). Behavioral clustering is based on the individual preference score (*n* = 27 mice), P accuracy and latency in sampling and choice trials (only preference score is shown; Extended Data Fig. 9). **m**, Preference score distribution (two-way RM ANOVA, main cluster effect *F*(2,26) = 60.25, *P* ≤ 0.001, *η*^2^_p_ = 0.823; AR effect *F*[1,26] = 17.37, *P* = 0.0003, *η*^2^_p_ = 0.401 and interaction cluster × AR type *F*[2,26] = 6.78, *P* = 0.0043, *η*^2^_p_ = 0.343, followed by Bonferroni’s test: ^*^P ≤ 0.05, ^***^P ≤ 0.001 for short-DASS versus cocaine; ^##^*P* ≤ 0.01, ^###^*P* ≤ 0.001 for effects between clusters; *n* = 14, 11 and 4 mice). **n**, Normalized dLight signal aligned to the Le for each cluster, with short-oDASS and NR or cocaine and NR. **o**, Quantification of the difference of the normalized dLight signal evoked at cue associated with AR and NR, with short-oDASS or cocaine (two-way RM ANOVA, main cluster effect *F*[2,26] = 5.44, *P* = 0.0106, *η*^2^_p_ = 0.295; AR effect *F*[1,26] = 42.55, *P* ≤ 0.001, *η*^2^_p_ = 0.621, followed by Bonferroni’s test: ^**^P ≤ 0.01, ^***^P ≤ 0.001 for short-oDASS versus cocaine; ^#^*P* ≤ 0.05, ^##^*P* ≤ 0.01 for effects between clusters; *n* = 14, 11 and 4 mice). **p**, Left: table of correlation between the difference of AUC of normalized dLight (NR–AR) calculated for the different events of the trial and the preference score measured from later choice trials. Right: correlation between the preference score and the difference (NR–AR) of normalized dLight signal evoked at the cue (two-tailed Spearman’s: *r*_27_ = 0.500, *P* = 0.0059, 95% CI (0.151, 0.737); *n* = 29 mice). **q**, Same as **p** for the second phase of the experiment with cocaine 1.25 mg kg^−1^ per infusion as the AR (two-tailed Spearman’s: *r*_27_ = 0.560, *P* = 0.0016, 95% CI (0.233, 0.773); *n* = 29 mice). **r**, Correlation between preference score observed with the two successive ARs, short-oDASS and cocaine 1.25 mg kg^−1^ per infusion, respectively (two-tailed Spearman’s: *r*_27_ = 0.690, *P* ≤ 0.001, 95% CI (0.423, 0.846); *n* = 29 mice). Box plots represent the distribution (median represents the 25th and 75th percentiles and whiskers the 5th and 95th percentiles). Data are represented as the mean ± s.e.m.

**Extended Data Fig. 9:**
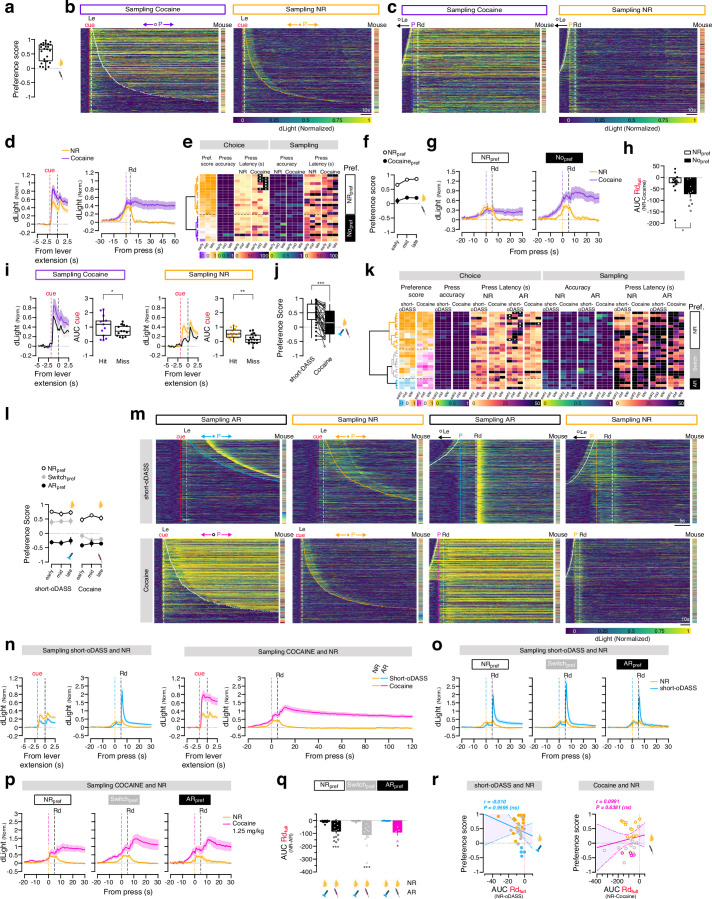
Behavioral clustering and accumbal dLight signals supporting cocaine *versus* natural reward preference and cocaine-driven revaluation of short-oDASS. **a**, Preference score distribution for cocaine (0.75 mg/kg/infusion) *versus* NR choice (*n* = 25 mice). **b**, Normalized dLight signal recorded with photometry during sampling trials for the NR or for cocaine. Recordings of each trial are aligned to the lever extension (-10 to +120 s) and sorted as a function of the delay to press. Missed trials are shown at the bottom. White and orange dots are for lever presses. **c**, Same as (b) but with trials are aligned to the lever press (purple or orange dashed line, P, 5 s before the reward delivery, Rd). White dots indicate Le. **d**, Normalized dLight signal aligned to the lever extension (le, 2 s after the cue onset) or to the press (5 s before Rd) during the sampling trials for cocaine and NR (*n* = 25 mice). **e**, Behavioral clustering based on individual preference score (*n* = 25 mice), press accuracy and latency in sampling and choice trials (white dots when only missed trials). **f**, Evolution of the preference score for the two clusters of mice (n = 15 and 10 mice). **g**, Normalized dLight signal aligned to press (5 s before the reward delivery) during the sampling trials for cocaine and NR (n = 15 and 10 mice, for NR preference and No preference, respectively). **h**, Quantification of the difference of normalized dLight signal recorded after the reward delivery (AUC on the full-time window) in cocaine and NR sampling trials (Two-tailed paired *t*-test, t23 = 2.38, *P* = 0.026, *n* = 15 and 10). **i**, Left for cocaine sampling trials, grouped data of dLight signal aligned to the lever extension (2 s after the cue onset) for hit and miss trials in expert mice. AUCs of dLight signal for 2 s after the cue in hit and miss trials (Two-tailed paired *t*-test, t12 = 2.30, *P = 0.0402, *n* = 13/25 mice having miss trials, 57 miss and 633 hit trials). Right for NR sampling trials, grouped data of dLight signal aligned to the lever extension (2 s after the cue onset) for hit and miss trials in expert mice. AUCs of dLight signal for 2 s after the cue in hit and miss trials (Two-tailed paired *t*-test, t15 = 3.15, **P = 0.0065, *n* = 16/25 mice having miss trials, 80 miss and 1034 hit trials). **j**, Preference score for choice between NR and short-oDASS and then between NR and cocaine (1.25 mg/kg/infusion) (Two-tailed paired *t*-test, t28 = 4.60, ***P ≤ 0.001, for cocaine *versus* short-oDASS, *n* = 29 mice). **k**, Behavioral clustering based on individual preference score (*n* = 29 mice), press accuracy and latency in sampling and choice trials (white dots when only missed trials). **l**, Evolution of the preference score for the three clusters of mice (n = 14, 11 and 4 mice). **m**, Left, normalized dLight signal recorded with photometry during sampling trials for the NR or AR, during the two phases (short-oDASS and then cocaine). Recordings of each trial are aligned to the lever extension (-10 to +30 s for the first phase and -10 to +120 s for the second phase) and sorted as a function of the delay to press. Missed trials are shown at the bottom. Orange, white and blue dots are for lever presses. Right, for trials aligned to the lever press (orange, pink or blue dashed line, P, 5 s before the reward delivery). White dots indicate Le. **n**, Normalized dLight signal aligned to Le (2 s after the cue onset) or to the press (5 s before Rd) during the sampling trials for short-oDASS and NR, and then for cocaine and NR (*n* = 29 mice). **o**, Normalized dLight signal by clusters, aligned to press (5 s before the reward delivery) during the sampling trials for short-oDASS and NR. **p**, Normalized dLight signal by clusters, aligned to press (5 s before the reward delivery) during the sampling trials for cocaine and NR. **q**, Quantification of the difference of normalized dLight signal recorded after the reward delivery (AUC on the full-time window) in short-oDASS and NR sampling trials, and then in cocaine and NR sampling trials (two-way RM ANOVA, main cluster effect F[2,26] = 0.42, *P* = 0.6595, η^2^_p_ = 0.032; main AR effect F[1,26] = 41.48, *P* ≤ 0.001, η^2^_p_ = 0.615; interaction cluster x AR type F[2,26] = 0.62, *P* = 0.5438, η^2^_p_ = 0.046, followed by a Bonferroni test: *P ≤ 0.05, ***P ≤ 0.001 for short-oDASS *versus* cocaine, *n* = 14, 11 and 4 mice). **r**, Correlation between the preference score and the AUC difference (NR-AR) of normalized dLight signal evoked after Rd, when AR is short-oDASS and then cocaine (Two-tailed Spearman’s: r_27_ = -0.010, *P* = 0.9595, 95% CI [-0.385,0.368] and r_27_ = 0.091, *P* = 0.6381, 95% CI [-0.295,0.452], respectively, *n* = 29 mice). Box plots represent the distribution (median, 25 and 75%, whiskers are for 5 and 95%). Data are represented as mean ± s.e.m.

To further test whether an initial preference for ARs forecasts addiction vulnerability, we correlated the preference for a low dose of cocaine (0.75 mg kg^−1^) (Fig. 7a and Extended Data Fig. 9a) with the subsequent oDASS perseverance. We also checked whether individuals with a preference for short-oDASS would re-evaluate this when offered cocaine at a higher dose (1.25 mg kg^−1^) (Fig. 7k).

After the sampling trials, DA transients at NR-associated and cocaine-associated cues were on average for the population of similar amplitude (0.6× AUC; Extended Data Fig. 9b–d). We identified two behavioral clusters: mice that preferred the NR (NR_pref._, 60%) and mice that chose NR and cocaine equally (No_pref._, 40%) (Fig. 7b and Extended Data Fig. 9e,f). Again, cue-associated DA transients correlated best with the individual preference. Miss and hit trials were 57 and 633 for cocaine and 80 and 1,034 for NR sampling trials (Fig. 7c–f and Extended Data Fig. 9g–i). The mice were next subjected to the oDASS perseverance task, which yielded a strong correlation with preference score. Importantly, mice with a weak AR preference were then found to persevere for oDASS under punishment risk (Fig. 7g–j).

In a separate experiment, we observed that mice with an initial preference for a weak AR (short-oDASS) were more susceptible to next develop a strong preference for cocaine, used at high dose. As expected, an individual variability of preference between short-oDASS and NR that correlated with DA transient at cues was found. At the population level, DA transient at NR-associated cue was bigger than that at short-oDASS-associated cue (2× AUC; Extended Data Fig. 9n). Behavioral trajectories were defined with the three clusters: 48.3% mice kept choosing preferentially the NR when cocaine replaced short-oDASS (NR_pref._), 37.9% mice shifted their preference toward cocaine (Switch_pref._) and 13.8% mice preferred short-oDASS and then cocaine (AR_pref._) (Fig. 7l,m). Remarkably, replacing short-oDASS by an intravenous infusion of a high dose of cocaine (1.25 mg kg^−1^ per infusion) triggered revaluation not only of the preference, but also of the value encoded by the associated cues (Fig. 7n,q and Extended Data Fig. 9j–r). At the population level, DA transient at cue now associated with cocaine became much bigger than that at NR-associated cue (2.7× AUC; Extended Data Fig. 9n). This experiment further demonstrated that individual preference for weak AR forecasts addiction-related behavior, as shown by the correlation between preferences (Fig. 7r).

## Discussion

We observed pronounced interindividual variability when mice chose between an AR (cocaine or oDASS) and an NR (fat solution). The individual preference was best predicted by the difference in cue-evoked DA transients in the NAc, supporting the view that these signals encode subjective reward value^9,19^ rather than objective reward magnitude. Preference for weak ARs over NR emerged as a risk factor for addiction-like behaviors.

We used an operant task initiated by discriminative cues to prevent value generalization. In a recent study^21^, DA neuron activity peaked at a nondiscriminative trial-start cue regardless of reward identity. In the absence of discriminative cues, value generalization can extend to reward-associated contexts^39,41^. In our task, incidental stimuli occurring between cue onset and Rd evoked DA transients that correlated weakly with preference, whereas Rd likely functioned as a proximal cue. Before lever pressing, DA increased progressively for all reward types, consistent with a role for DA in approach behavior^16^.

Trial-by-trial analyses revealed only a weak correlation between cue-evoked DA amplitude and response latency, indicating limited motivational contribution. This aligns with evidence that replacing a cue with optogenetic stimulation of VTA DA neurons fails to trigger reward seeking^42^. Nevertheless, cue-evoked DA transients were often smaller in trials in which animals failed to press the lever, particularly for preferred rewards. These findings indicate that, although cue-evoked DA encodes value, additional contextual signals guide trial-specific responding. DA at cues can acquire motivational influence when contextual information signals immediate reward availability^43^. To minimize motivational confounds, we did not food restrict mice. Breakpoints, which reflect reinforcer efficacy^44–46^, did not correlate with preference scores. Preference was likely also shaped by differences in effort and reward delay across reward types: obtaining NR required locomotion and cocaine delivery involved pharmacokinetic delays^47^, whereas optogenetic stimulation produced immediate DA increases.

To avoid confounds of inherent salience or novelty^48^, we selected auditory and visual cues that did not elicit DA transients in naIve mice. In expert mice, long-oDASS produced larger DA transients than short-oDASS, both during stimulation and at the predictive cue, consistent with theories proposing that DA encodes reward magnitude or objective value^10,19^. In contrast to NR, DA evoked by AR delivery itself remained stable despite the emergence of cue-evoked DA. Omitting optogenetic reward produced a DA dip that scaled with reward strength, consistent with RPE signaling.

A central result of this study is that the difference between cue-evoked DA transients associated with NRs and ARs robustly predicted individual preference. Although AR-evoked DA signals were stable across mice, the values encoded at cues varied widely. During learning, DA signals typically shift from Rd to the most distal predictive cue^49–52^. However, DA transients after early NR delivery did not predict later preference and, in some individuals, subjective valuation overrode population-level reward ranking. Cue-evoked DA integrates environmental context and cost–benefit information, thereby encoding subjective value^9,10,53,54^.

**Extended Data Fig. 10:**
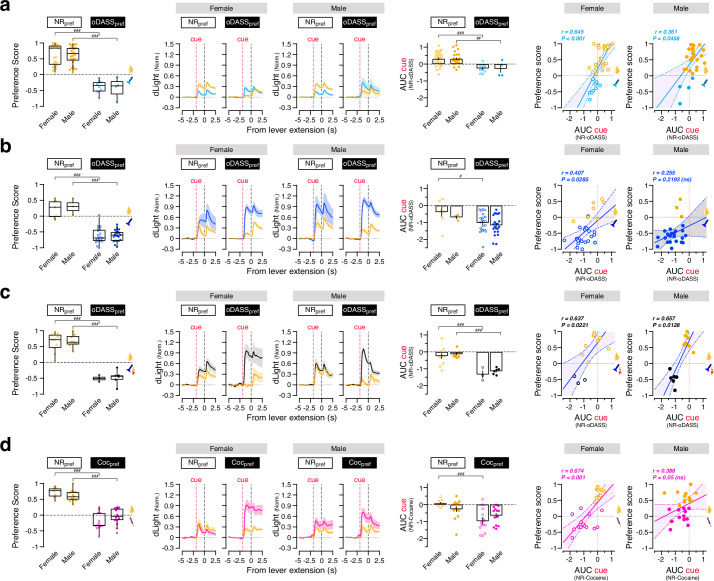
AR *versus* NR preference in female and male mice. **a**, Preference score as a function of behavioral clusters in female and male, for short-oDASS *versus* NR experiment (two-way ANOVA, main cluster effect F[1,64] = 108.5, *P* ≤ 0.001, η^2^_p_ = 0.629; main sex effect F[1,64] = 0.05, *P* = 0.8316, η^2^_p_ = 0.001, interaction F[1,64] = 0.03, *P* = 0.8573, η^2^_p_ = 0.001, followed by a Fisher LSD test: ###*p* ≤ 0.001 for groups with NR *versus* oDASS preference, *n* = 37 females and 31 males). Average normalized dLight traces for AR and NR aligned the lever extension (2 s after cue onset) during sampling trials in female and male of the two clusters (short-oDASS, *n* = 68). Corresponding AUC (NR-AR) of normalized dLight signal (two-way ANOVA, main cluster effect F[1,64] = 23.51, *P* ≤ 0.001, η^2^_p_ = 0.269; main sex effect F[1,64] = 0.01, *P* = 0.9356, η^2^_p_ = 0.0001; interaction F[1,64] = 0.01, *P* = 0.9159, η^2^_p_ = 0.0002, followed by a Fisher LSD test: ##*P* ≤ 0.01, ###*P* ≤ 0.001 for group with NR *versus* oDASS preference, *n* = 37 females and 31 males). Correlation between the preference score and the difference of normalized dLight signal AUCs at the cues (Two-tailed Spearman’s: r_35_ = 0.645, *P* ≤ 0.001, 95% CI [0.397,0.805] and r_29_ = 0.361, *P* = 0.0458, 95% CI [0.003,0.641], for female and male respectively, *n* = 37 and 31 mice). **b**, Same panels as (A) but with long-oDASS as the AR (*n* = 54). Preference score (two-way ANOVA, main cluster effect F[1,50] = 105.60, *P* ≤ 0.001, η^2^_p_ = 0.679; main sex effect F[1,50] = 0.17, *P* = 0.6831, η^2^_p_ = 0.003; interaction F[1,50] = 0.14, *P* = 0.7131, η^2^_p_ = 0.003, followed by a Fisher LSD test: ### *P* ≤ 0.001 for NR *versus* oDASS preference, *n* = 29 females and 25 males). Corresponding AUC (NR-AR) of normalized dLight signal (two-way ANOVA, main cluster effect F[1,50] = 6.58, *P* = 0.0134, η^2^_p_ = 0.116; main sex effect F[1,50] = 1.12, *P* = 0.2955, η^2^_p_ = 0.022; interaction F[1,50] = 0.15, *P* = 0.6973, η^2^_p_ = 0.003, followed by a Fisher LSD test: #*P* ≤ 0.05 for NR *versus* oDASS preference, *n* = 29 females and 25 males). Correlation between the preference score and the difference of normalized dLight signal AUCc at the cues (Two-tailed Spearman’s: r_27_ = 0.407, *P* = 0.0285, 95% CI [0.036,0.6799] and r_23_ = 0.255, *P* = 0.2193, 95% CI [-0.168,0.594], for female and male respectively, *n* = 29 and 25 mice). **c**, Same panels as (A) but with long-oDASS with punishment risk as the AR (*n* = 27). Preference score (two-way ANOVA, main cluster effect F[1,23] = 121.40, *P* ≤ 0.001, η^2^_p_ = 0.841; main sex effect F[1,23] = 0.16, *P* = 0.6947, η^2^_p_ = 0.007; interaction F[1,23] = 0.002, *P* = 0.9649, η^2^_p_ = 0.0001, followed by a Fisher LSD test: ###*P* ≤ 0.001 for group with NR *versus* oDASS preference, *n* = 13 females and 14 males). Corresponding AUC (NR-AR) of normalized dLight signal (two-way ANOVA, main cluster effect F[1,23] = 39.59, *P* ≤ 0.001, η^2^_p_ = 0.633; main sex effect F[1,23] = 0.85, *P* = 0.3654, η^2^_p_ = 0.036; interaction F[1,23] = 0.06, *P* = 0.8092, η^2^_p_ = 0.003, followed by a Fisher LSD test: ###*p* ≤ 0.001 for NR *versus* oDASS preference, *n* = 13 females and 14 males). Correlation between the preference score and the difference of normalized dLight signal AUCc at the cues (Two-tailed Spearman’s: r_11_ = 0.637, *P* = 0.0221, 95% CI [0.115,0.884] and r_12_ = 0.657, *P* = 0.0128, 95% CI [0.177,0.885], for female and male respectively, *n* = 13 and 14 mice). **d**, Same panels as (A) but with Cocaine 1.25 mg/kg/infusion as the AR (*n* = 49 mice). Preference score (two-way ANOVA, main cluster effect F[1,45] = 160.1, *P* ≤ 0.001, η^2^_p_ = 0.781; main sex effect F[1,45] = 0.54, *P* = 0.4640, η^2^_p_ = 0.012; interaction F[1,45] = 6.45, *P* = 0.0146, η^2^_p_ = 0.125, followed by a Fisher LSD test: ###*P* ≤ 0.001 for group with NR *versus* cocaine preference; *P ≤ 0.05 for male *versus* female, *n* = 23 females and 26 males). Corresponding AUC (NR-AR) of normalized dLight signal (two-way ANOVA, main cluster effect F[1,45] = 20.53, *P* ≤ 0.001, η^2^_p_ = 0.313; main sex effect F[1,45] = 0.003, *P* = 0.9533, η^2^_p_ = 0.0001; interaction F[1,45] = 4.73, *P* = 0.0349, η^2^_p_ = 0.0006, followed by a Fisher LSD test: ###*P* ≤ 0.001 for group with NR *versus* cocaine preference, *n* = 23 females and 26 males). Correlation between the preference score and the difference of normalized dLight signal AUCs at the cues (Two-tailed Spearman’s: r_21_ = 0.674, *P* ≤ 0.001, 95% CI [0.352,0.854] and r_24_ = 0.388, *P* = 0.05, 95% CI [-0.11,0.681], for female and male respectively, *n* = 23 and 26 mice). Box plots represent distribution of the preference score (median, 25 and 75%, whiskers are for 5 and 95%). Data are represented as mean ± s.e.m.

This subjective valuation likely underlies interindividual variability in AR versus NR preference and cannot be explained by genetics or sex. We used mice backcrossed for several generations on a quasi-clonal C57BL/6 background and included both sexes in similar proportions. We observed only weak sex-specific differences in preference and no significant sex differences in dLight signals, with females showing a slight bias toward ARs (Extended Data Fig. 10), as previously shown for drugs^55^.

Introduction of a PR reduced DA transients evoked by cues predicting a large optogenetic AR and reversed preference in a subset of mice. Cue-evoked DA never fell below NR-associated levels, indicating residual value that may contribute to relapse^22^. In contrast, devaluating a food reward by altering the internal state linearly reduced cue-evoked DA and shifted preference toward an alternative food reward^56^. Increasing AR strength, either by extending the duration of optogenetic stimulation or by substituting it by a high dose of cocaine, enhanced cue-evoked DA and shifted preference away from NR. By contrast, extended training with an AR that consistently produced strong DA transients at Rd (short-oDASS) neither enhanced DA at cue nor altered preference. Thus, cue-evoked DA encoding stabilizes after learning yet remains flexible under revaluation or punishment and deficits in updating these value representations may contribute to vulnerability to addiction.

Pairing optogenetic DA neuron stimulation with Rd during compound cue presentation generated an artificial positive RPE that enabled learning about an otherwise blocked cue^57^, an effect not reproduced by cocaine self-administration^58^. Although both cocaine and oDASS qualify as ARs, they differ mechanistically. Consistent with modeling-based theories^26,29^, cues predicting intravenous cocaine elicited stronger DA transients than NR-associated cues in most animals, especially with a high dose. However, despite higher cue-evoked DA, some mice continued to prefer NRs, indicating that choice behavior integrates additional factor NRs^26,28,29^.

We measured accumbal DA using the genetically encoded sensor dLight1.2, which provides a wide dynamic range and fast off-kinetics, avoiding saturation of DA peaks^59,60^. DA peaked before cocaine infusion onset due to predictive cues and remained elevated for minutes after the infusion. During acquisition, DA accumulated across closely spaced infusions, likely inflating cue value. During choice trials, longer ITIs may explain the weaker correlation between DA and preference. Alternatively, competing neuromodulatory systems, such as serotonin, may influence choice, consistent with its opponent role in cocaine perseverance^61^.

Preference for weak ARs, short oDASS or low-dose cocaine predicted the emergence of addiction-like behaviors. Only a subset of animals transitions to compulsive drug use^62,63^, at proportions similar to humans^64^. Preference for ARs forecast the transition to compulsion, revealed by perseverance in the oDASS despite punishment task. In mice that chose ARs despite punishment, cue-evoked DA differences between ARs and NRs remained stable, suggesting reduced value updating. Perseverance despite harm and choosing drugs over NRs appear to be tightly linked^65^. Individual preference for weak optogenetic ARs also correlated with preference for high-dose cocaine, indicating that DA-driven preference represents an early step in the addiction trajectory.

Individual variability in AR versus NR preference is therefore reflected from DA value assignment rather than the physical reward magnitude. As we used quasi-clonal mice, these differences likely reflect epigenetic mechanisms. Although phasic DA transients in the ventral striatum guide early choice, later addiction stages likely involve enduring synaptic plasticity and a shift of DA signaling toward the dorsal striatum^66–69^. Rapid DA-induced intracellular signaling triggers long-lasting gene expression and synaptic changes^70,71^ that preferentially emerge in vulnerable individuals and causally drive addiction-like behaviors^72,73^. By dissecting DA signaling related to choice, this study defines early mechanisms of addiction vulnerability and provides a framework for targeted prevention and intervention strategies.

## Methods

### Animals

Mice (aged 8–24 weeks) were heterozygous transgenic mice in which the Cre-recombinase expression was under the control of the regulatory elements of the DA transporter gene (DAT B6.SJL-Slc6a3tm1.1(cer)Bkmn/J, also known as DAT-IRES-Cre). Both female and male mice were used. Weight and sex were distributed homogeneously among the groups. Transgenic mice had been backcrossed into the C57BL/6 line for a minimum of four generations. The mice were single housed after surgery. All animals were kept in a temperature-controlled and humidity-controlled environment with an inverted 12-h light to 12-h dark cycle (light off at 07:00). All procedures were approved by the Institutional Animal Care and Use Committee of the University of Geneva and the Animal Welfare Committee of the Cantonal of Geneva, in accordance with Swiss law. All experiments were performed during the dark period of the inverted light-to-dark cycle.

### Surgery

#### Jugular vein catheter

The surgical procedure was performed as previously^74^. Briefly, mice were anesthetized with a mix of ketamine (60 mg per kg of Ketalar) and xylazine (12 mg per kg of Rompun). Catheters (SAI Infusion Technologies, cat. no. MSB-05B) made of silicone elastomer tubing (outside diameter 0.63 mm, inside diameter 0.30 mm) were inserted 1.0–1.2 cm into the right jugular vein, about 5 mm from the pectoral muscle, to reach the right atrium. The other extremity of the catheter was placed subcutaneously in the midscapular region and connected to stainless steel tubing. Successful placement was confirmed by withdrawing blood from the vein. Mice were allowed to recover for 3–5 d before the start of drug self-administration training and received paracetamol in drinking water (2 mg ml^−1^, orally) and the antibiotic, amikacin (10 mg kg^−1^, subcutaneously). Catheters were flushed daily with heparin (Heparin ‘Bichsel’) in saline (30 IU) during the recovery period, before and after each self-administration session.

#### Stereotaxic injections

Injections were carried out as previously described^35,36^. Adeno-associated virus AAV8-hSyn-Flex-ChrimsonR-tdTomato (ETH Zurich Vector Core, cat. no. 289-8) was injected into the VTA of mice aged 5–8 weeks. Anesthesia was induced at 5% and maintained at 2.5% isoflurane (w:v) (Baxter AG) during surgery. The mouse was placed in a stereotaxic frame (Angle One) and craniotomies were performed using stereotaxic coordinates (for VTA: anteroposterior (AP) −3.3, mediolateral (ML) −0.9 with a 10° angle and dorsoventral (DV) −4.3). Following the same procedure, AAV5-hSyn-dLight1.2 (Addgene. cat. no. 111068) was injected unilaterally in the NAc (AP +1.6, ML +0.7 and DV −4.3). Virus injections (0.5 μl) were made with graduated pipettes (Drummond Scientific Co.), broken back to a tip diameter of 10–15 μm, at an infusion rate of 0.05 μl min^−1^. During the same surgical procedure, a chronically indwelling optic fiber cannula (Inper, cat. no. FOC-W-1.25-200-0.37-5.0) was implanted above the VTA using the same coordinates as for the injection except for the DV coordinate, which was reduced to 4.2. A photometry fiber (Inper, cat. no. FOC-W-2.5-400-0.5-6.0) was implanted unilaterally in the NAc (AP +1.6, ML +0.7 and DV −4.15) for dLight recordings. Three screws drilled into the skull fixed the implant and were secured with dental cement. The first behavioral session typically occurred 30–40 d after surgery to allow sufficient expression of dLight and Chrimson.

### Tissue preparation for imaging

Mice were anesthetized with pentobarbital (300 mg kg^−1^, intraperitoneally; Sanofi-Aventis) and transcardially perfused with 4% (w:v) paraformaldehyde in phosphate-buffered saline, pH 7.5. Brains were post-fixed overnight and stored at 4 °C. Coronal sections (60-µm thick) were cut with a vibratome (Leica), stained with Hoechst (Sigma-Aldrich) and mounted with Mowiol (Sigma-Aldrich). Full images of brain slices from VTA and NAc were obtained with a Zeiss Axioscan Z1 system equipped with a Plan-Apochromat ×10 and 0.45 numerical aperture objective, together with filters for DAPI (emission band-pass filter: 445-nm center wavelength and a 50-nm full-width at half-maximum bandwidth (445/50 nm)), eGFP (emission band-pass filter: 525/50 nm) and cyanine 3 (Cy3) (emission band-pass filter: 605/70 nm). For immunohistochemistry, the primary antibody (rabbit polyclonal anti-GFP, Invitrogen, cat. no. A11122, lot 2481666, 1:500) and the secondary antibody (donkey anti-rabbit Alexa Fluor-488, Invitrogen, cat. no. A21206, lot 19277937, 1:500) were used. The dLight infection highlighted by the GFP staining amplification was observed in the dorsomedial shell and in the core of the NAc in all recorded mice. Mice with weak VTA or NAc infection were excluded from the study.

### Optogenetic self-stimulation apparatus

Mice were placed in operant chambers (Med Associates, cat. no. ENV-307A-CT) situated in a sound-attenuating box (Med Associates). The optic fiber of the mouse was connected to DPSS orange-light lasers (Laser2000, cat. no. MGL-N-593.5-300mW and a shutter, Doric Lenses, cat. no. CMSA-SR475-FC) via a ferrule connector/physical contact (FC/PC) fiber cable (Inper, cat. no. MFO-F-W1.25-200-0.37-100) and a simple rotary joint (Doric Lenses, cat. no. FRJ-FC-FC) allowing free movement during operant behavior. The power at the exit of the patch cord was set to 15 ± 1 mW.

Optogenetic stimulation short-oDASS and long-oDASS consisted, respectively, of 2 and 30 bursts of 5 laser pulses (10 ms) at 20 Hz, 500 ms apart. Optogenetic stimulation with repeated pulses of 10 ms (for 20 pulses or 2 s) was also tested with various frequencies (5 Hz, 10 Hz, 20 Hz or 50 Hz) while recording with dLight in NAc.

### Operant box

The mouse operant chambers contained two retractable levers, a cue light located above each lever (Med Associates, cat. no. ENV-321 or APEM LEDs, cat. no. APC001), one speaker (Med Associates, cat. no. ENV-323AM) located behind the wall, the infusion pump (Med Associates, cat. no. PHM100), the spout located in the center of the wall, between the two levers, the liquid dispenser (Asco, cat. no. SCH284B001.24/DC) and the lickometer (Med Associates, cat. no. ENV-250). A rack mount interface cabinet (Med Associates, cat. no. SG-6010A) containing a programmable constant current shocker (Med Associates, cat. no. ENV-413) connected to a grid harness (Med Associates, cat. no. ENV-307A-QD) was used to provide foot-shocks. The apparatus was controlled and data captured using MED-PC 5.1 software (Med Associates).

### Behaviors

#### Acquisition of cocaine self-administration

During intravenous cocaine self-administration sessions, the stainless steel tubing of the catheter device was connected through a CoEx poly(ethylene) or poly(vinyl chloride) tubing (Phymep, cat. no. BCOEX-22) to a 22GA swivel (Instech Solomon, cat. no. 375/22PS) and then to an infusion pump. During six daily sessions, a single press (fixed ratio 1 (FR1)) on the active lever resulted in an infusion of cocaine (cocaine hydrochloride, Geneva University Hospital, dissolved in 0.9% saline at 0.75 mg ml^−1^ or 1.25 mg ml^−1^ and delivered at 0.0177 ml s^−1^ as a unit dose depending on the weight of the mouse). Infusion triggered a timeout period of 20 s, during which the cocaine was no longer available.

Mice then received sampling trials for 3 d with an ITI reduced to 60 s to allow a cumulative effect between successive infusions during the learning phase.

#### Sampling of cocaine self-administration

Sampling trials started with the presentation of a discriminative cue (auditory or visual), which terminated at the time of Le. The cue associated with the NR was either auditory or visual (randomized attributed to each mouse for the duration of the experiment) and the other modality was used for the alternative option (cocaine). Cues are either a small light (orange 0.79-cm diameter) or tone (80 dB, 2.9 kHz), both lasting for 2 s. Pressing the lever triggered its immediate retraction (within 0.5 s) and the infusion pump 5 s later (0.75 or 1.25 mg kg^−1^ per infusion). After Le, mice had a maximum delay of 260 s to P before the lever was automatically retracted and the trial was considered as a miss. Infusion parameters were identical to those described in the previous self-administration section. The ITI was then set to 4 min or 6 min depending on the dose of cocaine used during the sessions with choice trials.

#### Sampling trials for NRs and oDASS

Mice were placed in the operant chamber for the first time and dLight fiber photometry was recorded during the entire session. Importantly mice were not food or water deprived in any experiment. The novice period started from the first trial and ended with a session containing at least one success trial. The expert period started when press accuracy was >75% in two successive sessions. Sampling trials performed between these two periods were considered as the intermediate period. The dLight photometry was recorded during the novice period and occasionally during the intermediate and expert periods. A recorded session consisted of 12–20 lever presentations (also called Les) or a maximum duration of 45 min, whichever came first (one session per day for each of the two outcomes, that is. NR or short-oDASS or long-oDASS). After the novice period, mixed sampling sessions were introduced in which sampling trials for each reward were given in a random order. The learning phase consisted of a minimum of 20 sessions for each reward. Before Le, a discriminative cue indicating the identity of the possible reward was turned on for 2 s. The cue associated with the NR was either auditory or visual (randomized attributed to each mouse for the duration of the experiment) and the other modality was used for the alternative option (oDASS). Small light (orange 0.79-cm diameter) or tone (80 dB, 2.9 kHz), both lasting for 2 s, were used as cues before the Le.

After Le, mice had a maximum delay of 260 s to press before the lever was retracted. A trial was considered as missed whenever no press was made within 260 s. Press accuracy was calculated as the rate of hit trials. The press led to the immediate retraction of the lever and to the Rd with a 5-s delay. Rd consisted of optogenetic stimulation (2 or 30 bursts of 5 pulses at 20 Hz, 500 ms apart for, respectively, short-oDASS or long-oDASS). For the NR, the delivery consisted of a valve opening for 0.15 s that led to a drop of Lipofundin at the tip of the spout. When collecting the drop by licking the spout, an additional valve opening of 0.5 s happened to deliver a bigger drop of NR after three licks. This was done to prevent the drop from falling in the absence of licks. The volume received per trial was 24 µl. No reduction in performance was detected during a session. The iITI had a duration of 90 s after the end of Rd in a success trial or after the lever retraction in a missed trial.

#### Reward omission experiments

To further validate that photometry recordings of DA transients in the NAc are capturing the main RPE-like properties of VTA DA neurons in this operant schedule, we next tested whether reward omission in expert mice could produce a deflection at the time the reward is expected, here 5 s after the lever press. Sessions of short-oDASS, long-oDASS and NR sampling trials were performed in 7–12 mice during which Rd (optogenetic stimulation or the valve opening) was omitted in 50% of the trials. Each mouse had a total of 24 trials for each reward.

#### Two-reward choice

Choice sessions were made 3–10× in mice expert for each reward. A daily choice session was composed of two blocks (separated by 10–15 min) of six sampling trials (three for each reward) and eight choice trials as previously described^4^. Data for all choice trials were pooled or separated in three epochs (early, mid and late) for graphic representations and clustering. Sampling trials were identical to those described above. Choice trials started by the simultaneous extension of the two levers (preceded by the specific visual and auditory cue for 2 s, in the corresponding experiments). First press on a lever immediately triggered the retraction of the two levers and the delivery of the chosen reward. Choice trial was considered as missed whenever no lever press was made within 260 s, the time after which both levers are retracted. The ITI was identical for choice and sampling trials. The schedule of choice sessions was identical for the different pairs of rewards (oDASS versus NR and cocaine versus NR). The preference score is the ratio of number of choices between the two rewards (*N*_NR_ − *N*_AR_)/(*N*_NR_ + *N*_AR_).

#### PR on the long-oDASS

Classically, in addiction models the perseverance of the drug seeking or taking with a risk of foot-shock, used as a punishment, serves to identify the compulsive-like individuals^35,36,63^. Here the risk of foot-shock was introduced in a second phase of the experiment after all mice experienced the choice trials for long-oDASS and NR without PR. Choice trials started immediately after, with the introduction of the PR. After mice underwent the choice sessions for long-oDASS versus NR, without PR, additional blocks of choice were given that contained six sampling (three for each reward) and eight choice trials in which long-oDASS was under PR. In these blocks lever press for oDASS occasionally triggered a foot-shock (500 ms, 0.25 mA) given immediately after the press while optogenetic stimulation onset remained 5 s later. Every third long-oDASS sampling trial, starting with the second trial of the block, was punished. Similarly, every third long-oDASS choice was punished. In the case of a missed trial, the schedule of punished trials and nonpunished trials was postponed. DA transients were analyzed during sampling trials and reward preference was analyzed from choice trials, during two successive testing conditions, first without and then with a PR on long-oDASS.

#### Extended training for short-oDASS

After mice underwent the choice sessions for short-oDASS versus NR, they were extensively trained for short-oDASS sampling before a second period of choice sessions. The extended training consisted of daily sessions of 30–50 short-oDASS sampling trials for 1 month. Mice also received NR sampling trial sessions and mixed sampling trial sessions in which short-oDASS and NR sampling trials were given in a random order. The dLight photometry was performed during the last week of the extended training and the second period of choice sessions. Some mice of the extended training experiment were used for the cocaine versus NR choice experiment.

#### Perseverance of oDASS

Each of the 12 acquisition sessions lasted 60 min, during which mice could respond on the active lever to trigger the optogenetic stimulation (oDASS, 30 bursts of 5 pulses at 20 Hz, 500 ms apart), after a delay of 5 s. Each pulse had a 10-ms duration. During the first four sessions, a single press on the active lever (termed FR1) was required. Next, an FR2 (sessions 5–8) and an FR3 (sessions 9–12) were introduced. A 20-s time-out followed the rewarded lever press, during which lever presses had no consequences. Pressing the inactive lever also had no consequences. After this acquisition phase, mice underwent three additional sessions with a reduced duration (30 min). These sessions served as a baseline before starting the punishment sessions. Punishment sessions occurred in the same conditions as for baseline sessions, except every third laser stimulation event was preceded by a foot-shock (500 ms, 0.25 mA), starting immediately after the completion of FR3. Impending punishment was announced by illumination of the home cage light for 1 s after the second press of FR3. Perseverance was determined as previously described^32,36^.

#### Progressive ratio

For measurements of motivation, mice did a single progressive ratio session that lasted for a maximum of 4 h. The breakpoint is reported as the last ratio completed during the single experimental session of 4 h or after 40 min had elapsed since the last schedule. The reinforced schedules were the following: 1, 3, 5, 8, 12, 16, 22, 29, 38, 50, 65, 84, 108, 139, 178, 228, 291, 371, 473, 603, 767, 977, 1,243 and 1,582. The progressive ratio experiment was done for long-oDASS and NR.

### Photometry recordings

Data were collected blind with TDT Synapse v98-528 (Tucker Davis). During recordings, the NAc was illuminated with blue (470-nm wavelength, Thorlabs, cat. no. M470F3) and violet (405-nm wavelength, Thorlabs, cat. no. M405FP1) filtered excitation light-emitting diode lights, which were sinusoidally modulated at 211 Hz and 531 Hz and then focused on to a patch cord. Green emission light (500–550 nm) was collected through the same fiber that was used for excitation and passed on to a photoreceiver (Doric Lenses, cat. no. Newport 2151). Pre-amplified signals were then demodulated by a real-time signal processor (Tucker Davis Systems, cat. no. RZ5P) to determine contributions from 470-nm and 405-nm excitation sources^75^. The data acquisition rate was made at 1,017.25 Hz. Transistor–transistor logic signals of the relevant stimuli were directly sent from the operant chamber to the signal processor.

### Data processing

Data analysis was performed blind to the conditions of the experiments. The data were analyzed using MATLAB (MathWorks, cat. no. R2023b). To calculate Δ*F*/*F*_0_, a linear fit was applied to the 405-nm control signal to align it to the 470-nm signal. This fitted 405-nm signal was used as *F*_0_ in standard Δ*F*/*F*_0_ normalization (*F*(*t*) − *F*_0_(*t*))/*F*_0_(*t*). The original data were recorded with a sampling rate of 1,017.4 Hz. For convenience, we resampled the data to get a rate of 100 Hz. This was done using the MATLAB function ‘resample’. To improve comparison of photometry data, between animals and across sessions, we normalized data to the spontaneous DA fluctuations detected in baseline periods within each recording. Spontaneous DA peaks were defined as local peaks of dLight signal (Δ*F*/*F*_0_) with an amplitude of at least 0.2% Δ*F*/*F*_0_. In the detection of spontaneous events, we ignored the data 5 s before and 30 s after any imposed external event, like Le or Rd. We selected the top 10% spontaneous events and used their mean amplitude to rescale the whole trace of photometry recording (Extended Data Fig. 1). Next, the data were aligned to two different events: the Le and the P (when applicable) and data from 20 s before to 30 s after each of these events were stored. For learning NR sampling, the window was extended to 45 s to capture drop collection. For experiments involving cocaine, this time-window was increased to capture the lasting effect of cocaine (from 60 s before to 120 s after Le and P). For each trial, the photometry data were extracted using customized MATLAB scripts and exported as Excel files

Next, a baseline of the normalized dLight signal, defined as the mean during a window (−3.5 s to −0.5 s) before the cue was computed for each trial and subtracted from the whole trial. For trials with a long (>15 s) delay, to avoid a shift in baseline between the cue and the press, the baseline for the data aligned to press was computed 4 s before the press, for a duration of 3 s.

The normalized dLight recordings were sorted according to the delay to press and plotted in heatmaps, around the Le or around the P (−20 s to +30 s or +45 s, or 120 s after the Le in experiments with cocaine). Plots for mean traces were made using Igor 9.05.

The DA transients recorded with dLight were obtained, for each mouse, in sampling trials for each reward, often during the same session, to avoid technical differences on the photometry signals. Amplitude can therefore be directly compared. AUCs for NR and AR sampling at each event in imposed time windows (for discriminative cue from onset +2 s, for the Le, Le from onset +1 s, for P from onset +1 s, for P to the Rd, Rd window for the 5-s and post-reward windows, from Rd +1 to 115 s) were taken (units: normalized dLight s^−1^). Differences of the mean AUC between NR and AR of the normalized dLight in the different time-windows of the schedule were calculated and used for correlation with the preference score.

### Clustering method

A hierarchical clustering was applied for each choice experiment to separate groups behaving differently. Before the clustering, we applied a nonlinear dimension reduction algorithm (*t*-distributed stochastic neighbor embedding)^76^, on the variables of interest (accuracy and mean delay for both sampling and choice trials, preference index) to end up with two or three relevant projected variables. We then used a hierarchical clustering algorithm (with the Minkowski metric and average linkage method, using the MATLAB ‘pdist’, ‘linkage’ and ‘cluster’ functions) to divide the mice into two or three clusters, based on their overall behavioral similarities. The dimension reduction and clustering were repeated iteratively 100× and the best tree was taken based on the clustering robustness, namely the mean silhouette score and the Cophenetic distance. The resulting mean silhouette value (computed using the MATLAB ‘silhouette’ function) was between 0.68 and 0.84, indicating that the clustering solution is appropriate. The preference index, computed separately for each animal, was defined as (*N*_NR_ − *N*_AR_)/(*N*_NR_ + *N*_AR_). Omissions were not considered in the preference index.

### Statistics

Data distribution was assumed to be normal without formal testing. Single comparisons of between-group or within-group measures were made by two-tailed, nonpaired or paired Student’s *t*-tests, respectively. Spearman’s correlation was used to test significant correlations with the preference score. Multiple comparisons were first subjected to mixed-factor ANOVA defining both between-group and/or within-group factors. Where significant main effects or interaction terms were found (*P* ≤ 0.05), further comparisons were made using a two-tailed Student’s *t*-test with Bonferroni’s correction applied when appropriate (that is, the level of significance = 0.05 divided by the number of comparisons). For comparison of AUCs across learning periods, an ANOVA for RMs was used and, where significant main was found (*P* ≤ 0.05), further comparisons were made using a post-hoc comparison with Dunnett’s test. Samples sizes were predetermined using a power and sample size calculator when possible or sample sizes were based on MonteCarlo simulations and previous publications^36,61^. Statistics were calculated using GraphPad Prism v11 and fully reported in Supplementary Table 1.

### Reporting summary

Further information on research design is available in the Nature Portfolio Reporting Summary linked to this article.

## Online content

Any methods, additional references, Nature Portfolio reporting summaries, source data, extended data, supplementary information, acknowledgements, peer review information; details of author contributions and competing interests; and statements of data and code availability are available at 10.1038/s41593-026-02331-y.

## Supplementary information

Supplementary Information Supplementary Table for statistical reporting of data of Figs 1–7 and Extended Data Figs. 1–10.

Reporting Summary

## Data Availability

Data are available via Zenodo at 10.5281/zenodo.15463589 (ref. ^77^).

## Code availability

Codes are available via Zenodo at 10.5281/zenodo.15463589 (ref. ^77^).
