# Supplementary material for: Conditioned accumbal dopamine transients forecast individual preference for drug versus natural rewards and compulsive behavior
Source: Nat Neurosci. 2026 Jun 26;29(8):1905–19. doi: 10.1038/s41593-026-02331-y (PMC13433266; doi:10.1038/s41593-026-02331-y)
Supplement: Supplementary file 1 [file 41593_2026_2331_MOESM1_ESM.pdf]

nature neuroscience

Supplementary information

https://doi.org/10.1038/s41593-026-02331-y

Conditioned accumbal dopamine transients forecast individual preference for drug versus natural rewards and compulsive behavior

In the format provided by the
authors and unedited

Statistic table for Main figures

Figure	Experiment	Variable	Comparison	Test	Effect	Test result	P	Effect size
1g	NR *versus* short-oDASS learning	Norm. dLight AUC CUE	Novice / Intermediate / Expert<br>NR / short-oDASS	Two-way ANOVA for RM	Learning period<br>Reward type<br>Learning period x Reward type	$F_{(2,74)}$ = 51.23<br>$F_{(1,37)}$ = 0.31<br>$F_{(2,74)}$ = 5.82	<0.001<br>0.5802<br>0.0045	η^2_p = 0.581<br>η^2_p = 0.008<br>η^2_p = 0.136
				Bonferroni	Novice: NR / short-oDASS<br>Intermediate: NR / short-oDASS<br>Expert: NR / short-oDASS<br>NR: novice / intermediate<br>NR: novice / expert<br>short-oDASS: novice / interm.<br>short-oDASS: novice / expert	t_{37} = 0.78<br>t_{37} = 0.87<br>t_{37} = 3.35<br>t_{37} = 2.55<br>t_{37} = 7.91<br>t_{37} = 2.64<br>t_{37} = 3.77	0.4381<br>0.3877<br>0.0013<br>0.0388<br><0.001<br>0.0306<br>0.0010	
1g	NR *versus* short-oDASS learning	Norm. dLight AUC Le	Novice / Intermediate / Expert<br>NR / short-oDASS	Two-way ANOVA for RM	Learning period<br>Reward type<br>Learning period x Reward type	$F_{(2,74)}$ = 98.21<br>$F_{(1,37)}$ = 2.83<br>$F_{(2,74)}$ = 6.20	<0.001<br>0.1008<br>0.0032	η^2_p = 0.726<br>η^2_p = 0.071<br>η^2_p = 0.144
				Bonferroni	Novice: NR / short-oDASS<br>Intermediate: NR / short-oDASS<br>Expert: NR / short-oDASS<br>NR: novice / intermediate<br>NR: novice / expert<br>short-oDASS: novice / interm.<br>short-oDASS: novice / expert	t_{37} = 0.40<br>t_{37} = 0.72<br>t_{37} = 4.36<br>t_{37} = 4.58<br>t_{37} = 11.63<br>t_{37} = 3.46<br>t_{37} = 6.87	0.6917<br>0.4760<br><0.001<br><0.001<br><0.001<br>0.0027<br><0.001	
1g	NR *versus* short-oDASS learning	Norm. dLight AUC Press-Rd	Novice / Intermediate / Expert<br>NR / short-oDASS	Two-way ANOVA for RM	Learning period<br>Reward type<br>Learning period x Reward type	$F_{(2,74)}$ = 42.86<br>$F_{(1,37)}$ = 19.14<br>$F_{(2,72)}$ = 1.23	<0.001<br><0.001<br>0.2992	η^2_p = 0.537<br>η^2_p = 0.341<br>η^2_p = 0.033
				Bonferroni	Novice: NR / short-oDASS<br>Intermediate: NR / short-oDASS<br>Expert: NR / short-oDASS<br>NR: novice / intermediate<br>NR: novice / expert<br>short-oDASS: novice / interm.<br>short-oDASS: novice / expert	t_{37} = 1.68<br>t_{37} = 2.12<br>t_{37} = 3.80<br>t_{36} = 4.05<br>t_{37} = 8.17<br>t_{37} = 3.71<br>t_{37} = 6.38	0.0968<br>0.0365<br><0.001<br><0.001<br><0.001<br><0.001<br><0.001	
1g	NR *versus* short-oDASS learning	Norm. dLight AUC Rd+1s	Novice / Intermediate / Expert<br>NR / short-oDASS	Two-way ANOVA for RM	Learning period<br>Reward type<br>Learning period x Reward type	$F_{(2,74)}$ = 14.13<br>$F_{(1,37)}$ = 95.90<br>$F_{(2,74)}$ = 2.53	<0.001<br><0.001<br>0.0870	η^2_p = 0.276<br>η^2_p = 0.722<br>η^2_p = 0.066
				Bonferroni	Novice: NR / short-oDASS<br>Intermediate: NR / short-oDASS<br>Expert: NR / short-oDASS<br>NR: novice / intermediate<br>NR: novice / expert<br>short-oDASS: novice / interm.<br>short-oDASS: novice / expert	t_{37} = 8.66<br>t_{37} = 9.34<br>t_{37} = 7.45<br>t_{36} = 2.33<br>t_{37} = 4.51<br>t_{37} = 3.48<br>t_{37} = 2.53	<0.001<br><0.001<br><0.001<br>0.0642<br><0.001<br>0.0020<br>0.0378	
1g	NR *versus* short-oDASS learning	Norm. dLight AUC Rd_{full}	Novice / Intermediate / Expert<br>NR / short-oDASS	Two-way ANOVA for RM	Learning period<br>Reward type<br>Learning period x Reward type	$F_{(2,74)}$ = 28.44<br>$F_{(1,37)}$ = 0.39<br>$F_{(2,74)}$ = 27.06	<0.001<br>0.5357<br><0.001	η^2_p = 0.435<br>η^2_p = 0.010<br>η^2_p = 0.429
				Bonferroni	Novice: NR / short-oDASS<br>Intermediate: NR / short-oDASS<br>Expert: NR / short-oDASS<br>NR: novice / intermediate<br>NR: novice / expert<br>short-oDASS: novice / interm.<br>short-oDASS: novice / expert	t_{37} = 4.60<br>t_{37} = 2.75<br>t_{37} = 3.23<br>t_{36} = 7.44<br>t_{37} = 9.90<br>t_{37} = 1.22<br>t_{37} = 0.68	<0.001<br>0.0071<br>0.0016<br><0.001<br><0.001<br>0.6708<br>>0.999	
1h	Learning NR	Norm. dLight AUC CUE expert<br>Norm. dLight AUC maximum Rd_{full} novice	Norm. dLight AUC CUE (expert) / Norm. dLight AUC Rd_{full} Max. (novice)	Spearman's correlation		r_{36} = 0.151	0.3658	95%CI [-0.187,-0.457]
1h	Learning short-oDASS	Norm. dLight AUC CUE expert<br>Norm. dLight AUC maximum Rd_{full} novice	Norm. dLight AUC CUE (expert) / Norm. dLight AUC Rd_{full} Max. (novice)	Spearman's correlation		r_{36} = 0.023	0.8895	95%CI [-0.308,0.349]
1m	Choice NR / short-oDASS	Preference score	NR_{pref} / No_{pref} / short-$oDASS_{pref}$	One-way ANOVA		$F_{(2,65)}$ = 282.80	<0.001	η^2_p = 0.897
				Bonferroni	NR_{pref} / No_{pref}<br>NR_{pref} / short-$oDASS_{pref}$<br>No_{pref} / short-$oDASS_{pref}$	t_{43} = 12.70<br>t_{38} = 23.53<br>t_{39} = 13.46	<0.001<br><0.001<br><0.001	
1o	Choice NR / short-oDASS	Norm. dLight AUC CUE	NR_{pref} / No_{pref} / short-$oDASS_{pref}$	One-way ANOVA		$F_{(2,65)}$ = 18.37	<0.001	η^2_p = 0.361
				Bonferroni	NR_{pref} / No_{pref}<br>NR_{pref} / short-$oDASS_{pref}$<br>No_{pref} / short-$oDASS_{pref}$	t_{43} = 2.02<br>t_{38} = 6.04<br>t_{39} = 4,45	0.1420<br><0.001<br><0.001	
1q	Choice NR / short-oDASS	Norm. dLight AUC Rd_{full}	NR_{pref} / No_{pref} / short-$oDASS_{pref}$	One-way ANOVA		$F_{(2,65)}$ = 2.63	0.0796	η^2_p = 0.075
1r	Choice NR / short-oDASS	Preference score<br>Norm. dLight AUC CUE (NR-oDASS)	Preference score / Norm. dLight AUC CUE (NR - oDASS)	Spearman's correlation		r_{66} = 0.554	<0.001	95%CI [0.357,0.704]
1r	Choice NR / short-oDASS	Preference score<br>Norm. dLight AUC Rd_{full}	Preference score / Norm. dLight AUC Rd_{full} (NR - oDASS)	Spearman's correlation		r_{66} = -0.181	0.1398	95%CI [-0.408,0.067]
2c	Re-valuation	Preference score	NR_{pref} / $Switch_{pref}$ / $oDASS_{pref}$<br>short-oDASS / long-oDASS	Two-way ANOVA for RM	Cluster<br>oDASS protocol<br>Cluster x oDASS protocol	$F_{(2,24)}$=81.17<br>$F_{(1,24)}$=69.09<br>$F_{(2,24)}$=10.52	<0.001<br><0.001<br>0.0005	η^2_p = 0.871<br>η^2_p = 0.742<br>η^2_p = 0.467
				Bonferroni	short-oDASS: NR_{pref} / $Switch_{pref}$<br>short-oDASS: NR_{pref} / $oDASS_{pref}$<br>short-oDASS: $Switch_{pref}$ / $oDASS_{pref}$<br>long-oDASS: NR_{pref} / $Switch_{pref}$<br>long-oDASS: NR_{pref} / $oDASS_{pref}$<br>long-oDASS: $Switch_{pref}$ / $oDASS_{pref}$<br>NR_{pref}: short / long-oDASS<br>$Switch_{pref}$: short / long-oDASS<br>$oDASS_{pref}$: short / long-oDASS	t_{18} = 4.33<br>t_{11} = 9.92<br>t_{19} = 7.36<br>t_{18} = 8.19<br>t_{11} = 8.27<br>t_{19} = 1.30<br>t_{5} = 3.63<br>t_{13} = 10.59<br>t_{6} = 2.17	<0.001<br><0.001<br><0.001<br><0.001<br><0.001<br>0.5961<br>0.0013<br><0.001<br>0.0403	

Figure	Experiment	Variable	Comparison	Test	Effect	Test result	*P*	Effect size
2e	Re-valuation	Norm. dLight AUC CUE (NR-oDASS)	NR_{pref} / $Switch_{pref}$ / $oDASS_{pref}$<br>short-oDASS / long-oDASS	Two-way ANOVA for RM	Cluster<br>oDASS protocol<br>Cluster x oDASS protocol	$F_{(2,24)}$ = 7.87<br>$F_{(1,24)}$ = 92.72<br>$F_{(2,24)}$ = 7.80	0.0023<br><0.001<br>0.0025	η^2_p = 0.396<br>η^2_p = 0.794<br>η^2_p = 0.394
				Bonferroni	short-oDASS: NR_{pref} / $Switch_{pref}$<br>short-oDASS: NR_{pref} / $oDASS_{pref}$<br>short-oDASS: $Switch_{pref}$ / $oDASS_{pref}$<br>long-oDASS: NR_{pref} / $Switch_{pref}$<br>long-oDASS: NR_{pref} / $oDASS_{pref}$<br>long-oDASS: $Switch_{pref}$ / $oDASS_{pref}$<br>NR_{pref}: short- / long-oDASS<br>$Switch_{pref}$: short- / long-oDASS<br>$oDASS_{pref}$: short- / long-oDASS	t_{18} = 1.00<br>t_{11} = 3.53<br>t_{19} = 3.19<br>t_{18} = 4.00<br>t_{11} = 3.15<br>t_{19} = 0.44<br>t_{5} = 3.87<br>t_{13} = 11.17<br>t_{6} = 3.64	0.9649<br>0.0028<br>0.0076<br><0.001<br>0.0085<br>>0.999<br><0.001<br><0.001<br>0.0013	
2f	Re-valuation	Preference score<br>Norm. dLight AUC CUE	Preference score / Norm. dLight AUC CUE (NR - short-oDASS)	Spearman's correlation		r_{25} = 0.681	<0.001	95%CI [0.396,0.846]
2f	Re-valuation	Preference score<br>Norm. dLight AUC Rd_{full}	Preference score / Norm. dLight AUC Rd_{full} (NR - short-oDASS)	Spearman's correlation		r_{25} = -0.311	0.1146	95%CI [-0.625,0.090]
2g	Re-valuation	Preference score<br>Norm. dLight AUC CUE	Preference score / Norm. dLight AUC CUE (NR - long-oDASS)	Spearman's correlation		r_{25} = 0.528	0.0047	95%CI [0.173,0.761]
2g	Re-valuation	Preference score<br>Norm. dLight AUC Rd_{full}	Preference score / Norm. dLight AUC Rd_{full} (NR - long-oDASS)	Spearman's correlation		r_{25} = 0.118	0.5583	95%CI [-0.285,0.486]
3c	Extended oDASS training	Preference score	NR_{pref} / No_{pref}<br>expert / over-trained short-oDASS	Two-way ANOVA for RM	Cluster<br>Extended training<br>Cluster x Extended training	$F_{(1,27)}$ = 70.42<br>$F_{(1,27)}$ = 2.20<br>$F_{(1,27)}$ = 4.58	<0.001<br>0.1493<br>0.0416	η^2_p = 0.723<br>η^2_p = 0.075<br>η^2_p = 0.145
				Uncorrected Fisher's LSD	Expert: NR_{pref} / No_{pref}<br>Over-trained: NR_{pref} / No_{pref}<br>NR_{pref}: Expert / over-trained<br>No_{pref}: Expert / over-trained	t_{27} = 7.78<br>t_{27} = 5.01<br>t_{19}=0.59<br>t_{8} = 2.18	<0.001<br><0.001<br>0.5613<br>0.0380	
3e	Extended oDASS training	Norm. dLight AUC CUE (NR-oDASS)	NR_{pref} / No_{pref}<br>Expert /over-trained short-oDASS	Two-way ANOVA for RM	Cluster<br>Extended oDASS training<br>Cluster x Extended oDASS training	$F_{(1,27)}$ = 9.21<br>$F_{(1,27)}$ = 0.31<br>$F_{(1,27)}$ = 1.20	0.0053<br>0.5833<br>0.2823	η^2_p = 0.254<br>η^2_p = 0.011<br>η^2_p = 0.043
				Uncorrected Fisher's LSD	Expert: NR_{pref} / No_{pref}<br>Over-trained: NR_{pref} / No_{pref}<br>NR_{pref}: Expert / over-trained<br>No_{pref}: Expert / over-trained	t_{27} = 2.24<br>t_{27} = 3.21<br>t_{19} = 1.48<br>t_{8} = 0.33	0.0294<br>0.0023<br>0.1497<br>0.7468	
3f	Expert short-oDASS	Preference score<br>Norm. dLight AUC CUE	Preference score / Norm. dLight AUC CUE (NR - short-oDASS)	Spearman's correlation		r_{27} = 0.472	0.0098	95%CI [0.116,0.720]
3f	Expert short-oDASS	Preference score<br>Norm. dLight AUC Rd_{full}	Preference score / Norm. dLight AUC Rd_{full} (NR - short-oDASS)	Spearman's correlation		r_{27} = -0.031	0.8730	95%CI [-0.403,0.349]
3g	Over-trained short-oDASS	Preference score<br>Norm. dLight AUC CUE	Preference score / Norm. dLight AUC CUE (NR - short-oDASS)	Spearman's correlation		r_{27} = 0.618	<0.001	95%CI [0.315,0.807]
3g	Over-trained short-oDASS	Preference score<br>Norm. dLight AUC Rd_{full}	Preference score / Norm. dLight AUC CUE (NR - short-oDASS)	Spearman's correlation		r_{27} = 0.118	0.5413	95%CI [-0.270,0.474]
4i	Choice Cocaine 1.25 mg/kg/inf. / NR	Preference score	NR_{pref} / No_{pref}	Unpaired *t*-test	Two-tailed	t_{47} = 11.98	<0.001	MD = -0.769, 95%CI [-0.898,-0.640]
4k	Choice Cocaine 1.25 mg/kg/inf. / NR	Norm. dLight AUC CUE (NR-Cocaine)	NR_{pref} / No_{pref}	Unpaired *t*-test	Two-tailed	t_{47} = 4.29	<0.001	MD = -0.686, 95%CI [-1.008,-0.364]
4m	Choice Cocaine 1.25 mg/kg/inf. / NR	Preference score<br>Norm. dLight AUC CUE	Preference score / Norm. dLight AUC CUE (NR-Cocaine)	Spearman's correlation		r_{47} = 0.600	<0.001	95%CI [0.376,0.758]
4m	Cocaine 1.25 mg/kg/inf.	Preference score<br>Norm. dLight AUC Rd_{full}	Preference score / Norm. dLight AUC Rd_{full} (NR-Cocaine)	Spearman's correlation		r_{47} = 0.233	0.1076	95%CI [-0.060,0.489]
5f	NR *versus* long-oDASS learning	Norm. dLight AUC CUE	Novice / Intermediate / Expert<br>NR / long-oDASS	Two-way ANOVA for RM	Learning period<br>Reward type<br>Learning period x Reward type	$F_{(2,24)}$ = 73.95<br>$F_{(1,12)}$ = 37.08<br>$F_{(2,24)}$ = 17.71	<0.001<br><0.001<br><0.001	η^2_p = 0.860<br>η^2_p = 0.371<br>η^2_p = 0.596
				Bonferroni	Novice: NR / long-oDASS<br>Intermediate: NR / long-oDASS<br>Expert: NR / long-oDASS<br>NR: novice / intermediate<br>NR: novice / expert<br>long-oDASS: novice / interm.<br>long-oDASS: novice / expert	t_{12} = 0.42<br>t_{12} = 7.20<br>t_{12} = 8.13<br>t_{12} = 1.16<br>t_{12} = 2.40<br>t_{12} = 7.94<br>t_{12} = 10.11	0.6779<br><0.001<br><0.001<br>0.7744<br>0.0739<br><0.001<br><0.001	
5f	NR *versus* long-oDASS learning	Norm. dLight AUC at Le	Novice / Intermediate / Expert<br>NR / long-oDASS	Two-way ANOVA for RM	Learning period<br>Reward type<br>Learning period x Reward type	$F_{(2,24)}$ = 107.1<br>$F_{(1,12)}$ = 21.47<br>$F_{(2,24)}$ = 17.37	<0.001<br><0.001<br><0.001	η^2_p = 0.899<br>η^2_p = 0.641<br>η^2_p = 0.591
				Bonferroni	Novice: NR / long-oDASS<br>Intermediate: NR / long-oDASS<br>Expert: NR / long-oDASS<br>NR: novice / intermediate<br>NR: novice / expert<br>long-oDASS: novice / interm.<br>long-oDASS: novice / expert	t_{12} = 0.58<br>t_{12} = 7.32<br>t_{12} = 8.20<br>t_{12} = 3.40<br>t_{12} = 5.90<br>t_{12} = 10.14<br>t_{12} = 13.52	0.5660<br><0.001<br><0.001<br>0.0070<br><0.001<br><0.001<br><0.001	
5f	NR *versus* long-oDASS learning	Norm. dLight AUC at Press-Rd	Novice / Intermediate / Expert<br>NR / long-oDASS	Two-way ANOVA for RM	Learning period<br>Reward type<br>Learning period x Reward type	$F_{(2,24)}$ = 46.50<br>$F_{(1,12)}$ = 29.92<br>$F_{(2,24)}$ = 6.59	<0.001<br><0.001<br>0.0053	η^2_p = 0.795<br>η^2_p = 0.714<br>η^2_p = 0.354
				Bonferroni	Novice: NR / long-oDASS<br>Intermediate: NR / long-oDASS<br>Expert: NR / long-oDASS<br>NR: novice / intermediate<br>NR: novice / expert<br>long-oDASS: novice / interm.<br>long-oDASS: novice / expert	t_{12} = 0.26<br>t_{12} = 3.76<br>t_{12} = 5.27<br>t_{12} = 3.62<br>t_{12} = 5.99<br>t_{12} = 7.11<br>t_{12} = 11.00	0.7955<br>0.0010<br><0.001<br>0.0041<br><0.001<br><0.001<br><0.001	

Figure	Experiment	Variable	Comparison	Test	Effect	Test result	*P*	Effect size
5f	NR *versus* long-oDASS learning	Norm. dLight AUC Rd+1s	Novice / Intermediate / Expert<br>NR / long-oDASS	Two-way ANOVA for RM	Learning period<br>Reward type<br>Learning period x Reward type	$F_{(2,24)}$ = 15.44<br>$F_{(1,12)}$ = 173.20<br>$F_{(2,24)}$ = 1.03	<0.001<br><0.001<br>0.3733	η^2_p = 0.563<br>η^2_p = 0.935<br>η^2_p = 0.079
				Bonferroni	Novice: NR / long-oDASS<br>Intermediate: NR / long-oDASS<br>Expert: NR / long-oDASS<br>NR: novice / intermediate<br>NR: novice / expert<br>long-oDASS: novice / interm.<br>long-oDASS: novice / expert	t_{12} = 14.61<br>t_{12} = 12.92<br>t_{12} = 14.74<br>t_{12} = 2.81<br>t_{12} = 4.71<br>t_{12} = 1.12<br>t_{12} = 4.83	<0.001<br><0.001<br><0.001<br>0.0293<br><0.001<br>0.8233<br><0.001	
5f	NR *versus* long-oDASS learning	Norm. dLight AUC Rd_{full}	Novice / Intermediate / Expert<br>NR / long-oDASS	Two-way ANOVA for RM	Learning period<br>Reward type<br>Learning period x Reward type	$F_{(2,24)}$ = 8.33<br>$F_{(1,12)}$ = 67.68<br>$F_{(2,24)}$ = 2.01	0.0018<br><0.001<br>0.1562	η^2_p = 0.410<br>η^2_p = 0.849<br>η^2_p = 0.143
				Bonferroni	Novice: NR / long-oDASS<br>Intermediate: NR / long-oDASS<br>Expert: NR / long-oDASS<br>NR: novice / intermediate<br>NR: novice / expert<br>long-oDASS: novice / interm.<br>long-oDASS: novice / expert	t_{12} = 13.30<br>t_{12} = 14.60<br>t_{12} = 16.13<br>t_{12} = 5.00<br>t_{12} = 5.28<br>t_{12} = 3.70<br>t_{12} = 2.45	<0.001<br><0.001<br><0.001<br><0.001<br><0.001<br>0.0034<br>0.0655	
5g	Learning NR	Norm. dLight AUC CUE expert<br>Norm. dLight AUC maximum Rd_{full} novice	Norm. dLight AUC CUE (expert) / Norm. dLight AUC Rd_{full} Max. (novice)	Spearman's correlation		r_{11} = -0.060	0.8491	95%CI [-0.604,0.521]
5g	Learning long-oDASS	Norm. dLight AUC CUE expert<br>Norm. dLight AUC maximum Rd_{full} novice	Norm. dLight AUC CUE (expert) / Norm. dLight AUC Rd_{full} Max. (novice)	Spearman's correlation		r_{11} = 0.159	0.6040	95%CI [-0.444,0.663]
5k	Devaluation	Preference score	$Switch_{pref}$ / $oDASS_{pref}$<br>No Punishment Risk / PR	Two-way ANOVA for RM	Cluster<br>PR<br>Cluster x PR	$F_{(1,25)}$ = 45.41<br>$F_{(1,25)}$ = 164.4<br>$F_{(1,25)}$ = 94.53	<0.001<br><0.001<br><0.001	η^2_p = 0.645<br>η^2_p = 0.868<br>η^2_p = 0.791
				Uncorrected Fisher's LSD	no PR: $Switch_{pref}$ / $oDASS_{pref}$<br>PR: $Switch_{pref}$ / $oDASS_{pref}$<br>$Switch_{pref}$: No PR / PR<br>$oDASS_{pref}$: No PR / PR	t_{25} = 1.52<br>t_{25} = 10.45<br>t_{17} = 19.53<br>t_{8} = 1.90	0.1352<br><0.001<br><0.001<br>0.0692	
5m	Devaluation	Norm. dLight AUC CUE (NR-long-oDASS with PR)	$Switch_{pref}$ / $oDASS_{pref}$<br>No Punishment Risk / PR	Two-way ANOVA for RM	Cluster<br>PR<br>Cluster x PR	$F_{(1,25)}$ = 11.89<br>$F_{(1,25)}$ = 7.45<br>$F_{(1,25)}$ = 14.25	0.0020<br>0.0115<br><0.001	η^2_p = 0.322<br>η^2_p = 0.229<br>η^2_p = 0.363
				Uncorrected Fisher's LSD	no PR: $Switch_{pref}$ / $oDASS_{pref}$<br>PR: $Switch_{pref}$ / $oDASS_{pref}$<br>$Switch_{pref}$: No PR / PR<br>$oDASS_{pref}$: No PR / PR	t_{25} = 0.54<br>t_{25} = 5.02<br>t_{17} = 5.63<br>t_{8} = 0.64	0.5914<br><0.001<br><0.001<br>0.5276	
5n	Devaluation	Preference score<br>Norm. dLight AUC CUE	Preference score / Norm. dLight AUC CUE (NR - long-oDASS)	Spearman's correlation		r_{25} = 0.233	0.2423	95%CI [-0.173,0.571]
5n	Devaluation	Preference score<br>Norm. dLight AUC Rd_{full}	Preference score / Norm. dLight AUC Rd_{full} (NR - long-oDASS)	Spearman's correlation		r_{25} = 0.274	0.1664	95%CI [-0.130,0.600]
5o	Devaluation	Preference score<br>Norm. dLight AUC CUE	Preference score / Norm. dLight AUC CUE (NR - long-oDASS with PR)	Spearman's correlation		r_{25} = 0.675	<0.001	95%CI [0.387,0.843]
5o	Devaluation	Preference score<br>Norm. dLight AUC Rd_{full}	Preference score / Norm. dLight AUC Rd_{full} (NR - long-oDASS with PR)	Spearman's correlation		r_{25} = -0.136	0.5002	95%CI [-0.499,0.269]
6b	Behavorial marker	Breakpoint	NR / long-oDASS	Paired *t*-test	Two-tailed	t_{90} = 6.333	<0.001	MD = 67.47, 95%CI [46.30,88.64]
6c	Behavioral markers for long-oDASS and NR	Preference score<br>Breakpoint	Preference score / Breakpoint	Spearman's correlation		r_{89} = -0.134	0.2039	95%CI [-0.337,0.080]
6f	Choice and oDASS perseverance	Preference score (long-oDASS / NR)<br>Perseverance	Preference score (long-oDASS / NR) / Perseverance	Spearman's correlation		r_{122} = -0.113	0.2108	95%CI [-0.289,0.070]
6f	Choice and oDASS perseverance	Preference score (long-oDASS with PR / NR)<br>Perseverance	Preference score (long-oDASS with PR / NR) / Perseverance	Spearman's correlation		r_{98} = -0.584	<0.001	95%CI [-0.703,-0.433]
6g	Choice NR / long-oDASS and oDASS perseverance	Preference score	NR_{pref} / $oDASS_{pref}$	Unpaired *t*-test	Two-tailed	t_{122} = 15.36	<0.001	MD = -0.658, 95%CI [-0.743,-0.573]
6g	Choice NR / long-oDASS and oDASS perseverance	Perseverance	NR_{pref} / $oDASS_{pref}$	Unpaired *t*-test	Two-tailed	t_{122} = 2.41	0.0173	MD = 18.50, 95%CI [3.33,33.67]
6g	Choice NR / long-oDASS with PR and oDASS perseverance	Preference score	NR_{pref} / $oDASS_{pref}$	Unpaired *t*-test	Two-tailed	t_{98} = 18.97	<0.001	MD = -1.047, 95%CI [-1.156,-0.937]
6g	Choice NR / long-oDASS with PR and oDASS perseverance	Perseverance	NR_{pref} / $oDASS_{pref}$	Unpaired *t*-test	Two-tailed	t_{98} = 7.37	<0.001	MD = 40.63, 95%CI [29.69,51.58]
6h	Choice NR / long-oDASS and oDASS perseverance	Preference score	Renouncer / Perseverer	Unpaired *t*-test	Two-tailed	t_{122} = 1.89	0.0611	MD = -0.106, 95%CI [-0.217,0.005]
6h	Choice NR / long-oDASS and oDASS perseverance	Perseverance	Renouncer / Perseverer	Unpaired *t*-test	Two-tailed	t_{122} = 27.35	<0.001	MD = 62.20, 95%CI [57.70,66.70]
6h	Choice NR / long-oDASS with PR and oDASS perseverance	Preference score	Renouncer / Perseverer	Unpaired *t*-test	Two-tailed	t_{98} = 7.78	<0.001	MD = -0.705, 95%CI [-0.885,-0.525]
6h	Choice NR / long-oDASS with PR and oDASS perseverance	Perseverance	Renouncer / Perseverer	Unpaired *t*-test	Two-tailed	t_{98} = 24.85	<0.001	MD = 61.11, 95%CI [56.23,65.99]

Figure	Experiment	Variable	Comparison	Test	Effect	Test result	*P*	Effect size
7b	Choice NR / Cocaine 0.75 mg/kg/inf.	Preference score	NR / Cocaine 0.75 mg/kg/inf.	Unpaired *t*-test	Two-tailed	t_{23} = 12.17	<0.001	MD = -0.614, 95%CI [-0.719,-0.510]
7d	Choice NR / Cocaine 0.75 mg/kg/inf.	Norm. dLight AUC CUE (NR-Cocaine)	NR_{pref} / No_{pref}	Unpaired *t*-test	Two-tailed	t_{23} = 5.24	<0.001	MD = -0.935, 95%CI [-1.304,-0.565]
7f	Choice NR / Cocaine 0.75 mg/kg/inf.	Preference score<br>Norm. dLight AUC CUE	Preference score / Norm. dLight AUC CUE (NR-Cocaine)	Spearman's correlation		r_{23} = 0.582	0.0023	95%CI [0.230,0.799]
7f	Choice NR / Cocaine 0.75 mg/kg/inf.	Preference score<br>Norm. dLight AUC Rd_{full}	Preference score / Norm. dLight AUC Rd_{full} (NR-Cocaine)	Spearman's correlation		r_{23} = 0.225	0.2787	95%CI [-0.199,0.578]
7g	Choice NR / Cocaine 0.75 mg/kg/inf. and oDASS perseverance	Preference score (Cocaine 0.75 mg/kg/inf. / NR) Perseverance	Preference score (Cocaine 0.75 mg/kg/inf. / NR) Perseverance	Spearman's correlation		r_{52} = -0.478	<0.001	95%CI [-0.666,-0.233]
7h	Choice NR / Cocaine 0.75 mg/kg/inf. and oDASS perseverance	Preference score	NR_{pref} / No_{pref}	Unpaired *t*-test	Two-tailed	t_{52} = 10.85	<0.001	MD = -0.656, 95%CI [-0.777,-0.534]
7h	Choice NR / Cocaine 0.75 mg/kg/inf. and oDASS perseverance	Perseverance	NR_{pref} / No_{pref}	Unpaired *t*-test	Two-tailed	t_{52} = 3.99	0.0002	MD = 30.46, 95%CI [15.14,45.79]
7i	Choice NR / Cocaine 0.75 mg/kg/inf. and oDASS perseverance	Perseverance	Renouncer / Perseverer	Unpaired *t*-test	Two-tailed	t_{52} = 16.39	<0.001	MD = 60.16, 95%CI [52.79,67.52]
7i	Choice NR / Cocaine 0.75 mg/kg/inf. and oDASS perseverance	Preference score	Renouncer / Perseverer	Unpaired *t*-test	Two-tailed	t_{52} = 4.18	0.0001	MD = -0.412, 95%CI [-0.610,-0.214]
7m	Re-valuation	Preference score	NR_{pref} / No_{pref} / AR_{pref}<br>short-oDASS / Cocaine 1.25 mg/kg/inf.	Two-way ANOVA for RM	Cluster<br>Artificial Reward<br>Cluster x Artificial Reward	$F_{(2,26)}$= 60.25<br>$F_{(1,26)}$= 17.37<br>$F_{(2,26)}$= 6.78	<0.001<br>0.0003<br>0.0043	η^2_p = 0.823<br>η^2_p = 0.401<br>η^2_p = 0.343
				Bonferroni	short-oDASS:<br>NR_{pref} / $SWITCH_{pref}$<br>NR_{pref} / AR_{pref}<br>$SWITCH_{pref}$ / AR_{pref}	t_{23} = 3.28<br>t_{16} = 7.77<br>t_{13} = 5.28	0.0055<br><0.001<br><0.001	
					cocaine:<br>NR_{pref} / $SWITCH_{pref}$<br>NR_{pref} / AR_{pref}<br>$SWITCH_{pref}$ / AR_{pref}	t_{23} = 7.70<br>t_{16} = 7.01<br>t_{13} = 1.49	<0.001<br><0.001<br>0.4267	
					short-oDASS / Cocaine:<br>NR_{pref}<br>$SWITCH_{pref}$<br>AR_{pref}	t_{13}= 2.09<br>t_{10} = 6.24<br>t_{3}= 0.48	0.0465<br><0.001<br>0.6380	
7o	Re-valuation	Norm. dLight AUC CUE	NR_{pref} / No_{pref} / AR_{pref}<br>short-oDASS / Cocaine 1.25 mg/kg/inf.	Two-way ANOVA for RM	Cluster<br>Artificial Reward<br>Cluster x Artificial Reward	$F_{(2,26)}$= 5.44<br>$F_{(1,26)}$= 42.55<br>$F_{(2,26)}$= 2.18	0.0106<br><0.001<br>0.1334	η^2_p = 0.295<br>η^2_p = 0.621<br>η^2_p = 0.144
				Bonferroni	short-oDASS:<br>NR_{pref} / $SWITCH_{pref}$<br>NR_{pref} / AR_{pref}<br>$SWITCH_{pref}$ / AR_{pref}	t_{23} = 0.90<br>t_{16} = 1.61<br>t_{13} = 0.94	>0.999<br>0.3418<br>>0.999	
					cocaine:<br>NR_{pref} / $SWITCH_{pref}$<br>NR_{pref} / AR_{pref}<br>$SWITCH_{pref}$ / AR_{pref}	t_{23} = 3.40<br>t_{16} = 2.64<br>t_{13} = 0.22	0.0039<br>0.0327<br>>0.999	
					short-oDASS / Cocaine:<br>NR_{pref}:<br>$SWITCH_{pref}$<br>AR_{pref}	t_{13}= 3.57<br>t_{10} = 5.94<br>t_{3}= 2.89	0.0014<br><0.001<br>0.0078	
7p	Re-valuation	Preference score<br>Norm. dLight AUC CUE	Preference score / Norm. dLight AUC CUE (NR - short-oDASS)	Spearman's correlation		r_{27} = 0.500	0.0059	95%CI [0.151,0.737]
7q	Re-valuation	Preference score<br>Norm. dLight AUC CUE	Preference score / Norm. dLight AUC CUE (NR - cocaine)	Spearman's correlation		r_{27} = 0.560	0.0016	95%CI [0.233,0.773]
7r	Re-valuation	Preference score NR / short-oDASS<br>Preference score NR / cocaine 1.25 mg/kg/inf.	Preference score NR / short-oDASS<br>Preference score NR / cocaine 1.25 mg/kg/inf.	Spearman's correlation		r_{27} = 0.690	<0.001	95%CI [0.423,0.846]

Statistic table for Extended Data figures

Extended Data Figure	Experiment	Variable	Comparison	Test	Effect	Test result	*P*	Effect size
1b	dLight signal normalization	Peak amplitude of max. 10% ΔF/F0 (%)	Recording session 1 to 20	One-way RM ANOVA		$F_{(19,1273)}$ = 3.39	0.0019	η^2_p = 0.048
				Dunnett	Session 1/ 2	q_{67} = 0.46	>0.999	
					Session 1/ 3	q_{67} = 1.17	0.9460	
					Session 1/ 4	q_{67} = 0.29	>0.999	
					Session 1/ 5	q_{67} = 0.25	>0.999	
					Session 1/ 6	q_{67} = 0.29	>0.999	
					Session 1/ 7	q_{67} = 1.19	0.9393	
					Session 1/ 8	q_{67} = 1.29	0.8931	
					Session 1/ 9	q_{67} = 1.29	0.8938	
					Session 1/ 10	q_{67} = 1.05	0.9774	
					Session 1/ 11	q_{67} = 1.02	0.9817	
					Session 1/ 12	q_{67} = 1.80	0.5325	
					Session 1/ 13	q_{67} = 1.66	0.6458	
					Session 1/ 14	q_{67} = 2.26	0.2513	
					Session 1/ 15	q_{67} = 2.15	0.3041	
					Session 1/ 16	q_{67} = 2.77	0.0838	
					Session 1/ 17	q_{67} = 1.64	0.6615	
					Session 1/ 18	q_{67} = 2.64	0.1142	
					Session 1/ 19	q_{67} = 2.34	0.2151	
					Session 1/ 20	q_{67} = 2.21	0.2745	
1c	dLight signal normalization	Frequency of max. 10% (Hz)	Recording session 1 to 20	One-way RM ANOVA		$F_{(19,1273)}$ = 1.43	0.1966	η^2_p = 0.021
2a	Learning NR	Norm. dLight AUC Lick-5 s	Novice / Intermediate / Expert	One-way RM ANOVA		$F_{(2,74)}$ = 40.94	<0.001	η^2_p = 0.532
				Dunnett	Intermediate / Novice	q_{36}= 6.00	<0.001	
					Expert / Novice	q_{36}= 8.21	<0.001	
2a	Learning NR	Norm. dLight AUC Lick+5 s	Novice / Intermediate / Expert	One-way RM ANOVA		$F_{(2,74)}$ = 47.54	<0.001	η^2_p = 0.569
				Dunnett	Intermediate / Novice	q_{36}= 6.56	<0.001	
					Expert / Novice	q_{36}= 7.74	<0.001	
2c	Learning NR	Norm. dLight AUC Lick burst	Before / after Lick burst *per* trials	Two-way ANOVA for RM	Trial	$F_{(14,728)}$ = 15.67	<0.001	η^2_p = 0.232
					Before / After	$F_{(1,52)}$ = 1.99	0.1641	η^2_p = 0.037
					Trial x Before / After	$F_{(14,728)}$ = 30.94	<0.001	η^2_p = 0.373
				Bonferroni	Trial 1: Before / After	t_{52} = 11.94	<0.001	
					Trial 2: Before / After	t_{52} = 9.43	<0.001	
					Trial 3: Before / After	t_{52} = 7,72	<0.001	
					Trial 4: Before / After	t_{52} = 6.62	<0.001	
					Trial 5: Before / After	t_{52} = 4.27	<0.001	
					Trial 6: Before / After	t_{52} = 1.72	0.0854	
					Trial 7: Before / After	t_{52} = 2.85	0.0045	
					Trial 8: Before / After	t_{52} = 2.07	0.0384	
					Trial 9: Before / After	t_{52} = 1.74	0.0821	
					Trial 10: Before / After	t_{52} = 1.56	0.1195	
					Trial 11: Before / After	t_{52} = 4.01	<0.001	
					Trial 12: Before / After	t_{52} = 3.54	<0.001	
					Trial 13: Before / After	t_{52} = 3.49	<0.001	
					Trial 14: Before / After	t_{52} = 3.71	<0.001	
					Trial 15: Before / After	t_{52} = 3.68	<0.001	
				Bonferroni	Before: trial 1 / 2	t_{52} = 0.49	>0.999	
					Before: trial 1 / 3	t_{52} = 2.36	>0.999	
					Before: trial 1 / 4	t_{52} = 3.27	0.1198	
					Before: trial 1 / 5	t_{52} = 0.94	>0.999	
					Before: trial 6 / 7	t_{52} = 0.07	>0.999	
					Before: trial 6 / 8	t_{52} = 0.56	>0.999	
					Before: trial 6 / 9	t_{52} = 0.66	>0.999	
					Before: trial 6 / 10	t_{52} = 0.05	>0.999	
					Before: trial 11 / 12	t_{52} = 0.85	>0.999	
					Before: trial 11 / 13	t_{52} = 0.18	>0.999	
					Before: trial 11 / 14	t_{52} = 0.67	>0.999	
					Before: trial 11 / 15	t_{52} = 1.76	>0.999	
					After: trial 1 / 2	t_{52} = 2.99	0.3036	
					After: trial 1 / 3	t_{52} = 6.57	<0.001	
					After: trial 1 / 4	t_{52} = 8.58	<0.001	
					After: trial 1 / 5	t_{52} = 16.34	<0.001	
					After: trial 6 / 7	t_{52} = 1.20	>0.999	
					After: trial 6 / 8	t_{52} = 0.21	>0.999	
					After: trial 6 / 9	t_{52} = 0.64	>0.999	
					After: trial 6 / 10	t_{52} = 0.12	>0.999	
					After: trial 11 / 12	t_{52} = 0.38	>0.999	
					After: trial 11 / 13	t_{52} = 0.34	>0.999	
					After: trial 11 / 14	t_{52} = 0.38	>0.999	
					After: trial 11 / 15	t_{52} = 1.44	>0.999	
2d	NR sampling trials	Delay to press	Norm. dLight AUC CUE / Delay to press binned	One-way ANOVA		$F_{(4,317)}$ = 11.92	<0.001	η^2_p = 0.131
	expert	Norm. dLight AUC CUE		Dunnett	AUC CUE delay 0-1 / 1-3	q_{120} = 2.70	0.0251	
					AUC CUE delay 0-1 / 3-10	q_{121} = 4.37	<0.001	
					AUC CUE delay 0-1 / 10-50	q_{121} = 4.96	<0.001	
					AUC CUE delay 0-1 / 50-260	q_{117} = 6.40	<0.001	
2e	NR sampling trials expert	Norm. dLight AUC CUE	Hit / Miss trials	Paired *t*-test	Two-tailed	t_{34} = 2.13	0.0408	
2f	short-oDASS trials	Delay to press	Norm. dLight AUC CUE / Delay to press binned	One-way ANOVA		$F_{(4,307)}$ = 10.66	<0.001	η^2_p = 0.122
	expert	Norm. dLight AUC CUE		Dunnett	AUC CUE delay 0-1 / 1-3	q_{106} = 3.97	<0.001	
					AUC CUE delay 0-1 / 3-10	q_{109} = 4.85	<0.001	
					AUC CUE delay 0-1 / 10-50	q_{109} = 5.03	<0.001	
					AUC CUE delay 0-1 / 50-260	q_{109} = 6.28	<0.001	
2g	short-oDASS trials expert	Norm. dLight AUC CUE	Hit / Miss trials	Paired *t*-test	Two-tailed	t_{43} = 2.26	0.0287	MD = -0.099, 95%CI [-0.187,-0.011]
2h	NR omission	Norm. dLight AUC CUE	Rewarded / Not rewarded	Paired *t*-test	Two-tailed	t_{11} = 0.54	0.6017	MD = 0.028, 95%CI [-0.087,0.143]
		Norm. dLight AUC Le	Rewarded / Not rewarded	Paired *t*-test	Two-tailed	t_{11} = 1.15	0.2759	MD = 0.055, 95%CI [-0.050,-0.160]
		Norm. dLight AUC Press-Rd	Rewarded / Not rewarded	Paired *t*-test	Two-tailed	t_{11} = 3.07	0.0108	MD = 0.331, 95%CI [0.093,0.569]
		Norm. dLight AUC Rd+1 s	Rewarded / Not rewarded	Paired *t*-test	Two-tailed	t_{11} = 1.14	0.2802	MD = 0.044, 95%CI [-0.042,0.131]
		Norm. dLight AUC Rd_{full}	Rewarded / Not rewarded	Paired *t*-test	Two-tailed	t_{11} = 3.23	0.0080	MD = -4.528, 95%CI [-7.612,-1.444]
2i	Short-oDASS omission	Norm. dLight AUC CUE	Rewarded / Not rewarded	Paired *t*-test	Two-tailed	t_{6} = 0.62	0.5579	MD = -0.079, 95%CI [-0.393,0.234]
		Norm. dLight AUC Le	Rewarded / Not rewarded	Paired *t*-test	Two-tailed	t_{6} = 0.73	0.4931	MD = -0.065, 95%CI [-0.282,0.153]
		Norm. dLight AUC Press-Rd	Rewarded / Not rewarded	Paired *t*-test	Two-tailed	t_{6} = 0.02	0.9835	MD = -0.008, 95%CI [-0.874,0.859]
		Norm. dLight AUC Rd+1 s	Rewarded / Not rewarded	Paired *t*-test	Two-tailed	t_{6} = 7.29	0.0003	MD = -1.269, 95%CI [-1.695,-0.843]
		Norm. dLight AUC Rd_{full}	Rewarded / Not rewarded	Paired *t*-test	Two-tailed	t_{6} = 3.07	0.0220	MD = -10.41, 95%CI [-18.71,-2.105]

Extended Data Figure	Experiment	Variable	Comparison	Test	Effect	Test result	*P*	Effect size
2l	Choice NR / short-oDASS	Preference score<br>Norm. dLight AUC CUE (NR)	Preference score / Norm. dLight AUC CUE (NR)	Spearman's correlation		$r_{66} = 0.481$	<0.001	95%CI [0.267,0.650]
2l	Choice NR / short-oDASS	Preference score<br>Norm. dLight AUC CUE (oDASS)	Preference score / Norm. dLight AUC CUE (oDASS)	Spearman's correlation		$r_{66} = -0.406$	<0.001	95%CI [-0.593,-0.179]
3a	Learning NR	Norm. dLight AUC CUE<br>Norm. dLight AUC Rd_{full}	Norm. dLight AUC CUE (expert) / Norm. dLight AUC Rd_{full} Max. (novice)	Spearman's correlation		$r_{78} = 0.058$	0.6108	95%CI [-0.171,0.280]
3b	Learning short-oDASS	Norm. dLight AUC CUE<br>Norm. dLight AUC Rd_{full}	Norm. dLight AUC CUE (expert) / Norm. dLight AUC Rd_{full} Max. (novice)	Spearman's correlation		$r_{45} = -0.007$	0.9612	95%CI [-0.302,0.289]
3c	Learning long-oDASS	Norm. dLight AUC CUE<br>Norm. dLight AUC Rd_{full}	Norm. dLight AUC CUE (expert) / Norm. dLight AUC Rd_{full} Max. (novice)	Spearman's correlation		$r_{11} = 0.159$	0.6040	95%CI [-0.444,0.663]
4d	Re-valuation	Preference score	short-oDASS / long-oDASS	Paired *t*-test	Two-tailed	$t_{26} = 7.93$	<0.001	MD = -0.660, 95%CI [-0.831,-0.489]
4h	Re-valuation	Norm. dLight AUC Rd_{full} (NR - oDASS)	NR_{pref} / $Switch_{pref}$ / $oDASS_{pref}$<br>short-oDASS / long-oDASS	Two-way ANOVA for RM	Cluster<br>oDASS protocol<br>Cluster x oDASS protocol	$F_{(2,24)} = 2.87$<br>$F_{(1,24)} = 53.89$<br>$F_{(2,24)} = 2.82$	0.0763<br><0.001<br>0.0796	$\eta^2_p = 0.193$<br>$\eta^2_p = 0.692$<br>$\eta^2_p = 0.190$
				Bonferroni	short-oDASS:<br>NR_{pref} / $Switch_{pref}$<br>NR_{pref} / $oDASS_{pref}$<br>$Switch_{pref}$ / $oDASS_{pref}$	 $t_{18} = 0.18$<br>$t_{11} = 0.17$<br>$t_{19} = 0.39$	 >0.999<br>>0.999<br>>0.999	
					long-oDASS:<br>NR_{pref} / $Switch_{pref.}$<br>NR_{pref} / $oDASS_{pref}$<br>$Switch_{pref}$ / $oDASS_{pref}$	 $t_{18} = 2.29$<br>$t_{11} = 0.52$<br>$t_{19} = 3.04$	 0.0786<br>>0.999<br>0.0113	
					short- / long-oDASS:<br>NR_{pref}<br>$Switch_{pref}$<br>$oDASS_{pref}$	 $t_{5} = 3.15$<br>$t_{13} = 7.90$<br>$t_{6} = 3.00$	 0.0043<br><0.001<br>0.0063	
5d	Extended oDASS training	Preference score	Expert /over-trained short-oDASS	Paired *t*-test	Two-tailed	$t_{28} = 0.68$	0.4992	MD = 0.051, 95%CI [-0.102,0.204]
5h	Extended oDASS training	Norm. dLight AUC Rd_{full} (NR-oDASS)	NR_{pref} /No_{pref}<br>Expert /over-trained short-oDASS	Two-way ANOVA for RM	Cluster<br>Extended oDASS training<br>Cluster x Extended oDASS training	$F_{(1,27)} = 1.15$<br>$F_{(1,27)} = 0.04$<br>$F_{(1,27)} = 1.72$	0.2927<br>0.8342<br>0.2004	$\eta^2_p = 0.041$<br>$\eta^2_p = 0.002$<br>$\eta^2_p = 0.060$
6a	Choice NR / Cocaine 1.25 mg/kg/inf.	Norm. dLight AUC CUE	Hit / Miss trials cocaine sampling	Paired *t*-test	Two-tailed	$t_{31} = 5.10$	<0.001	MD = -0.357, 95%CI [-0.500,-0.214]
6a	Choice NR / Cocaine 1.25 mg/kg/inf.	Norm. dLight AUC CUE	Hit / Miss trials NR sampling	Paired *t*-test	Two-tailed	$t_{37} = 0.45$	0.6553	MD = -0.023, 95%CI [-0.129,0.821]
6e	Cocaine 1.25 mg/kg/inf.	Norm. dLight AUC Rd_{full} (NR-Coc)	NR_{pref} / $Cocaine_{pref}$	Unpaired *t*-test	Two-tailed	$t_{47} = 1.14$	0.2592	MD = -20.85, 95%CI [-57.57,15.87]
8a	Learning NR	Norm. dLight AUC Lick-5 s	Novice / Intermediate / Expert	One-way RM ANOVA		$F_{(2,24)} = 17.97$	0.0008	$\eta^2_p = 0.600$
				Dunnett	Intermediate / Novice<br>Expert / Novice	$q_{12} = 4.32$<br>$q_{12} = 4.25$	0.0019<br>0.0021	
8a	Sampling NR expert	Norm. dLight AUC Lick+5 s	Novice / Intermediate / Expert	One-way RM ANOVA		$F_{(2,24)} = 9.84$	0.0063	$\eta^2_p = 0.451$
				Dunnett	Intermediate / Novice<br>Expert / Novice	$q_{12} = 3.28$<br>$q_{12} = 3.13$	0.0122<br>0.0160	
8b	Sampling NR expert	Delay to press<br><br>Norm. dLight AUC CUE	Norm. dLight AUC CUE / Delay to<br><br>press binned	One-way ANOVA		$F_{(4,247)} = 5.19$	0.0005	$\eta^2_p = 0.078$
				Dunnett	delay 0-1 / 1-3<br>delay 0-1 / 3-10<br>delay 0-1 / 10-50<br>delay 0-1 / 50-260	$q_{92} = 1.81$<br>$q_{92} = 3.26$<br>$q_{92} = 3.47$<br>$q_{88} = 4.00$	0.2046<br>0.0046<br>0.0022<br><0.001	
Ext. Data F8c	Sampling trials for NR expert	Norm. dLight AUC CUE	Hit / Miss trials	Paired *t*-test	Two-tailed	$t_{16} = 0.61$	0.5480	MD = -0.058, 95%CI [-0.259,0.143]
Ext. Data F8d	Sampling long-oDASS<br><br>expert	Delay to press<br><br>Norm. dLight AUC CUE	Norm. dLight AUC CUE / Delay to<br><br>press binned	One-way ANOVA		$F_{(4,256)} = 3.05$	0.0177	$\eta^2_p = 0.045$
				Dunnett	delay 0-1 / 1-3<br>delay 0-1 / 3-10<br>delay 0-1 / 10-50<br>delay 0-1 / 50-260	$q_{104} = 0.26$<br>$q_{104} = 1.44$<br>$q_{104} = 1.94$<br>$q_{97} = 3.00$	0.9969<br>0.4078<br>0.1654<br>0.0108	
8e	Sampling long-oDASS expert	Norm. dLight AUC CUE	Hit / Miss trials	Paired *t*-test	Two-tailed	$t_{13} = 2.86$	0.0135	MD = -0.429, 95%CI [-0.754,-0.105]
8g	Long-oDASS omission	Norm. dLight AUC CUE	Rewarded / Not rewarded	Paired *t*-test	Two-tailed	$t_{11} = 1.20$	0.2552	MD = 0.087, 95%CI [-0.072,0.246]
		Norm. dLight AUC Le	Rewarded / Not rewarded	Paired *t*-test	Two-tailed	$t_{11} = 0.99$	0.3428	MD = 0.044, 95%CI [-0.053,0.141]
		Norm. dLight AUC Press-Rd	Rewarded / Not rewarded	Paired *t*-test	Two-tailed	$t_{11} = 2.56$	0.0268	MD = 0.411, 95%CI [0.057,0.764]
		Norm. dLight AUC Rd+1 s	Rewarded / Not rewarded	Paired *t*-test	Two-tailed	$t_{11} = 10.70$	<0.001	MD = -1.355, 95%CI [-1.634,-1.076]
		Norm. dLight AUC Rd_{full}	Rewarded / Not rewarded	Paired *t*-test	Two-tailed	$t_{11} = 5.82$	<0.001	MD = -65.31, 95%CI [-89.99,-40.63]
8h	Devaluation	Preference score	No Punishment Risk / PR	Paired *t*-test	Two-tailed	$t_{26} = 7.95$	<0.001	MD = 0.802, 95%CI [-0.595,1.009]
8n	Modality of the CUE during long-oDASS learning	Norm. dLight AUC CUE (NR - long-oDASS)	CUE modality oDASS visual and NR auditory / oDASS auditory and NR visual	Unpaired *t*-test	Two-tailed	$t_{25} = 0.23$	0.8209	MD = 0.052, 95%CI [-0.412,-0.516]
8p	Devaluation	Norm. dLight AUC Rd_{full} (NR - oDASS)	$Switch_{pref}$ / $oDASS_{pref}$	Two-way ANOVA for RM	Cluster<br>PR<br>Cluster x PR	$F_{(1,25)} = 1.11$<br>$F_{(1,25)} = 0.35$<br>$F_{(1,25)} = 3.48$	0.3031<br>0.5591<br>0.0738	$\eta^2_p = 0.042$<br>$\eta^2_p = 0.014$<br>$\eta^2_p = 0.122$
9h	Choice NR / Cocaine 0.75 mg/kg/inf.	Norm. dLight AUC Rd_{full} (NR-Cocaine)	NR_{pref} / No_{pref}	Unpaired *t*-test	Two-tailed	$t_{23} = 2.38$	0.0263	MD = -47.94, 95%CI [-89.70,-6.182]
9i	Sampling Cocaine 0.75 mg/kg/inf. expert	Norm. dLight AUC CUE	Hit / Miss trials	Paired *t*-test		$t_{12} = 2.30$	0.0402	MD = -0.337, 95%CI [-0.656,-0.018]
9i	Sampling NR expert	Norm. dLight AUC CUE	Hit / Miss trials	Paired *t*-test		$t_{15} = 3.15$	0.0065	MD = -0.344, 95%CI [-0.576,-0.111]
9j	Re-valuation	Preference score	short-oDASS / Cocaine 1.25 mg/kg/inf.	Paired *t*-test	Two-tailed	$t_{28} = 4.60$	<0.001	MD = -0.316, 95%CI [-0.457,-0.176]
9q	Re-valuation	Norm. dLight AUC Rd_{full}	NR_{pref} / No_{pref} / AR_{pref}<br>short-oDASS / Cocaine 1.25 mg/kg/inf.	Two-way ANOVA for RM	Cluster<br>Artificial Reward<br>Cluster x Artificial Reward	$F_{(2,26)} = 0.42$<br>$F_{(1,26)} = 41.48$<br>$F_{(2,26)} = 0.62$	0.6595<br><0.001<br>0.5438	$\eta^2_p = 0.032$<br>$\eta^2_p = 0.615$<br>$\eta^2_p = 0.046$
				Bonferroni	short-oDASS:<br>NR_{pref} / $SWITCH_{pref}$<br>NR_{pref} / AR_{pref}<br>$SWITCH_{pref}$ / AR_{pref}	 $t_{23} = 0.11$<br>$t_{16} = 0.21$<br>$t_{13} = 0.12$	 >0.999<br>>0.999<br>>0.999	
					cocaine:<br>NR_{pref} / No_{pref}<br>NR_{pref} / AR_{pref}<br>$SWITCH_{pref}$ / AR_{pref}	 $t_{23} = 1.41$<br>$t_{16} = 0.27$<br>$t_{13} = 0.71$	 0.4945<br>>0.999<br>>0.999	
					NR_{pref}: short-oDASS / Cocaine<br>$SWITCH_{pref}$: short-oDASS / Cocaine<br>AR_{pref}: short-oDASS / Cocaine	$t_{13} = 4.35$<br>$t_{10} = 5.35$<br>$t_{3} = 2.72$	0.0002<br><0.001<br>0.0115	

Extended Data Figure	Experiment	Variable	Comparison	Test	Effect	Test result	P	Effect size
9r	Re-valuation	Preference score<br>Norm. dLight AUC	Preference score / Norm. dLight AUC Rd_{full} (NR - short-oDASS)	Spearman's correlation		r_{27} = -0.010	0.9595	95%CI [-0.385,0.368]
9r	Re-valuation	Preference score<br>Norm. dLight AUC	Preference score / Norm. dLight AUC Rd_{full} (NR - cocaine)	Spearman's correlation		r_{27} = 0.091	0.6381	95%CI [-0.295,0.452]
10a	Choice NR / short-oDASS	Preference score	NR_{pref} / $oDASS_{pref}$<br>Female / Male	Two-way ANOVA	Cluster<br>Sex<br>Cluster x Sex	$F_{(1,64)}$ = 108.5<br>$F_{(1,64)}$ = 0.05<br>$F_{(1,64)}$ = 0.03	<0.001<br>0.8316<br>0.8573	η^2_p = 0.629<br>η^2_p = 0.001<br>η^2_p = 0.001
				Uncorrected Fisher's LSD	Female: NR_{pref} / $oDASS_{pref}$<br>Male: NR_{pref} / $oDASS_{pref}$<br>NR_{pref}: Female / Male<br>$oDASS_{pref}$: Female / Male	t_{35} = 9.04<br>t_{29} = 6.44<br>t_{52} = 0.04<br>t_{12} = 0.22	<0.001<br><0.001<br>0.9686<br>0.8290	
	Choice NR / short-oDASS	Norm. dLight AUC CUE	NR_{pref} / $oDASS_{pref}$<br>Female / Male	Two-way ANOVA	Cluster<br>Sex<br>Cluster x Sex	$F_{(1,64)}$ = 23.51<br>$F_{(1,64)}$ = 0.00<br>$F_{(1,64)}$ = 0.01	<0.001<br>0.9356<br>0.9159	η^2_p = 0.269<br>η^2_p = 0.0001<br>η^2_p = 0.0002
				Uncorrected Fisher's LSD	Female: NR_{pref} / $oDASS_{pref}$<br>Male: NR_{pref} / $oDASS_{pref}$<br>NR_{pref}: Female / Male<br>$oDASS_{pref}$: Female / Male	t_{35} = 4.17<br>t_{29} = 3.01<br>t_{52} = 0.03<br>t_{12} = 0.10	<0.001<br>0.0037<br>0.9763<br>0.9182	
	Choice NR / short-oDASS in Female	Preference score<br>Norm. dLight AUC	Preference score / Norm. dLight AUC CUE (NR - short-oDASS)	Spearman's correlation		r_{35} = 0.645	<0.001	95%CI [0.397,0.805]
	Choice NR / short-oDASS in Male	Preference score<br>Norm. dLight AUC	Preference score / Norm. dLight AUC CUE (NR - short-oDASS)	Spearman's correlation		r_{29} = 0.361	0.0458	95%CI [0.003,0.641]
10b	Choice NR / long-oDASS	Preference score	NR_{pref} / $oDASS_{pref}$<br>Female / Male	Two-way ANOVA	Cluster<br>Sex<br>Cluster x Sex	$F_{(1,50)}$ = 105.6<br>$F_{(1,50)}$ = 0.17<br>$F_{(1,50)}$ = 0.14	<0.001<br>0.6831<br>0.7131	η^2_p = 0.679<br>η^2_p = 0.003<br>η^2_p = 0.003
				Uncorrected Fisher's LSD	Female: NR_{pref} / $oDASS_{pref}$<br>Male: NR_{pref} / $oDASS_{pref}$<br>NR_{pref}: Female / Male<br>$oDASS_{pref}$: Female / Male	t_{27} = 8.60<br>t_{23} = 6.51<br>t_{8} = 0.43<br>t_{42} = 0.05	<0.001<br><0.001<br>0.6721<br>0.9595	
	Choice NR / long-oDASS	Norm. dLight AUC CUE	NR_{pref} / $oDASS_{pref}$<br>Female / Male	Two-way ANOVA	Cluster<br>Sex<br>Cluster x Sex	$F_{(1,50)}$ = 6.58<br>$F_{(1,50)}$ = 1.12<br>$F_{(1,50)}$ = 0.15	0.0134<br>0.2955<br>0.6973	η^2_p = 0.116<br>η^2_p = 0.022<br>η^2_p = 0.003
				Uncorrected Fisher's LSD	Female: NR_{pref} / $oDASS_{pref}$<br>Male: NR_{pref} / $oDASS_{pref}$<br>NR_{pref}: Female / Male<br>$oDASS_{pref}$: Female / Male	t_{27} = 2.57<br>t_{23} = 1.33<br>t_{8} = 0.79<br>t_{42} = 0.83	0.0134<br>0.1896<br>0.4330<br>0.4095	
	Choice NR / long-oDASS in Female	Preference score<br>Norm. dLight AUC	Preference score / Norm. dLight AUC CUE (NR - long-oDASS)	Spearman's correlation		r_{27} = 0.407	0.0285	95%CI [0.036,0.679]
	Choice NR / long-oDASS in Male	Preference score<br>Norm. dLight AUC	Preference score / Norm. dLight AUC CUE (NR - long-oDASS)	Spearman's correlation		r_{23} = 0.255	0.2193	95%CI [-0.168,0.594]
10c	Choice NR / long-oDASS with punishment risk	Preference score	NR_{pref} / $oDASS_{pref}$<br>Female / Male	Two-way ANOVA	Cluster<br>Sex<br>Cluster x Sex	$F_{(1,23)}$ = 121.4<br>$F_{(1,23)}$ = 0.16<br>$F_{(1,23)}$ = 0.002	<0.001<br>0.6947<br>0.9649	η^2_p = 0.841<br>η^2_p = 0.007<br>η^2_p = 0.0001
				Uncorrected Fisher's LSD	Female: $Switch_{pref}$ / $oDASS_{pref}$<br>Male: $Switch_{pref}$ / $oDASS_{pref}$<br>$Switch_{pref}$: Female / Male<br>$oDASS_{pref}$: Female / Male	t_{11} = 7.10<br>t_{12} = 8.72<br>t_{16} = 0.40<br>t_{7} = 0.21	<0.001<br><0.001<br>0.6952<br>0.8336	
	Choice NR / long-oDASS with punishment risk	Norm. dLight AUC CUE	NR_{pref} / $oDASS_{pref}$<br>Female / Male	Two-way ANOVA	Cluster<br>Sex<br>Cluster x Sex	$F_{(1,23)}$ = 39.59<br>$F_{(1,23)}$ = 0.85<br>$F_{(1,23)}$ = 0.06	<0.001<br>0.3654<br>0.8092	η^2_p = 0.633<br>η^2_p = 0.036<br>η^2_p = 0.003
				Uncorrected Fisher's LSD	Female: $Switch_{pref}$ / $oDASS_{pref}$<br>Male: $Switch_{pref}$ / $oDASS_{pref}$<br>$Switch_{pref}$: Female / Male<br>$oDASS_{pref}$: Female / Male	t_{11} = 4.23<br>t_{12} = 4.77<br>t_{16} = 0.61<br>t_{7} = 0.70	<0.001<br><0.001<br>0.5481<br>0.4891	
	Choice NR / long-oDASS with punishment risk in Female	Preference score<br>Norm. dLight AUC	Preference score / Norm. dLight AUC CUE (NR - long-oDASS with PR)	Spearman's correlation		r_{11} = 0.637	0.0221	95%CI [0.115,0.884]
	Choice NR / long-oDASS with punishment risk in Male	Preference score<br>Norm. dLight AUC	Preference score / Norm. dLight AUC CUE (NR - long-oDASS with PR)	Spearman's correlation		r_{12} = 0.657	0.0128	95%CI [0.177,0.885]
10d	Choice NR / Cocaine 1.25 mg/kg/inf.	Preference score	NR_{pref} / No_{pref}<br>Female / Male	Two-way ANOVA	Cluster<br>Sex<br>Cluster x Sex	$F_{(1,45)}$ = 160.1<br>$F_{(1,45)}$ = 0.54<br>$F_{(1,45)}$ = 6.45	<0.001<br>0.4640<br>0.0146	η^2_p = 0.781<br>η^2_p = 0.012<br>η^2_p = 0.125
				Uncorrected Fisher's LSD	Female: NR_{pref} / COC_{pref}<br>Male: NR_{pref} / COC_{pref}<br>NR_{pref}: Female / Male<br>COC_{pref}: Female / Male	t_{21} = 10.31<br>t_{24} = 7.48<br>t_{18} = 1.21<br>t_{25} = 2.47	<0.001<br><0.001<br>0.2346<br>0.0174	
	Choice NR / Cocaine 1.25 mg/kg/inf.	Norm. dLight AUC CUE	NR_{pref} / No_{pref}<br>Female / Male	Two-way ANOVA	Cluster<br>Sex<br>Cluster x Sex	$F_{(1,45)}$ = 20.53<br>$F_{(1,45)}$ = 0.00<br>$F_{(1,45)}$ = 4.73	<0.001<br>0.9533<br>0.0349	η^2_p = 0.313<br>η^2_p = 0.0001<br>η^2_p = 0.0006
				Uncorrected Fisher's LSD	Female: NR_{pref} / COC_{pref}<br>Male: NR_{pref} / COC_{pref}<br>NR_{pref}: Female / Male<br>COC_{pref}: Female / Male	t_{21}= 4.55<br>t_{24} = 1.74<br>t_{20} = 1.49<br>t_{25} = 1.59	<0.001<br>0.0884<br>0.1421<br>0.1180	
	Choice NR / Cocaine 1.25 mg/kg/inf. in Female	Preference score<br>Norm. dLight AUC	Preference score / Norm. dLight AUC CUE (NR - Cocaine)	Spearman's correlation		r_{21} = 0.674	<0.001	95%CI [0.352,0.854]
	Choice NR / Cocaine 1.25 mg/kg/inf. in Male	Preference score<br>Norm. dLight AUC	Preference score / Norm. dLight AUC CUE (NR - Cocaine)	Spearman's correlation		r_{24} = 0.388	0.05	95%CI [-0.11,0.681]
