# Supplementary material for: Conditioned accumbal dopamine transients forecast individual preference for drug versus natural rewards and compulsive behavior
Source: Nat Neurosci. 2026 Jun 26;29(8):1905–19. doi: 10.1038/s41593-026-02331-y (PMC13433266; doi:10.1038/s41593-026-02331-y)
Supplement: Supplementary file 2 [file 41593_2026_2331_MOESM2_ESM.pdf]

nature portfolio

Corresponding author(s): Christian.Luscher@unige.ch

Last updated by author(s): Apr 23, 2026

Reporting Summary

Nature Portfolio wishes to improve the reproducibility of the work that we publish. This form provides structure for consistency and transparency in reporting. For further information on Nature Portfolio policies, see our Editorial Policies and the Editorial Policy Checklist.

Statistics

For all statistical analyses, confirm that the following items are present in the figure legend, table legend, main text, or Methods section.

n/a	Confirmed	
☐	☒	The exact sample size (*n*) for each experimental group/condition, given as a discrete number and unit of measurement
☐	☒	A statement on whether measurements were taken from distinct samples or whether the same sample was measured repeatedly
☐	☒	The statistical test(s) used AND whether they are one- or two-sided<br>*Only common tests should be described solely by name; describe more complex techniques in the Methods section.*
☒	☐	A description of all covariates tested
☐	☒	A description of any assumptions or corrections, such as tests of normality and adjustment for multiple comparisons
☐	☒	A full description of the statistical parameters including central tendency (e.g. means) or other basic estimates (e.g. regression coefficient) AND variation (e.g. standard deviation) or associated estimates of uncertainty (e.g. confidence intervals)
☐	☒	For null hypothesis testing, the test statistic (e.g. *F*, *t*, *r*) with confidence intervals, effect sizes, degrees of freedom and *P* value noted<br>*Give P values as exact values whenever suitable.*
☐	☒	For Bayesian analysis, information on the choice of priors and Markov chain Monte Carlo settings
☐	☒	For hierarchical and complex designs, identification of the appropriate level for tests and full reporting of outcomes
☐	☒	Estimates of effect sizes (e.g. Cohen's *d*, Pearson's *r*), indicating how they were calculated

Our web collection on statistics for biologists contains articles on many of the points above.

Software and code

Policy information about availability of computer code

Data collection	Behavior data were collected with MED-PC 5.1 (MedAssociate)<br>Photometry data were collected with TDT Synapse v.98-528 (Ticker Davis)
Data analysis	Customed made codes written in MATLAB (R2023b) for photometry data analysis are available on Zenodo DOI: 10.5281/zenodo.15463589<br>Graphs were madde with Igor 9.05<br>Statistics were made with GraphPad Prism11

For manuscripts utilizing custom algorithms or software that are central to the research but not yet described in published literature, software must be made available to editors and reviewers. We strongly encourage code deposition in a community repository (e.g. GitHub). See the Nature Portfolio guidelines for submitting code & software for further information.

Data

Policy information about availability of data

All manuscripts must include a data availability statement. This statement should provide the following information, where applicable:

- Accession codes, unique identifiers, or web links for publicly available datasets
- A description of any restrictions on data availability
- For clinical datasets or third party data, please ensure that the statement adheres to our policy

Data will be available on Zenodo (DOI: 10.5281/zenodo.15463589)

Research involving human participants, their data, or biological material

Policy information about studies with human participants or human data. See also policy information about sex, gender (identity/presentation), and sexual orientation and race, ethnicity and racism.

Reporting on sex and gender	N/A
Reporting on race, ethnicity, or other socially relevant groupings	N/A
Population characteristics	N/A
Recruitment	N/A
Ethics oversight	N/A

Note that full information on the approval of the study protocol must also be provided in the manuscript.

Field-specific reporting

Please select the one below that is the best fit for your research. If you are not sure, read the appropriate sections before making your selection.

☒ Life sciences ☐ Behavioural & social sciences ☐ Ecological, evolutionary & environmental sciences

For a reference copy of the document with all sections, see nature.com/documents/nr-reporting-summary-flat.pdf

Life sciences study design

All studies must disclose on these points even when the disclosure is negative.

Sample size	Sample size was determined according to previous experience in Lüscher Laboratory or by Monte Carlo Simulation. Large cohorts were used in each experiment to evaluate individual variability that is inherent to addiction behaviors.
Data exclusions	Mice with low infection levels (confirmed post-hoc with histology) were not self-stimulated and were excluded.
Replication	Choice behavior of each mouse was replicated several time (48-80 times) and presented as 3 periods (early-mid-late). Choice stability was observed in each experiment. Photometry recordings were performed during several trials.
Randomization	CUE modality (auditory and visual) was randomly assigned to the artificial and natural rewards for each mouse.
Blinding	Correlation between DA transients and choice behavior were made after experimental recordings, during which the experimentater was blind to the futur mouse cluster.

Reporting for specific materials, systems and methods

We require information from authors about some types of materials, experimental systems and methods used in many studies. Here, indicate whether each material, system or method listed is relevant to your study. If you are not sure if a list item applies to your research, read the appropriate section before selecting a response.

Materials & experimental systems

n/a	Involved in the study	
☐	☒	Antibodies
☒	☐	Eukaryotic cell lines
☒	☐	Palaeontology and archaeology
☐	☒	Animals and other organisms
☒	☐	Clinical data
☒	☐	Dual use research of concern
☒	☐	Plants

Methods

n/a	Involved in the study	
☒	☐	ChIP-seq
☒	☐	Flow cytometry
☒	☐	MRI-based neuroimaging

Antibodies

Antibodies used	primary antibody (rabbit polyclonal anti-GFP, Invitrogen A11122, lot 2481666, diluted 1:500) and the secondary antibody (donkey

Antibodies used	anti-rabbit Alexa 488, Invitrogen A21206, lot 19277937, diluted 1:500)
Validation	Antibody was validated in our previous publications DOI: 10.1038/s41386-022-01429-8 and DOI: 10.1038/s41586-018-0789-4

Animals and other research organisms

Policy information about studies involving animals; ARRIVE guidelines recommended for reporting animal research, and Sex and Gender in Research

Laboratory animals	Mice DAT B6.SJL-Slc6a3tm1.1(cer)Bkmn/J, also known as DAT-IRES-Cre. The mice were single housed after surgery. All animals were kept in a temperature- and humidity-controlled environment with an inverted 12 h light / 12 h dark cycle (light off at 07:00AM).
Wild animals	N/A
Reporting on sex	Both male and female underwent the experiments. Extended Data Figure 10 reports data as a function of sex.
Field-collected samples	N/A
Ethics oversight	All procedures were approved by the Institutional Animal Care and Use Committee of the University of Geneva and by the animal welfare committee of the Cantonal of Geneva, in accordance with Swiss lawAll procedures were approved by the Institutional Animal Care and Use Committee of the University of Geneva and by the animal welfare committee of the Cantonal of Geneva, in accordance with Swiss law.

Note that full information on the approval of the study protocol must also be provided in the manuscript.

Plants

Seed stocks	N/A
Novel plant genotypes	N/A
Authentication	N/A
